# Long‐Term and Regional‐Scale Data Reveal Divergent Trends of Different Climate Variables on Fish Body Size Over 75 Years

**DOI:** 10.1111/gcb.70584

**Published:** 2025-11-05

**Authors:** Peter J. Flood, Kaitlin E. Schiller, Katelyn B. S. King, Andrew D. Runyon, Kevin E. Wehrly, Karen M. Alofs

**Affiliations:** ^1^ School for Environment and Sustainability University of Michigan Ann Arbor Michigan USA; ^2^ Michigan Department of Natural Resources Institute for Fisheries Research Ann Arbor Michigan USA

**Keywords:** body size, climate change, growth, long‐term data, machine learning, temperature size rule

## Abstract

Across many ecto‐ and endothermic organisms, climate change has induced a general shift towards smaller body sizes. Several existing hypotheses (e.g., Temperature Size Rule—TSR, metabolic theory) contribute to our understanding of climate‐driven changes in body size. However, empirical support for climate‐induced reductions in body size is mixed with some species growing larger under warmer temperatures, and underlying mechanisms are under debate. To address these inconsistencies, we used Bayesian hierarchical modeling to determine if mean length‐at‐age (proxy for growth) changed from 1945 to 2020 for age classes of 13 freshwater fish species. Then, we used boosted regression trees (BRTs) to disentangle the impacts of climate change on growth from other environmental factors. Hierarchical modeling revealed that 37% of age classes were decreasing in mean length through time (69% were qualitatively decreasing). BRTs demonstrated that growing degree days and mean annual surface water temperature had varying effects on growth. For cold‐and cool‐water adapted fishes, length‐at‐age usually increased as a function of degree days but decreased as a function of surface temperature. Warm‐water adapted fishes, however, typically decreased in response to both degree days and surface temperature. The direction of change in length‐at‐age as a function of surface temperature corresponded to the direction of change over time for 62% (8/13) of species. Overall, we found widespread decreases in length, including age classes from all thermal guilds and juveniles (contrary to TSR assumptions). Mixed results in prior literature may result from choosing different variables to represent climate warming and/or not considering age‐specific length responses. When specific climate variables and age are considered, climate change effects on body size may be more predictable at large temporal and spatial scales than previously thought. Continued decreases in length for the youngest and oldest fishes could lead to biodiversity loss and diminished ecosystem functions and services.

## Graphical abstract

This research demonstrated that over the past 75 years many ages of multiple species of freshwater fishes were decreasing in length through time and with increasing water temperatures. The largest decreases were found in the youngest and oldest fishes, which are disproportionately important for healthy populations and ecosystems.

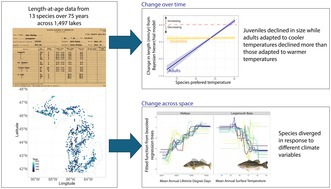

## 1 Introduction

Body size mediates many ecological and physiological processes and is predicted to decrease for many taxa around the globe with climate warming (Ahti et al. 2020; Gardner et al. 2011; Peters 1983). Widespread shifts in body size in populations and communities can negatively impact human food supply and related economics through cascading food‐web effects on crops (both directly and indirectly through pollinators) and harvested animals (Audzijonyte et al. 2013; Bartomeus et al. 2013; Martins et al. 2023). Decreasing body size may be particularly important in aquatic ecosystems as evidence suggests that climate‐related reductions in body size are stronger in aquatic than in terrestrial organisms (Forster et al. 2012). For fishes, decreasing body size can lead to lower fecundity, diminished dispersal ability, elevated competitive effects and predation risk, and altered population dynamics (Woodward et al. 2005). Moreover, fish body‐size reductions have negative consequences for ecosystem functions and services by reducing consumer‐mediated nutrient transport and decreasing fishing opportunities (Oke et al. 2020). These wide‐ranging impacts of decreasing body size across levels of ecological organization (from individuals to populations, communities, and ecosystems) highlight the importance of understanding the impacts of climate on body size and disentangling climate effects from other potential drivers (e.g., land use changes, abiotic differences across the landscape, variable harvest from commercial, recreational, and subsistence fishing, introduced species, and nutrient addition).

**FIGURE 1:**
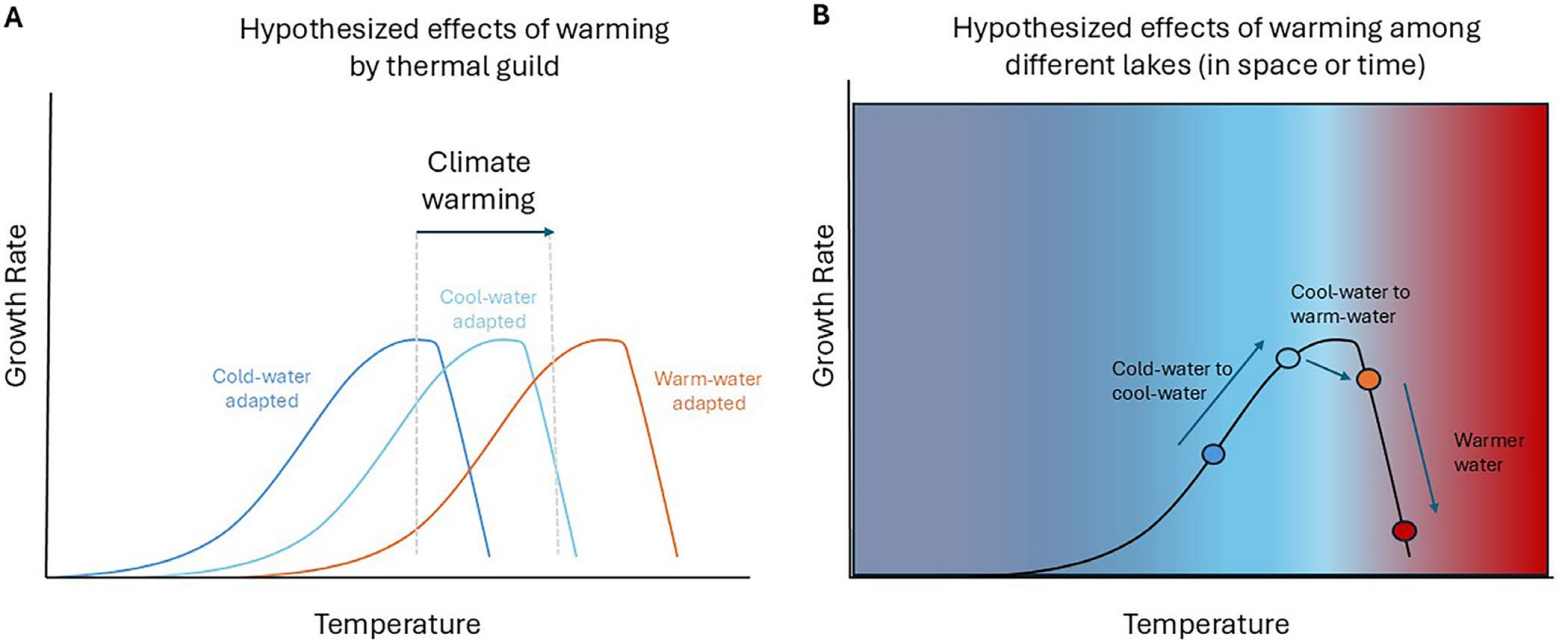
Hypothesized effects of climate warming on (A) fishes of different thermal guilds and on (B) among different lakes either changing through time or thermal shifts in different lakes across space at the same time (this example represents predicted responses for a cool‐water adapted fish). For fishes of different thermal guilds, warming may increase, have minimal impact, or decrease growth depending on the temperature preferences of that fish. As warming continues and lake water temperatures increase, growth should change predictably for a given species based on the thermal regime of that lake. Similarly, when comparing lakes with different thermal regimes within a single year across space, those differences could be used to predict future changes through time.

As ectotherms, fish growth is dependent on ambient temperature and existing hypotheses can inform our understanding of climate‐related changes in body size. The Temperature Size Rule (TSR) states that under increased temperatures, ectotherms will have elevated juvenile growth rates, yet smaller adult body sizes (Atkinson 1994; Verberk et al. 2021). In contrast, metabolic theory suggests that metabolic rates scale with mass and increase exponentially with temperature, leading to increased growth when caloric demands are met (Brown et al. 2004). Finally, impacts of warming on growth may vary with species' thermal traits and adaptations (Figure 1; Magozzi and Calosi 2015). Empirical evidence has shown mixed support for these hypotheses and climate‐driven decreases in body size (Audzijonyte et al. 2020; Warne et al. 2024). One notable exception is observed increases in body size for several species of North American salmonids, a cold‐water adapted lineage thought to be particularly vulnerable to climate warming (Solokas et al. 2023). As a result, mechanisms responsible for climate‐related decreases in body size are under debate and may include metabolic controls, trophic relationships, increased competition, and growth‐reproduction trade‐offs (Holt and Jørgensen 2015; Morais and Bellwood 2018; van der Have and de Jong 1996; Verberk et al. 2021; Wootton et al. 2022).

Life stage, thermal guild, population density, habitat characteristics, and/or using different measures of climate or growth may explain variable growth responses to climate warming within and among fish species (Audzijonyte et al. 2025; Grabda et al. 2025; Warne et al. 2024). Disentangling these factors from climate effects is difficult because of a lack of long‐term empirical data from different species and life stages. In experimental laboratory settings, decreases in adult body size in response to warming are common (Atkinson 1994; Forster et al. 2012). However, recent studies using empirical data from marine and freshwater fishes have shown departures from the TSR (Audzijonyte et al. 2020; Grabda et al. 2025; Huss et al. 2019; Solokas et al. 2023; Warne et al. 2024). Divergent growth responses through time have also been found between younger and older Bluegill ( *Lepomis macrochirus* ), with younger fish shrinking, while older fish increased or had no change in total length from 1945 to 2019 (Grabda et al. 2025). Moreover, the same species may have different responses to climate warming between studies depending on which climate variable was used in analyses. For example, Lake Whitefish ( *Coregonus clupeaformis* ) were found to increase in body size at higher mean annual surface water temperatures by Solokas et al. (2023), while Warne et al. (2024) showed that Lake Whitefish decreased in body size with increasing growing degree days. Additional long‐term studies that examine multiple variables representing climate warming and incorporate age‐specific information are needed to disentangle the effects of climate warming from other factors related to growth (Audzijonyte et al. 2025).

In this study, we used long‐term, regional‐scale data to disentangle effects of anthropogenic climate change from other factors that influence growth across age classes in a freshwater fish community. We used recently digitized historical survey data (Alofs et al. 2025; King et al. 2025) in tandem with contemporary survey data to examine 75 years (1945–2020) of data from 1497 inland lakes across Michigan to determine if there were changes in length‐at‐age for 13 species over time and as a function of climate warming and other factors. We used Bayesian hierarchical models to test for changes in length‐at‐age over time and boosted regression trees (BRTs) to disentangle effects of climate from other factors. We hypothesized that cold water adapted species would decrease in length‐at‐age with climate warming following the TSR, while warm water adapted species would increase in length‐at‐age as higher temperatures elevate metabolic rates but do not exceed thermal optima, and that cool water adapted species would have an intermediate or more variable response depending on lake water temperatures (Figure 1).

## 2 Materials and Methods

### 2.1 Data Description

**FIGURE 2:**
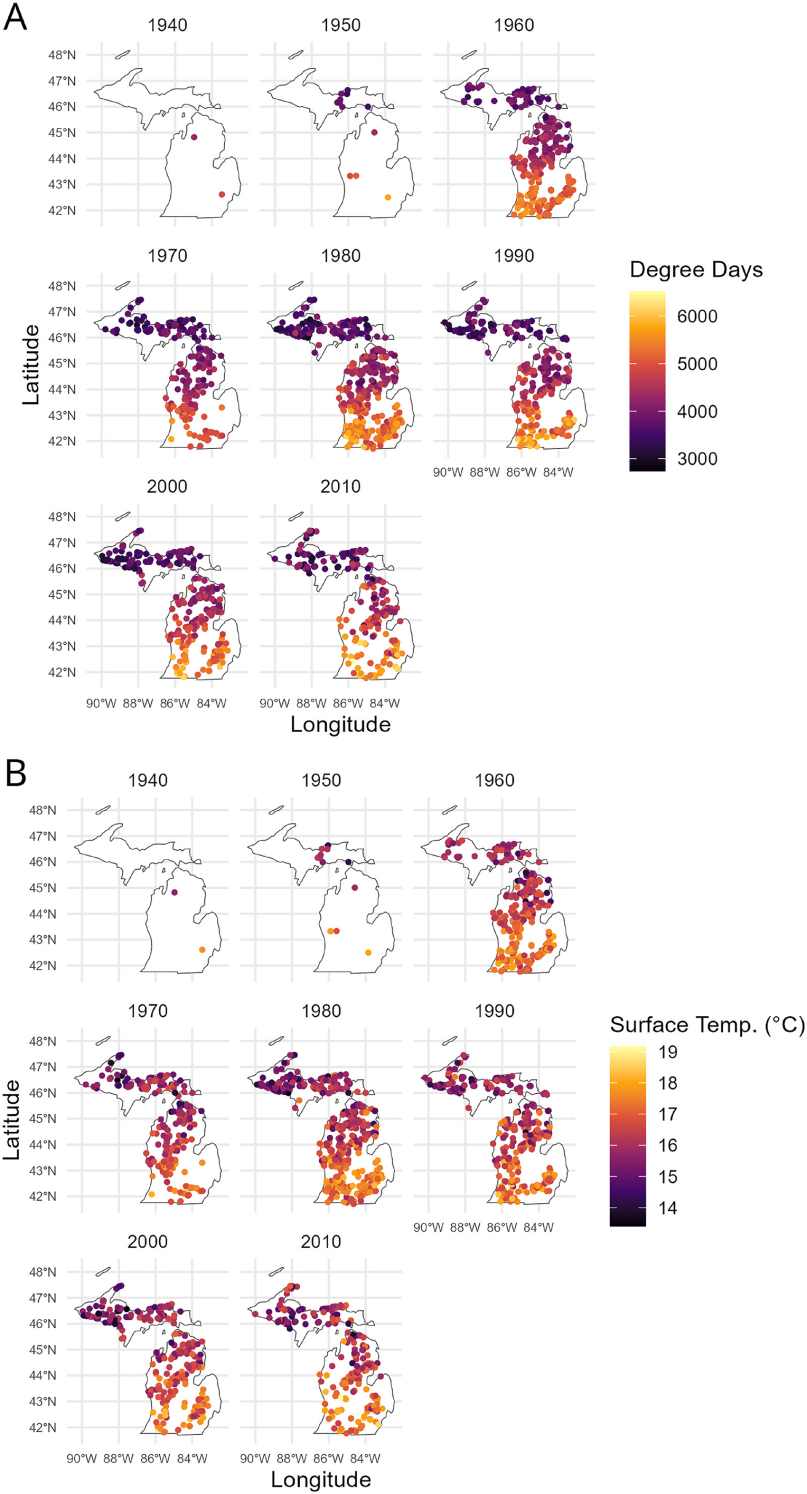
Map of lakes from across the state of Michigan where each dot represents an individual lake (*n* = 1497) showing the decadal average of (A) annual growing degree days and (B) mean annual surface water temperature for each lake sampled in that decade.

A collection of historical survey data from inland lakes in Michigan that includes information on fish growth and lake habitat is housed by the Michigan Department of Natural Resources (MDNR) Institute for Fisheries Research (IFR). This collection was recently transcribed after a community science effort through the online platform Zooniverse (Alofs et al. 2025; King et al. 2025). As a result, we were able to use these data to examine changes in fish growth over time using length‐at‐age (total length in mm) as a proxy for growth. Additionally, MDNR maintains a scale archive from which we transcribed and geolocated length‐at‐age data from envelopes of aged scales for fishes from inland lakes across the state of Michigan collected in the 1940s, '50s, and '60s. Historical data were combined with contemporary growth records from lake surveys conducted by MDNR including the Status and Trends Program (STP) from 2002 to 2020 (Wehrly et al. 2015). STP surveys sample fish populations in each lake with fyke and gill nets, seines, and boat electrofishing with standardized sampling effort (Hayes et al. 2003). Ages were based on scales and followed standard MDNR protocols until 2015. In 2015, MDNR updated their protocols to use structures other than scales (fin rays and spines) for certain species and size classes to increase agreement among agers (Table S1). We examined the data for corresponding differences in trends post‐2015 and did not find any obvious changes (Figure S1). The combined dataset included 36 species, 13 of which had enough data from different age classes to model through time in Bayesian models and across environmental variables in BRTs (Table 1). These species were from three different thermal guilds (as defined in Hasnain 2010): cold‐water adapted (Cisco, *Coregonus artedi* , Rainbow Trout, *Oncorhynchus mykiss* , and Brown Trout, *Salmo trutta* ), cool‐water adapted (Yellow Perch, *Perca flavescens* , Northern Pike, *Esox lucius* , Walleye, *Sander vitreus* , Black Crappie, *Pomoxis nigromaculatus* , White Sucker, *Catastomus commersonii*, and Rock Bass, *Ambloplites rupestris* ), and warm‐water adapted fishes (Smallmouth Bass, *Micropterus dolomieu* , Pumpkinseed Sunfish, *Lepomis gibbosus* , Largemouth Bass, *Micropterus nigricans*, and Bluegill). In total, the combined dataset included 84,452 observations of mean length‐at‐age from 1945 to 2020 across species, age classes, and surveys (29,893 contemporary, 54,559 historical). These data span all of Michigan and were from a total of 1497 inland lakes (Figure 2).

**TABLE 1:** Sample sizes per species age class.

Species	Thermal guild	Sample size per age class												
		0	1	2	3	4	5	6	7	8	9	10	11	12
Cisco	Cold	—	—	64	97	109	91	75	**55**	—	—	—	—	—
Rainbow Trout	Cold	—	158	168	123	59	—	—	—	—	—	—	—	—
Brown Trout	Cold	—	80	101	67	39	17	—	—	—	—	—	—	—
Yellow Perch	Cool	238	1165	2107	2495	2334	**1862**	**1337**	**822**	**476**	**261**	**153**	**64**	—
Northern Pike	Cool	196	1083	1705	1823	1568	**1249**	**911**	**559**	**307**	**151**	**72**	—	—
Walleye	Cool	205	503	680	772	707	647	**591**	**481**	**419**	**297**	**240**	**140**	**108**
Black Crappie	Cool	84	559	1136	1284	**1174**	**974**	**747**	**490**	**315**	**181**	**114**	**61**	—
White Sucker	Cool	—	56	94	113	103	**81**	—	—	—	—	—	—	—
Rock Bass	Cool	—	193	616	903	962	889	**772**	**631**	**477**	**320**	**198**	**105**	—
Smallmouth Bass	Warm	99	485	746	815	732	601	**500**	**359**	**265**	**176**	**112**	—	—
Pumpkinseed Sunfish	Warm	—	461	1205	1710	**1709**	**1422**	**1021**	**624**	**328**	**150**	**61**	—	—
Largemouth Bass	Warm	291	1186	1856	2125	1845	1553	**1182**	**844**	**613**	**419**	**250**	**136**	**84**
Bluegill	Warm	116	1108	1985	2466	2442	**2199**	**1777**	**1317**	**791**	**417**	**199**	**67**	—

*Note:* Species are sorted by increasing final temperature preferendum (FTP, Table S2). Adult age classes are in bold.

For each sampling event per lake per year, we compiled data on abiotic and biotic factors known to influence fish growth (Figures S2–S11). To facilitate communication of results, these factors were grouped into four categories: (1) lake attributes—lake area, maximum lake depth, and Secchi depth (a lake‐year specific measurement); (2) land cover—percent cover of adjacent urban, forest, wetland, and agriculture; (3) climate—growing degree days (base 0°C, hereafter “degree days”) and mean water surface temperature during the ice‐free period; and (4) sampling bias—day of year. For each lake, latitude, longitude, lake surface area, and lake depth were obtained from contemporary, georeferenced MDNR databases. Secchi depth (used as a proxy for primary production) was pulled from both historic and STP surveys when available, or mean Secchi depth of the summer stratified period (15 June–15 September) from LAGOS‐NE (Soranno et al. 2017, 2019). Land‐use land‐cover (LULC) data were assigned using watershed delineations from LAGOS‐NE combined with historic LULC data from a USGS model and contemporary data from the National Land Cover Database (Dewitz and US Geological Survey 2006; Sohl et al. 2016; Soranno et al. 2017). Lake mean annual surface water temperature was modeled using a modification of Shuter et al. (1983) described by King et al. (2023) and annual degree days were calculated as the sum of daily water temperatures above 0°C for the year. We averaged annual degree days over the life of each fish. For example, for an age‐4 fish, we averaged degree days over the 4 years prior to the year in which the fish was caught. To account for age‐specific changes in length, all statistical analyses were performed to obtain effects per explanatory variable per individual age class within a species. Examining age‐specific effects limits potential biases associated with varied sampling methods. While different methods capture individuals of different sizes (e.g., based on mesh size), it is unlikely that different methods would create a within‐age‐class bias in the length of fishes collected.

### 2.2 Changes in Length‐at‐Age Through Time

To quantify temporal changes in fish length‐at‐age, we fit a Bayesian hierarchical linear regression. Mean total length (mm) was modeled as a function of habitat characteristics to account for variation among lake types (lake area and maximum depth), day of year, and year within each species age class using the *brms* R package (Bürkner 2017):

$$\begin{array}{c} \text{Mean total length}\;\left(\mathrm{mm}\right)\sim \ln\left(\text{maximum depth}\right)+\ln\left(\text{area}\right)\\ \;+\mathrm{day}\;\text{of year}+\left(1+\text{year}\;\right|\;\text{spcies}\;\mathrm{age}\;\text{class}) \end{array}$$

This model formula was chosen because of well established relationships between lake depth and area and day of year on fish growth (e.g., Grabda et al. 2025) that we wanted to control for when examining temporal trends. The random effect permits each species age class to have its own slope and intercept as a function of year. The model was performed using four chains with 100,000 iterations and weakly informative (package default) priors.

Next, we used a Bayesian meta‐regression framework to investigate if the temporal trend (effect of year on total length from the hierarchical model) among species age classes varied by final temperature preferendum (FTP, temperature a species consistently gravitates towards in a temperature gradient; Hasnain 2010) and life stage (adult versus juvenile). Among the cold‐water adapted species, there was only one adult age class (age‐7 Cisco). This limited our ability to compare across life stages and thermal guilds, which is why we used FTP in this model as opposed to thermal guild. Posterior means and 95% credible intervals for the estimated year slope (i.e., annual rate of change in mean total length) for each species age group were extracted from the first hierarchical model. The corresponding posterior standard error was used as a measure of uncertainty in the meta‐regression:

$${\text{slope}}_{\text{group}}\;|\;{\mathrm{se}}_{\text{slope}}\sim \mathrm{FTP}*\text{life stage}$$

where slope_group_ is the estimated annual trend (effect of year on length in mm) for each species age class, and se_slope_ is the standard error of that slope estimate. This model was performed using four chains, 2000 iterations, and weakly informative priors. This approach propagates uncertainty in the slope estimates from the hierarchical model by assigning less weight to species age classes with greater uncertainty. This sequential modeling strategy allowed us to first estimate within‐group temporal trends while controlling for confounding variables, and then explicitly test whether adult versus juvenile differences in annual trends were associated with variation in FTP. By modeling the slope uncertainties in the second stage, we avoided overconfidence in inferences about predictors of temporal trends. Meta‐regression was performed on slopes, rather than slopes expressed as percent change, to facilitate propagation of error from the hierarchical model.

### 2.3 Boosted Regression Trees Disentangling Climate Effects From Other Factors on Growth

We used boosted regression trees (BRTs), a machine learning technique, to disentangle the effects of climate variables from other factors on mean length‐at‐age. Boosted regression trees were chosen for their ability to handle heterogeneous and patchy covariates, different types of predictor variables, and various statistical distributions (Elith et al. 2008). One BRT was generated per age class per species for a total of 114 BRT models. BRTs do not rely on *p*‐values. Predictors are statistically important in BRTs when their relative influence exceeds 1/*n*, where *n* is the number of predictors in the model. BRT hyperparameters in our model fitting were as follows: learning rate = 0.001, bag rate = 0.5, tree complexity = 5, bag fraction = 0.5, and step size = 100. BRT modeling was conducted via the *dismo* package in R (Hijamns et al. 2023). We report whether climate variables were having positive or negative effects on length‐at‐age for surface temperatures > 17°C and degree days > 5000. These cutoff values were based on the third quantile in our dataset for surface temperature (17.1°C) and degree days (5118) and rounded to values that could be clearly interpreted on plots to understand the effects of continued and future climate warming on fish growth.

## 3 Results

### 3.1 Changes in Length‐at‐Age Through Time

**FIGURE 3:**
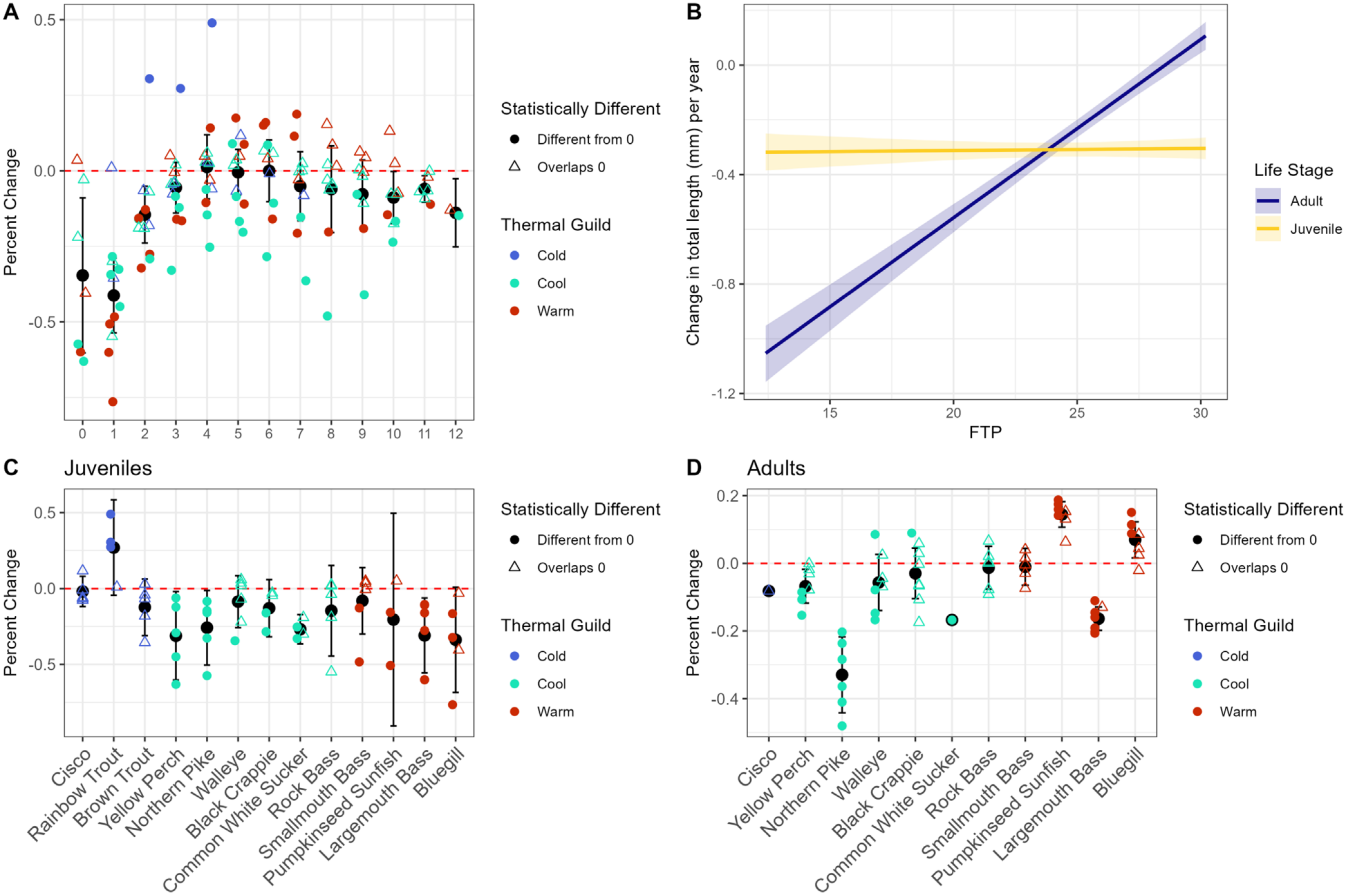
Partial effects of year on length‐at‐age (total length in mm) from Bayesian hierarchical linear regression with the partial effect expressed as annual percent change in mean length (A) across age classes and by species for (C) juveniles and (D) adults. The partial effect of FTP on change in length‐at‐age per year (B) was statistically positive for adults and not different from zero for juveniles. Black points represent group means and associated error bars represent a 95% confidence interval for that group.

Bayesian hierarchical linear regression revealed that 46% (58/125) of species age classes had statistically important changes (i.e., the 95% credibility interval did not overlap zero) in length through time with 37% (46/125) statistically decreasing. For cold‐water adapted species, Cisco and Brown Trout had no statistical changes in length through time, while Rainbow Trout ages 2–4 statistically increased in length through time (0.27%–0.49% per year or 1.0–2.47 mm/year, Figure 3, Figures S12 and S13, Table S2). Among the cool‐water adapted species, statistical changes through time were documented in 45% (29/64) of cool‐water adapted age classes and all but two of those were decreases. Yellow Perch ages 0–3 and 5–7 statistically decreased in length (−0.09% to −0.63% per year or −0.19 to −0.50 mm/year). All Northern Pike age classes in this study (0–10) statistically decreased (−0.08% to −0.57% per year or −0.46 to −3.89 mm/year). Walleye ages 1, 9, 10, and 12 statistically decreased (−0.08% to −0.34% per year or −0.45 to −0.98 mm/year), while age 6 statistically increased in length through time (0.09% per year or 0.43 mm/year). Black Crappie ages 1 and 2 statistically decreased in length through time (−0.28% per year or −0.36 mm/year and −0.16% or −0.27 mm/year, respectively) while age 5 statistically increased (0.09% per year or 0.22 mm/year). Common White Sucker ages 3–5 statistically decreased in length through time (−0.17% to −0.33% per year or −0.75 to −1.21 mm/year). Rock Bass had neither statistical increases nor decreases in length through time. For warm‐water adapted fishes, statistical changes in length through time (57% or 26/46 age classes) were usually decreases (41% or 19/46 age classes). Smallmouth Bass ages 1 and 2 statistically decreased in length through time (−0.48% per year or −0.68 mm/year and −0.13% or −0.27 mm/year, respectively). Pumpkinseed Sunfish ages 1 and 2 statistically decreased in length through time (−0.51% per year or −0.39 mm/year and −0.16% or −0.17 mm/year, respectively), while ages 4–7 statistically increased (0.14%–0.19% per year or 0.21–0.36 mm/year). Largemouth Bass ages 0–11 (all ages in this study besides age 12) statistically decreased in length through time (−0.11% to −0.60% or −0.32 to −0.86 mm/year).

The largest decreases in length through time were observed in the youngest and oldest age classes. On average across species, the largest decreases were observed in age‐12 (−0.77 mm/year ± 0.19, mean ± one standard deviation), age‐1 (−0.59 ± 0.31 mm/year), age‐9 (−0.56 ± 1.2 mm/year), age‐10 (−0.53 ± 0.70 mm/year), age‐8 (−0.52 ± 1.3 mm/year), and age‐0 (−0.45 ± 0.51 mm/year, Table S3). Across species, the largest decreases as a percent of mean length‐at‐age were age‐1 (−0.41% ± 0.20% per year), age‐0 (−0.35% ± 0.28% per year), age‐2 (−0.15% ± 0.16% per year), age‐12 (−0.14% ± 0.01% per year), and age‐10 (−0.09% ± 0.11% per year, Table S4). Across species, juveniles were more likely than adults to decrease in length‐at‐age with exceptions being cold‐water adapted Rainbow Trout ages 2–4 (Figure 3C, Table S2). For adults among species, statistical increases through time were common for some warm‐water adapted species like Bluegill and Pumpkinseed Sunfish, while only two adult age classes of cool‐water adapted fishes statistically increased through time (Black Crappie age‐5 and Walleye age‐6).

The effect size of year (time) on length‐at‐age as a function of final temperature preferendum (FTP) differed for juvenile and adult fishes. In the Bayesian meta‐regression, the interaction between FTP and life stage was statistically important (−0.06, −0.07 to −0.05; estimate, 95% credibility interval) (Figure 3B, Table S5). Post hoc comparison of these FTP trends to zero per life stage revealed that adults increased through time relative to FTP (0.065, 0.06–0.07), while there was no change in juveniles (0.001, −0.01 to 0.01, Table S5B). Similarly, post hoc pairwise comparison revealed that adults increased statistically more than juveniles (0.064, 0.06–0.07, Table S5C). Differences in trends between juveniles and adults were influenced by decreases in adult Northern Pike and increases in juvenile Rainbow Trout, which lack data for adults. After removing these species from the analysis, the partial effect of FTP for adults was weakly positive with a much broader 95% credibility interval and no longer statistically different from zero (Figure S14).

### 3.2 Boosted Regression Trees Disentangling Climate Effects From Other Factors on Growth

**FIGURE 4:**
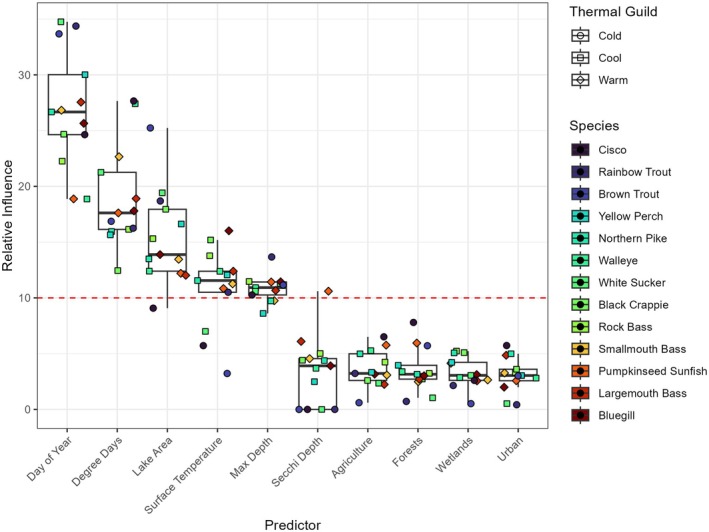
Mean relative influence per species and predictor variable included in BRTs. Relative influence below ten means that predictor was not statistically important. See Appendix S1 for relative influence plots for each individual species age group (Figures S15–S37). Boxes represent the first to third quartiles, the horizontal black line is the median, and whiskers extend to the minimum and maximum values within 1.5 times the interquartile range minus the first or plus the third quartile on the bottom and top, respectively.

**FIGURE 5:**
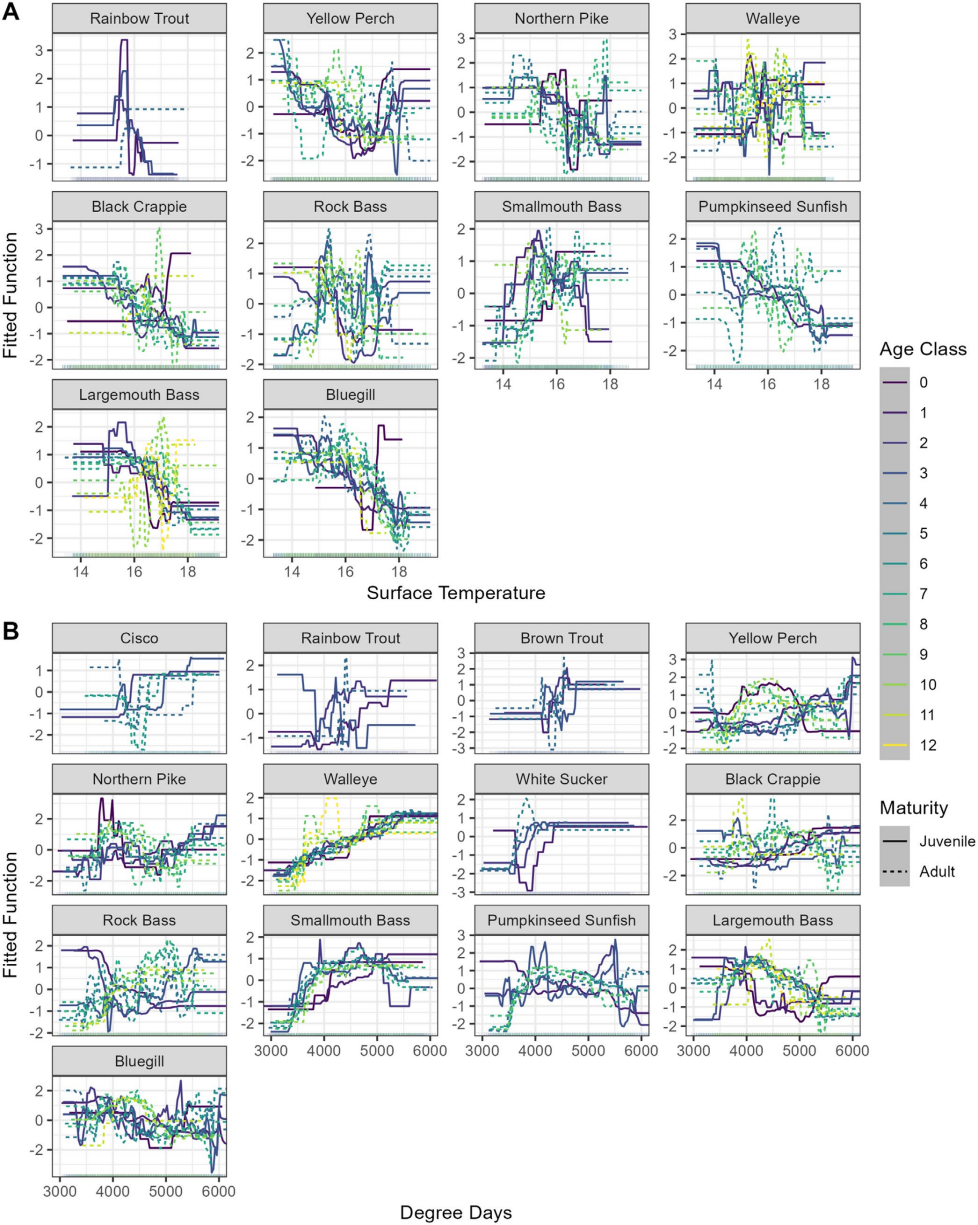
Partial dependency plots from BRTs for (A) effect of mean annual water surface temperature on length‐at‐age and (B) effect of lifetime growing degree days on length‐at‐age. Predictors have a positive effect when the fitted function is above zero, and a negative effect below zero. Species panels were arranged from top left to bottom right by descending final temperature preferendum. Rug plots are included in all panels to show the distribution of the response across that predictor (see Figures S2 and S3 for distributions of each species per age class across mean annual surface temperature and mean annual lifetime growing degree days, respectively). If the effect of either mean annual water surface temperature or mean lifetime growing degree days were below the relative influence threshold for statistical importance (10%, Figure 4, Figures S15–S37) for a given species age group, those species age groups were excluded from the plot. See Appendix S1 for partial dependency plots for all environmental predictors for each individual species age class (Figures S16–S38).

**FIGURE 6:**
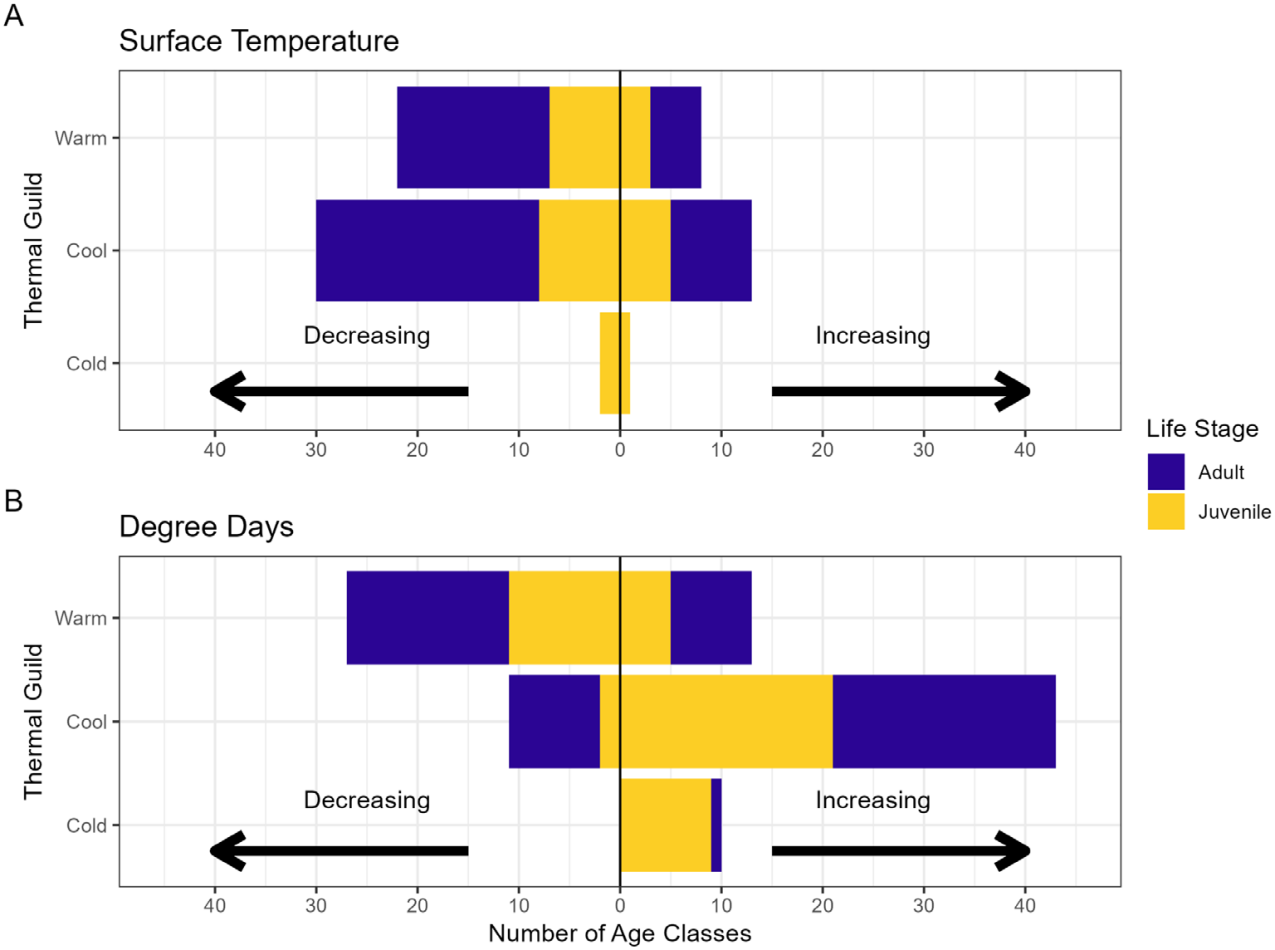
Synthesis for direction of effects of (A) surface temperature and (B) degree days on length‐at‐age from BRTs. Species age classes increasing in total length at > 5000 degree days or > 17°C are to the right of zero (vertical line), while those decreasing at those values are to the left of zero. Only species age classes for which surface temperature or degree days, respectively, had a relative influence greater than ten were included.

BRTs revealed that, across species and age classes, day of year, lake area, max depth, degree days, and surface temperature had the most influence on mean length‐at‐age, and land cover variables were rarely important. On average, across species and age classes, BRTs explained 43% of variance in total length data (Table S6). Day of year was the most important predictor of length‐at‐age for 77% of species (10/13) and had the highest mean relative influence among species (Figure 4). The exceptions were Smallmouth Bass, Largemouth Bass, and Walleye. For these species, degree days were the most important predictor across age classes. At higher values of degree days (> 5000) and surface water temperature (> 17°C), there were diverging effects within thermal guilds, except for warm‐water adapted fishes (Figure 5, Figures S15–S38). Most cold and cool‐water adapted fishes increased in length‐at‐age at degree days > 5000; however, when mean annual surface water temperature exceeded 17°C, half of cold‐water adapted fish age classes decreased in length, while most cool‐water adapted age classes decreased in length (Figure 6). Meanwhile, most age classes of warm‐water adapted species increased with both increasing degree days and surface temperature. The direction of response to higher values of surface temperature and degree days, respectively was generally the same for juveniles and adults within a thermal guild (Figure 6). Surface temperature was not statistically important for any age classes of cold‐water adapted Brown Trout. The direction of change in length‐at‐age through time was more likely to correspond to the direction of the response of length‐at‐age to surface temperature (8/13 species) than to degree days.

## 4 Discussion

Most fish species' age classes in this study were shrinking both through time and at higher water temperatures. There have been relatively few studies on the effects of climate warming on body size in freshwater fish communities, and those studies have found mixed directions of responses to climate warming (Jeppesen et al. 2012; Rypel et al. 2016; Solokas et al. 2023; Warne et al. 2024). For cold‐ and cool‐water adapted fishes in this study, we found that, on average, the effect of climate depended on the variable examined with more degree days typically leading to increases in body size, while elevated mean annual surface water temperatures usually led to decreases. Moreover, there were multiple species where juveniles and adults had divergent length‐at‐age responses through time (e.g., Bluegill) or to climate variables (e.g., Yellow Perch). Mixed results in the literature may be the result of studies choosing different variables to represent climate warming (e.g., surface water temperature vs. degree days) and/or not taking population age structure into account. Accounting for the effect of different climate variables (representing various dimensions of climate change) may explain variation in size changes across temporal and spatial scales.

### 4.1 Divergent Trends Between Growing Degree Days and Water Surface Temperature

Boosted regression trees revealed that, for cold and cool‐water adapted fishes, length‐at‐age responses to increasing degree days and mean annual surface water temperature were usually in opposite directions, which may explain some discrepancies among previous studies. Solokas et al. (2023) and Warne et al. (2024) both examined responses of Lake Whitefish and Lake Trout (*Salvelinus namayacush*) to climate warming, using average annual surface water temperature and growing degree days, respectively, and these two studies found divergent trends in body size for each species. These discrepancies, when coupled with our results, highlight that the variable chosen to represent climate warming can reverse the direction of the observed trend. Divergent responses to degree days and mean surface temperature may be related to changes in growing season duration versus thermal habitat suitability. Degree days are important indicators of climate impacts and growth (Alofs and Jackson 2015; Massie et al. 2021; Venturelli et al. 2010) yet may not necessarily correlate with responses to changes in mean water temperature. For temperate lakes, like those in this study, degree days are usually calculated using the ice‐free period of the year, which may underrepresent climate warming that is disproportionately increasing winter temperatures (Winslow et al. 2017). Further, changes in phenology represented by degree days differentially affect fishes of different thermal guilds (Shuter et al. 2012). For example, Xu et al. (2024) suggested that changes observed in thermal habitat were related to increased degree days via expansion of the growing season (earlier spring, later winter). They also found these changes led to faster losses of preferred water temperature days for cool‐water fishes than gains in preferred days for warm‐water fishes. However, increased surface water temperature could result in more days beyond thermal suitability thresholds and/or be more tightly correlated to losses in summertime thermal habitat. Future studies on the effects of climate warming on body size should incorporate multiple metrics of climate warming (e.g., representing changes in both temperature and phenology that may influence growth through different mechanisms) that are biologically meaningful to the species and response being considered.

Discrepancies in the direction of length‐at‐age responses to degree days and surface temperature may indicate that changes in seasonality with climate change (e.g., McMeans et al. 2020) are an important driver of changes in length‐at‐age for fishes in this study. In addition to changes in growing season duration and thermal habitat suitability, temperature seasonality can shape intra‐ and inter‐specific variation in body size through environmental filtering and niche partitioning (Chia et al. 2024). For example, Alpine ibex ( *Capra ibex* ) increased in body mass due to a reduction in winter mass loss with milder winters (reduced environmental filter) that result from climate change (Brambilla et al. 2024). This example demonstrates that temperature gradients in growth can reverse seasonally. Moreover, in Chinook Salmon ( *Oncorhynchus tshawytscha* ), there is a trade‐off between more spring growth in downstream relative to upstream populations due to warmer temperatures, and relatively more summer growth in upstream populations due to higher upstream prey production in summer (Kaylor et al. 2021). This seasonal reversal in temperature effects on growth highlights the need to examine other factors (e.g., prey production) that interact with temperature (and growing season timing and duration) to indirectly impact growth as climate change continues.

### 4.2 Largest Decreases in Length in Youngest and Oldest Fishes

Over the last 75 years, across species in this study, the highest mean annual decrease in length‐at‐age as a percentage of mean length was usually in the youngest fishes (ages 0 and 1). This result is the opposite of what is predicted by the TSR (Atkinson 1994), indicating factors besides temperature may play a role in observed juvenile decreases in length‐at‐age. This may be further indicated by the lack of a statistically important relationship between change in length for juveniles and species FTP. Fish predation is generally gape limited, and as a result, decreasing body size increases predation risk (Persson et al. 1996). Further, increased predation risk decreases foraging and slows prey growth (Urban 2007). Decreasing body size coupled with this growth‐predation risk trade‐off may create a negative feedback loop for fish body size, further exacerbating climate‐related decreases in body size. For species in this study where adults increased or had no change in total length over time, while juveniles shrank (e.g., most centrarchids), shrinkage may be related to density‐dependent growth (Osenberg et al. 1988). Warmer temperatures lead to fishes reaching sexual maturity at smaller sizes and increased energy allocation to reproduction (Thunell et al. 2023; White et al. 2020), which is likely to increase the density of juvenile cohorts. For species where growth is strongly density‐dependent, temperature‐driven increases in recruitment, or decreases in food availability (related to productivity or competition), would indirectly decrease growth (Grabda et al. 2025). For some species, decreases in juvenile growth were followed by what appears to be compensatory growth in intermediate age classes, and in some cases increases in growth in older age classes (e.g., Bluegill). For these species, ontogenetic shifts in diet and habitat use that occur in this intermediate age range may reduce food limitation and/or intraspecific competition reducing density‐dependent effects on growth (Grabda et al. 2025; Osenberg et al. 1988).

In several species (Black Crappie, Rock Bass, Walleye, and Northern Pike), after the youngest age classes, the oldest age classes had the next highest mean annual decreases in length‐at‐age. Across taxa, older individuals disproportionately contribute to cultural transmission, population dynamics, and ecosystem processes and services (Kopf et al. 2025). For instance, fish reproductive output exponentially increases with body size (Barneche et al. 2018). Decreases in length in the oldest and largest individuals can disproportionately reduce fecundity and thereby population productivity and stability (Hixon et al. 2014). Moreover, fishing pressure is an additional stressor that disproportionately impacts older, larger individuals (Ayllón et al. 2018). Notably, fishing has decreased the proportion of old fishes in 79%–97% of populations across five oceans (Barnett et al. 2017). Similar declines have been documented for the largest and oldest freshwater fishes (He et al. 2019; Rypel et al. 2021). Like the youngest age classes, the oldest age classes have specific conservation challenges that may negatively interact with climate change to influence population dynamics. Management and conservation approaches need to consider species‐specific age‐ and size‐based strategies over decades to restore older individuals to populations (Kopf et al. 2025).

### 4.3 Differences Among Thermal Guilds in Length‐at‐Age Responses to Climate Change

We found cold‐water adapted salmonids increased in length‐at‐age as a function of degree days, while mean surface temperature was rarely statistically important in our models for cold‐water adapted species, and when it was important responses were split in direction. Both Solokas et al. (2023) and Al‐Chokhachy et al. (2022) found a positive relationship between water temperature and growth in lakes and streams, respectively. In contrast, Warne et al. (2024) found salmonids (Lake Whitefish and Lake Trout) decreased or had no change in body size with increasing degree days. Cold‐water species increasing in length‐at‐age as a function of degree days may be the result of climate‐induced shifts in phenology that cause temporal partitioning of food resources that disproportionately benefit cold‐water fishes, particularly winter spawners (Shuter et al. 2012). Railsback (2022) posits that for salmonids: food consumption is at least as important as temperature for growth, there is no optimal temperature for growth under natural food consumption rates, and that effects of temperature on growth can be stronger during cooler seasons. Increasing degree days means a longer growing season, which could explain larger body size as a function of increasing degree days, particularly when there are beneficial phenological shifts in food availability. Conversely, decreases in length‐at‐age with increasing degree days may indicate food limitation or phenological shifts in food availability that do not benefit cold‐water adapted fishes.

Additionally, changes in salmonid length‐at‐age over time may be related to stocking and/or harvesting and their potential interactions with climate change. For example, over the past 75 years scientists have learned more about environmental conditions conducive to salmonid growth. In Michigan, salmonid stocking has shifted over time to focus more on lakes with optimal conditions for growth. This change in which lakes are stocked may contribute to observed increases in length‐at‐age through time for Rainbow Trout in this study. Conversely, in some situations, harvesting can reduce length‐at‐age over time. For instance, a size‐selective fishery for Alaskan sockeye salmon ( *Oncorhynchus nerka* ) led to decreased length‐at‐age over time and decreased size at maturity (Kendall et al. 2014). Effects of stocking and harvest are likely to interact with climate‐driven changes in length and should be accounted for when data are available to do so.

Surprisingly, warm‐water adapted fishes were the only thermal guild where most age classes were decreasing in length‐at‐age with both increasing surface temperature and increasing degree days. It is unlikely climate warming is directly decreasing length‐at‐age over time for warm‐water adapted species (Hoxmeier et al. 2009). For instance, climate‐based bioenergetics models of bluegill growth predicted increasing growth at higher degree days; however, evidence of decreasing juvenile length‐at‐age through time may indicate food limitation with increased metabolic demand, possibly through increased intraspecific competition (Grabda et al. 2025). Similarly, observed decreases in length‐at‐age through time of Largemouth Bass may be related to decreases in length‐at‐age we documented in their prey, which includes Yellow Perch and juvenile sunfishes (Clady 1974; Otis et al. 1998). Largemouth Bass growth is also subject to negative density‐dependent feedback and changes in angling behavior (Hansen et al. 2015; Sass and Shaw 2020). In Wisconsin lakes, Largemouth Bass abundance has increased from 1944 to 2012, attributed to catch‐and‐release angling, more growing degree days, and increased water temperatures (Hansen et al. 2017; Rypel et al. 2016). As a result, Largemouth Bass size structure and growth have decreased over time (Sass and Shaw 2020). In addition to the effects of climate change documented in this study, similar factors (negative density dependence and changes in angling behavior) likely also contributed to observed changes in length‐at‐age for warm‐water adapted fishes.

### 4.4 Caveats and Limitations

The heterogeneous nature of this dataset presents some inherent caveats and limitations to these analyses. As noted in the methods, through time different sampling methods were used to collect species included in this dataset. Those sampling methods capture different size ranges of fishes, which would introduce bias in length. However, because we modeled length‐at‐age, we were able to limit the impact of such bias. Different methods may be better geared towards capturing relatively larger (older) or smaller (younger) fishes, but there is no reason to expect that for a given age class different sampling methods would introduce a length‐at‐age bias. For example, different sampling methods should not result in collecting age‐4 fishes of inherently different size ranges. Next, adult age classes for cold‐water adapted fishes except for age‐7 Cisco did not have enough data to generate boosted regression trees and were therefore excluded from analyses. This was also true for White Sucker where age‐5 was the only adult age class with enough data for modeling. Similarly, age‐0 fishes lacked enough data to model for Cisco, Rainbow Trout, Brown Trout, White Sucker, Rock Bass, and Pumpkinseed Sunfish. Small sample sizes for adult age classes limited our ability to detect changes in how different life stages were changing in length‐at‐age through time with respect to FTP. While we were able to detect a clear statistical difference, more adult age classes across a wider FTP range would lend additional support. Further, based on age‐0 having some of the largest annual decreases in length, we may have missed ecologically important changes in length for species that lacked enough age‐0 data to model. Additionally, we were only able to account for some causes of variation in growth among lakes (using lake area and depth and Secchi depth for example). Lakes also vary in nutrient status, mixing, and primary productivity, among other factors that can influence fish growth, but we did not have sufficient measures of these factors across lakes and through time to include them in our analyses. Moreover, no individual lake had enough of a time series to use individual lakes as a random effect to control among lake variation in the influence of those factors on growth. Nonetheless, our Bayesian hierarchical and meta‐regression models and boosted regression trees represent ecologically meaningful trends which were attributed to plausible mechanisms by considering many species, age classes, and lakes (*n* = 1497) over 75 years.

## 5 Conclusions

Overall, in this study, most age classes of fishes were decreasing in length‐at‐age both through time and as a function of increasing mean surface water temperatures. In contrast to predictions from the TSR, the youngest age classes decreased in length the most. Additional factors beyond temperature, particularly food consumption, are important and may interact with climate change to influence fish growth (Massie et al. 2021). Future studies should examine if long‐term changes in trophic dynamics underpin species changes in body size. One potential avenue for this would be to leverage stable isotope samples from museum collections (Turner et al. 2023). Further, across a diverse set of lake fishes, the largest decreases in length‐at‐age were observed in the youngest and oldest fishes. Greater decreases in body size for the youngest and oldest fishes could lead to destabilized population dynamics, trophic cascades, biodiversity loss, and diminished ecosystem functions and services. Recognizing age‐ and size‐specific conservation challenges can point to management strategies that protect young and old individuals and can outperform traditional management approaches (Ahrens et al. 2020; Kopf et al. 2025; Marshall et al. 2021). Based on our results, mixed directions of growth responses to climate change for fishes in the literature may be related to not considering age‐specific changes in growth and/or different climate variables used in modeling. When possible, we recommend using multiple climate variables to capture different aspects of warming and different mechanisms through which warming may influence growth. When these factors (differences across age classes and climate variables) are accounted for across studies, fish body size responses to climate change may be more predictable than previously thought.

## Author Contributions

**Peter J. Flood:** conceptualization, data curation, formal analysis, investigation, methodology, software, visualization, writing – original draft. **Kaitlin E. Schiller:** investigation, methodology, writing – review and editing. **Katelyn B. S. King:** data curation, software, writing – review and editing. **Andrew D. Runyon:** data curation, investigation, writing – review and editing. **Kevin E. Wehrly:** data curation, project administration, resources, writing – review and editing. **Karen M. Alofs:** conceptualization, funding acquisition, methodology, project administration, resources, supervision, writing – review and editing.

## Conflicts of Interest

The authors declare no conflicts of interest.

## Supporting information


**Appendix S1:** gcb70584‐sup‐0001‐AppendixS1.pdf.

## Data Availability

Data and R code for this manuscript are available at https://doi.org/10.5281/zenodo.17288790.
