# Supplementary material for: Long‐Term and Regional‐Scale Data Reveal Divergent Trends of Different Climate Variables on Fish Body Size Over 75 Years
Source: Glob Chang Biol. 2025 Nov 5;31(11):e70584. doi: 10.1111/gcb.70584 (PMC12587105; doi:10.1111/gcb.70584)

Appendix S1 to accompany:

Global Change Biology

Long-term and regional-scale data reveal divergent trends of different climate variables on fish body size over 75 years

Peter J. Flood[1†], Kaitlin Schiller[1], Katelyn B. S. King[1, 2], Andrew Runyon[1], Kevin E. Wehrly[2], Karen M. Alofs[1]

[1]University of Michigan, School for Environment and Sustainability, Ann Arbor MI
[2]Michigan Department of Natural Resources, Institute for Fisheries Research, Ann Arbor MI

[†]Corresponding author full address: Dana Building, 440 Church St, Ann Arbor, MI, 48109

Corresponding author email: pjflood@umich.edu

Table of Contents

Supplementary Tables 6

Table S1. Summary table of aging methods per species based on total length in inches. Species not included in this table were aged based on scales. 6

Table S2. Slopes (change in length per year in mm) from the Bayesian hierarchical model for species age class and the associated standard error (SE) and lower and upper bounds of 95% credibility intervals (CI) for each slope estimate. Slopes where the 95% CI does not overlap zero were statistically important (functionally like a significant difference) and are denoted with an asterisk by the species name in that row. Mean length is the mean total length in mm for the species age class from this data set. Final temperature preferendum (FTP) and critical thermal maximum (CT_{max}) are listed for each species. 7

Table S3. Effect of year on total length in mm per species age class from Bayesian hierarchical models summarized per species life stage (juvenile and adult) as the mean and standard deviation (SD) for that species life stage expressed as the slope (annual change in length in mm) and annual percent change relative to the mean total length for a given species age class. 10

Table S4. Effect of year on total length in mm per species age class from Bayesian hierarchical models summarized per age class summarized as the mean and standard deviation (SD) expressed as both the slope (annual change in length in mm) and that slope expressed as percent change relative to mean total length of a given species age class. 11

Table S5. A) Model results from Bayesian meta-regression that examined if the effect of year on total length from the Bayesian hierarchical model varied among species age classes with respect to final temperature preferendum (FTP). B) Post-hoc test comparing adult and juvenile effects from Bayesian meta-regression to zero. For adults the relationship with FTP was positive and statistically different from zero. The juvenile relationship with FTP did not differ from zero as indicated by the 95% credibility interval overlapping with zero. C) Post-hoc pairwise comparison of the effects of adult and juvenile relationships with FTP. These effects were statistically different from one another as indicated by the credibility interval not overlapping zero. 12

Table S6. Model performance and summary metrics for all boosted regression tree models. 13

Supplementary Figures 16

Figure S1. Loess fits for each species age class through time to examine if there were any obvious shifts in length trends through time after 2015 when Michigan DNR aging protocols started to incorporate other aging structures in addition to scales for larger fishes (Table S1). The vertical red dashed line is placed at 2015. We were unable to discern any clear, consistent patterns that may be from this change in aging protocol. 16

Figure S2. Distribution of mean annual lake surface water temperature across species and age classes. 17

Figure S3. Distribution of mean lifetime growing degree days across species and age classes. 18
Figure S4. Distribution of day of year across species and age classes. 19
Figure S5. Distribution of log_{10}-transformed lake areas across species and age classes. 20
Figure S6. Distribution of log_{10}-transformed lake maximum depth across species and age classes. 21
Figure S7. Distribution of Secchi depth across species and age classes. 22
Figure S8. Distribution of percent wetland land cover across species and age classes. 23
Figure S9. Distribution of percent urban land cover across species and age classes. 24
Figure S10. Distribution of percent agricultural land cover across species and age classes. ... 25
Figure S11. Distribution of percent forest land cover across species and age classes. 26
Figure S12. Partial effects of year on length-at-age (total length in mm) from the Bayesian hierarchical model with the partial effect expressed as mean annual percent change in length-at-age. Species are arranged along the x-axis by ascending final temperature preferendum. Statistical differences were based on whether the 95% credibility interval did or did not overlap with zero (as indicated by point shape). 27
Figure S13. Partial effects of year on length-at-age (total length in mm) from Bayesian hierarchical modeling with the partial effect expressed as mean annual percent change in length-at-age. Species panels are ordered by ascending final temperature preferendum starting on the top left. Statistical differences were based on whether the 95% credibility interval did or did not overlap with zero (as indicated by point shape). 28
Figure S14. The relationship between the partial effect of year on total length from the Bayesian hierarchical model expressed as percent change relative to mean total length for a given species age class with Northern Pike and Rainbow Trout removed. 29
Figure S15. Relative influence of predictors from boosted regression trees for Cisco per age class. 30
Figure S16. Partial dependency plots for predictors from boosted regression trees for Cisco per age class. 31
Figure S17. Relative influence of predictors from boosted regression trees for Rainbow Trout per age class. 32
Figure S18. Partial dependency plots for predictors from boosted regression trees for Rainbow Trout per age class. 33
Figure S19. Relative influence of predictors from boosted regression trees for Brown Trout per age class. 34
Figure S20. Partial dependency plots for predictors from boosted regression trees for Brown Trout per age class. 35

Figure S21. Relative influence of predictors from boosted regression trees for Yellow Perch per age class. 36
Figure S22. Partial dependency plots for predictors from boosted regression trees for Yellow Perch per age class. 37
Figure S23. Relative influence of predictors from boosted regression trees for Northern Pike per age class. 38
Figure S24. Partial dependency plots for predictors from boosted regression trees for Northern Pike per age class. 39
Figure S25. Relative influence of predictors from boosted regression trees for White Sucker per age class. 40
Figure S26. Partial dependency plots for predictors from boosted regression trees for White Sucker per age class. 41
Figure S27. Relative influence of predictors from boosted regression trees for Black Crappie per age class. 42
Figure S28. Partial dependency plots for predictors from boosted regression trees for Black Crappie per age class. 43
Figure S29. Relative influence of predictors from boosted regression trees for Rock Bass per age class. 44
Figure S30. Partial dependency plots for predictors from boosted regression trees for Rock Bass per age class. 45
Figure S31. Relative influence of predictors from boosted regression trees for Smallmouth Bass per age class. 46
Figure S32. Partial dependency plots for predictors from boosted regression trees for Smallmouth Bass per age class. 47
Figure S33. Relative influence of predictors from boosted regression trees for Pumpkinseed Sunfish per age class. 48
Figure S34. Partial dependency plots for predictors from boosted regression trees for Pumpkinseed Sunfish per age class. 49
Figure S35. Relative influence of predictors from boosted regression trees for Largemouth Bass per age class. 50
Figure S36. Partial dependency plots for predictors from boosted regression trees for Largemouth Bass per age class. 51
Figure S37. Relative influence of predictors from boosted regression trees for Bluegill per age class. 52
Figure S38. Partial dependency plots for predictors from boosted regression trees for Bluegill per age class. 53

Figure S39. Trends in our data for A) annual mean lake surface temperature, B) annual growing degree days, C) mean annual growing degree days during the lifetime of each age class, and D) latitude. Trendline is a GAM fit with 0.95 confidence interval in shaded gray.. 54

Supplementary Tables

Table S1. Summary table of aging methods per species based on total length in inches. Species not included in this table were aged based on scales.

Species	Aging Method
Northern Pike	Scales 0 - 15.9 inches, dorsal fin ray (spine) ≥ 16 inches.
Walleye	Scales 0 - 9.9 inches, dorsal fin ray (spine) ≥ 10 inches.
Yellow Perch	Scales 0 - 5.9 inches, dorsal fin ray (spine) ≥ 6 inches.
Largemouth Bass	Scales 0 - 9.9 inches, dorsal fin ray (spine) ≥ 10 inches.
Smallmouth Bass	Scales 0 - 9.9 inches, dorsal fin ray (spine) ≥ 10 inches.
Bluegill	Scales 0 - 5.9 inches, dorsal fin ray (spine) ≥ 6 inches.
Pumpkinseed Sunfish	Scales 0 - 5.9 inches, dorsal fin ray (spine) ≥ 6 inches.
Black Crappie	Scales 0 - 5.9 inches, dorsal fin ray (spine) ≥ 6 inches.
Rainbow Trout	Length frequency < 4 inches, ≥ 4 inches scales
Brown Trout	Length frequency < 4 inches, 4-17 inches scales, ≥ 18 inches spine

Table S2. Slopes (change in length per year in mm) from the Bayesian hierarchical model for species age class and the associated standard error (SE) and lower and upper bounds of 95% credibility intervals (CI) for each slope estimate. Slopes where the 95% CI does not overlap zero were statistically important (functionally like a significant difference) and are denoted with an asterisk by the species name in that row. Mean length is the mean total length in mm for the species age class from this data set. Final temperature preferendum (FTP) and critical thermal maximum (CT_{max}) are listed for each species.

Species	Age	Change in Length per Year (mm)	SE	Lower 95% CI	Upper 95% CI	Thermal Guild	Life Stage	Mean Total Length	Percent Change in Length per Year	FTP	CTmax
Cisco	2	-0.16	0.28	-0.71	0.39	Cold	Juvenile	244.0	-0.07	12.4	26.9
Cisco	3	-0.21	0.24	-0.69	0.27	Cold	Juvenile	280.3	-0.08	12.4	26.9
Cisco	4	-0.19	0.21	-0.60	0.22	Cold	Juvenile	312.9	-0.06	12.4	26.9
Cisco	5	0.40	0.26	-0.11	0.89	Cold	Juvenile	341.8	0.12	12.4	26.9
Cisco	6	-0.03	0.24	-0.49	0.43	Cold	Juvenile	359.6	-0.01	12.4	26.9
Cisco	7	-0.31	0.34	-0.98	0.34	Cold	Adult	379.3	-0.08	12.4	26.9
Rainbow Trout	1	0.03	0.25	-0.46	0.51	Cold	Juvenile	259.6	0.01	15.5	22.1
Rainbow Trout*	2	1.00	0.22	0.58	1.43	Cold	Juvenile	329.3	0.30	15.5	22.1
Rainbow Trout*	3	1.10	0.25	0.62	1.59	Cold	Juvenile	405.0	0.27	15.5	22.1
Rainbow Trout*	4	2.47	0.35	1.79	3.17	Cold	Juvenile	505.2	0.49	15.5	22.1
Brown Trout	1	-0.78	0.42	-1.60	0.04	Cold	Juvenile	218.4	-0.35	15.7	28.3
Brown Trout	2	-0.57	0.30	-1.16	0.02	Cold	Juvenile	317.6	-0.18	15.7	28.3
Brown Trout	3	-0.17	0.28	-0.73	0.38	Cold	Juvenile	404.3	-0.04	15.7	28.3
Brown Trout	4	0.14	0.35	-0.55	0.82	Cold	Juvenile	483.5	0.03	15.7	28.3
Brown Trout	5	-0.37	0.41	-1.18	0.43	Cold	Juvenile	557.0	-0.07	15.7	28.3
Yellow Perch*	0	-0.50	0.19	-0.87	-0.13	Cool	Juvenile	79.1	-0.63	17.6	35
Yellow Perch*	1	-0.48	0.08	-0.64	-0.32	Cool	Juvenile	106.4	-0.45	17.6	35
Yellow Perch*	2	-0.42	0.06	-0.54	-0.30	Cool	Juvenile	144.7	-0.29	17.6	35
Yellow Perch*	3	-0.21	0.06	-0.32	-0.10	Cool	Juvenile	171.2	-0.12	17.6	35
Yellow Perch	4	-0.12	0.06	-0.24	0.00	Cool	Juvenile	196.8	-0.06	17.6	35
Yellow Perch*	5	-0.19	0.07	-0.32	-0.05	Cool	Adult	221.8	-0.09	17.6	35
Yellow Perch*	6	-0.26	0.08	-0.42	-0.10	Cool	Adult	243.8	-0.11	17.6	35
Yellow Perch*	7	-0.41	0.11	-0.62	-0.19	Cool	Adult	264.7	-0.15	17.6	35
Yellow Perch	8	-0.09	0.15	-0.37	0.20	Cool	Adult	284.3	-0.03	17.6	35
Yellow Perch	9	-0.05	0.20	-0.44	0.33	Cool	Adult	296.0	-0.02	17.6	35
Yellow Perch	10	-0.24	0.27	-0.78	0.30	Cool	Adult	309.8	-0.08	17.6	35
Yellow Perch	11	0.00	0.37	-0.72	0.72	Cool	Adult	321.4	0.00	17.6	35
Northern Pike*	0	-1.49	0.25	-1.97	-1.02	Cool	Juvenile	259.9	-0.57	20.7	32.6
Northern Pike*	1	-1.20	0.09	-1.38	-1.02	Cool	Juvenile	368.4	-0.33	20.7	32.6
Northern Pike*	2	-0.75	0.07	-0.89	-0.62	Cool	Juvenile	474.1	-0.16	20.7	32.6
Northern Pike*	3	-0.46	0.07	-0.60	-0.33	Cool	Juvenile	547.2	-0.08	20.7	32.6
Northern Pike*	4	-0.89	0.08	-1.03	-0.74	Cool	Juvenile	606.9	-0.15	20.7	32.6
Northern Pike*	5	-1.34	0.09	-1.51	-1.17	Cool	Adult	659.8	-0.20	20.7	32.6
Northern Pike*	6	-2.02	0.10	-2.21	-1.82	Cool	Adult	710.2	-0.28	20.7	32.6
Northern Pike*	7	-2.73	0.13	-2.97	-2.47	Cool	Adult	749.3	-0.36	20.7	32.6
Northern Pike*	8	-3.89	0.17	-4.22	-3.55	Cool	Adult	809.6	-0.48	20.7	32.6
Northern Pike*	9	-3.58	0.27	-4.10	-3.05	Cool	Adult	871.9	-0.41	20.7	32.6
Northern Pike*	10	-2.10	0.34	-2.76	-1.43	Cool	Adult	888.6	-0.24	20.7	32.6
Walleye	0	-0.39	0.37	-1.11	0.32	Cool	Juvenile	177.7	-0.22	22.5	23.4
Walleye*	1	-0.81	0.16	-1.11	-0.50	Cool	Juvenile	234.7	-0.34	22.5	23.4
Walleye	2	-0.22	0.14	-0.49	0.05	Cool	Juvenile	320.3	-0.07	22.5	23.4
Walleye	3	0.08	0.13	-0.18	0.33	Cool	Juvenile	380.4	0.02	22.5	23.4
Walleye	4	0.25	0.14	-0.01	0.52	Cool	Juvenile	430.2	0.06	22.5	23.4

Walleye	5	0.17	0.14	-0.09	0.44	Cool	Juvenile	471.2	0.04	22.5	23.4
Walleye*	6	0.43	0.14	0.15	0.70	Cool	Adult	502.5	0.09	22.5	23.4
Walleye	7	0.13	0.15	-0.15	0.42	Cool	Adult	531.1	0.02	22.5	23.4
Walleye	8	-0.25	0.16	-0.56	0.06	Cool	Adult	558.4	-0.04	22.5	23.4
Walleye*	9	-0.45	0.18	-0.79	-0.09	Cool	Adult	569.7	-0.08	22.5	23.4
Walleye*	10	-0.98	0.20	-1.38	-0.59	Cool	Adult	587.8	-0.17	22.5	23.4
Walleye	11	-0.42	0.26	-0.93	0.10	Cool	Adult	603.1	-0.07	22.5	23.4
Walleye*	12	-0.91	0.32	-1.53	-0.28	Cool	Adult	615.1	-0.15	22.5	23.4
Black Crappie	0	-0.03	0.32	-0.66	0.60	Cool	Juvenile	91.3	-0.03	23.4	34.9
Black Crappie*	1	-0.36	0.12	-0.60	-0.12	Cool	Juvenile	126.9	-0.28	23.4	34.9
Black Crappie*	2	-0.27	0.08	-0.43	-0.10	Cool	Juvenile	166.2	-0.16	23.4	34.9
Black Crappie	3	-0.09	0.08	-0.25	0.07	Cool	Juvenile	202.1	-0.05	23.4	34.9
Black Crappie	4	0.07	0.09	-0.11	0.24	Cool	Adult	228.7	0.03	23.4	34.9
Black Crappie*	5	0.22	0.10	0.02	0.43	Cool	Adult	248.0	0.09	23.4	34.9
Black Crappie	6	0.15	0.12	-0.07	0.38	Cool	Adult	266.0	0.06	23.4	34.9
Black Crappie	7	-0.01	0.15	-0.30	0.29	Cool	Adult	280.2	0.00	23.4	34.9
Black Crappie	8	-0.19	0.20	-0.57	0.20	Cool	Adult	293.1	-0.06	23.4	34.9
Black Crappie	9	-0.33	0.26	-0.83	0.18	Cool	Adult	305.6	-0.11	23.4	34.9
Black Crappie	10	-0.56	0.34	-1.23	0.12	Cool	Adult	318.3	-0.17	23.4	34.9
Black Crappie	11	-0.22	0.44	-1.09	0.64	Cool	Adult	327.7	-0.07	23.4	34.9
Common White Sucker	1	-0.57	0.39	-1.33	0.17	Cool	Juvenile	191.6	-0.30	23.4	31.6
Common White Sucker	2	-0.56	0.29	-1.13	0.01	Cool	Juvenile	293.9	-0.19	23.4	31.6
Common White Sucker*	3	-1.21	0.23	-1.67	-0.75	Cool	Juvenile	366.6	-0.33	23.4	31.6
Common White Sucker*	4	-1.07	0.25	-1.55	-0.59	Cool	Juvenile	423.6	-0.25	23.4	31.6
Common White Sucker*	5	-0.75	0.29	-1.31	-0.18	Cool	Adult	446.8	-0.17	23.4	31.6
Rock Bass	1	-0.41	0.24	-0.87	0.05	Cool	Juvenile	74.9	-0.55	24.9	36
Rock Bass	2	-0.20	0.12	-0.43	0.04	Cool	Juvenile	104.9	-0.19	24.9	36
Rock Bass	3	-0.05	0.10	-0.24	0.14	Cool	Juvenile	134.7	-0.04	24.9	36
Rock Bass	4	0.04	0.09	-0.14	0.23	Cool	Juvenile	162.3	0.03	24.9	36
Rock Bass	5	0.03	0.10	-0.16	0.23	Cool	Juvenile	186.2	0.02	24.9	36
Rock Bass	6	0.14	0.11	-0.08	0.36	Cool	Adult	207.6	0.07	24.9	36
Rock Bass	7	0.00	0.12	-0.24	0.24	Cool	Adult	224.6	0.00	24.9	36
Rock Bass	8	0.05	0.14	-0.24	0.33	Cool	Adult	241.0	0.02	24.9	36
Rock Bass	9	0.01	0.18	-0.34	0.37	Cool	Adult	253.0	0.00	24.9	36
Rock Bass	10	-0.20	0.23	-0.65	0.25	Cool	Adult	263.7	-0.08	24.9	36
Rock Bass	11	-0.25	0.34	-0.92	0.41	Cool	Adult	272.9	-0.09	24.9	36
Smallmouth Bass	0	0.03	0.31	-0.57	0.64	Warm	Juvenile	92.7	0.04	25	36.3
Smallmouth Bass*	1	-0.68	0.13	-0.95	-0.41	Warm	Juvenile	140.9	-0.48	25	36.3
Smallmouth Bass*	2	-0.27	0.11	-0.48	-0.06	Warm	Juvenile	208.2	-0.13	25	36.3
Smallmouth Bass	3	-0.01	0.10	-0.21	0.18	Warm	Juvenile	268.1	-0.01	25	36.3
Smallmouth Bass	4	0.16	0.11	-0.06	0.38	Warm	Juvenile	323.7	0.05	25	36.3
Smallmouth Bass	5	0.18	0.13	-0.07	0.42	Warm	Juvenile	363.6	0.05	25	36.3
Smallmouth Bass	6	0.16	0.14	-0.11	0.43	Warm	Adult	397.0	0.04	25	36.3
Smallmouth Bass	7	-0.12	0.16	-0.43	0.19	Warm	Adult	423.2	-0.03	25	36.3
Smallmouth Bass	8	0.07	0.18	-0.29	0.43	Warm	Adult	444.6	0.02	25	36.3
Smallmouth Bass	9	-0.02	0.23	-0.48	0.43	Warm	Adult	463.2	0.00	25	36.3
Smallmouth Bass	10	-0.35	0.29	-0.92	0.21	Warm	Adult	480.5	-0.07	25	36.3
Pumpkinseed Sunfish*	1	-0.39	0.11	-0.62	-0.17	Warm	Juvenile	77.4	-0.51	27.7	37.6
Pumpkinseed Sunfish*	2	-0.17	0.07	-0.31	-0.02	Warm	Juvenile	105.2	-0.16	27.7	37.6
Pumpkinseed Sunfish	3	0.07	0.06	-0.06	0.19	Warm	Juvenile	130.4	0.05	27.7	37.6
Pumpkinseed Sunfish*	4	0.21	0.07	0.08	0.34	Warm	Adult	151.1	0.14	27.7	37.6
Pumpkinseed Sunfish*	5	0.29	0.08	0.14	0.44	Warm	Adult	168.1	0.17	27.7	37.6
Pumpkinseed Sunfish*	6	0.29	0.09	0.10	0.47	Warm	Adult	180.9	0.16	27.7	37.6

Pumpkinseed Sunfish*	7	0.36	0.12	0.12	0.60	Warm	Adult	191.9	0.19	27.7	37.6
Pumpkinseed Sunfish	8	0.31	0.19	-0.06	0.68	Warm	Adult	204.1	0.15	27.7	37.6
Pumpkinseed Sunfish	9	0.13	0.26	-0.38	0.65	Warm	Adult	211.9	0.06	27.7	37.6
Pumpkinseed Sunfish	10	0.29	0.41	-0.51	1.09	Warm	Adult	217.5	0.13	27.7	37.6
Largemouth Bass*	0	-0.53	0.18	-0.87	-0.18	Warm	Juvenile	88.5	-0.60	28.6	38.4
Largemouth Bass*	1	-0.85	0.08	-1.01	-0.70	Warm	Juvenile	142.2	-0.60	28.6	38.4
Largemouth Bass*	2	-0.56	0.06	-0.69	-0.44	Warm	Juvenile	203.8	-0.28	28.6	38.4
Largemouth Bass*	3	-0.41	0.06	-0.53	-0.30	Warm	Juvenile	258.4	-0.16	28.6	38.4
Largemouth Bass*	4	-0.32	0.07	-0.45	-0.19	Warm	Juvenile	300.5	-0.11	28.6	38.4
Largemouth Bass*	5	-0.37	0.08	-0.52	-0.22	Warm	Juvenile	336.3	-0.11	28.6	38.4
Largemouth Bass*	6	-0.59	0.09	-0.76	-0.41	Warm	Adult	367.2	-0.16	28.6	38.4
Largemouth Bass*	7	-0.82	0.10	-1.03	-0.62	Warm	Adult	398.0	-0.21	28.6	38.4
Largemouth Bass*	8	-0.86	0.13	-1.12	-0.61	Warm	Adult	425.7	-0.20	28.6	38.4
Largemouth Bass*	9	-0.86	0.15	-1.17	-0.56	Warm	Adult	451.1	-0.19	28.6	38.4
Largemouth Bass*	10	-0.68	0.19	-1.04	-0.31	Warm	Adult	467.3	-0.15	28.6	38.4
Largemouth Bass*	11	-0.53	0.26	-1.04	-0.03	Warm	Adult	481.1	-0.11	28.6	38.4
Largemouth Bass	12	-0.64	0.33	-1.28	0.00	Warm	Adult	490.8	-0.13	28.6	38.4
Bluegill	0	-0.23	0.29	-0.81	0.35	Warm	Juvenile	56.9	-0.40	30.2	40.2
Bluegill*	1	-0.55	0.08	-0.71	-0.39	Warm	Juvenile	72.1	-0.76	30.2	40.2
Bluegill*	2	-0.34	0.06	-0.46	-0.21	Warm	Juvenile	104.3	-0.32	30.2	40.2
Bluegill*	3	-0.22	0.06	-0.33	-0.11	Warm	Juvenile	131.3	-0.17	30.2	40.2
Bluegill	4	-0.05	0.06	-0.16	0.07	Warm	Juvenile	154.7	-0.03	30.2	40.2
Bluegill*	5	0.15	0.06	0.03	0.28	Warm	Adult	173.4	0.09	30.2	40.2
Bluegill*	6	0.28	0.07	0.14	0.42	Warm	Adult	188.3	0.15	30.2	40.2
Bluegill*	7	0.23	0.09	0.06	0.40	Warm	Adult	200.6	0.11	30.2	40.2
Bluegill	8	0.18	0.12	-0.05	0.41	Warm	Adult	211.5	0.09	30.2	40.2
Bluegill	9	0.10	0.17	-0.23	0.42	Warm	Adult	222.8	0.04	30.2	40.2
Bluegill	10	0.06	0.22	-0.37	0.49	Warm	Adult	231.0	0.02	30.2	40.2
Bluegill	11	-0.05	0.31	-0.66	0.56	Warm	Adult	242.5	-0.02	30.2	40.2

Table S3. Effect of year on total length in mm per species age class from Bayesian hierarchical models summarized per species life stage (juvenile and adult) as the mean and standard deviation (SD) for that species life stage expressed as the slope (annual change in length in mm) and annual percent change relative to the mean total length for a given species age class.

Species	Thermal Guild	Juvenile				Adult			
		Annual Change in Length (mm)		Annual Percent Change		Annual Change in Length (mm)		Annual Percent Change	
		Mean	SD	Mean	SD	Mean	SD	Mean	SD
Cisco	Cold	-0.04	0.25	-0.02	0.08	-0.31	NA	*-0.08*	NA
Rainbow Trout	Cold	1.15	1.01	0.27	0.20	*NA*	*NA*	*NA*	*NA*
Brown Trout	Cold	-0.35	0.36	-0.12	0.15	*NA*	*NA*	*NA*	*NA*
Yellow Perch	Cool	-0.35	0.17	-0.31	0.23	-0.18	0.14	-0.07	0.05
Northern Pike	Cool	-0.96	0.40	-0.26	0.20	-2.61	0.98	-0.33	0.11
Walleye	Cool	-0.15	0.40	-0.09	0.16	-0.35	0.51	-0.06	0.09
Black Crappie	Cool	-0.19	0.15	-0.13	0.12	-0.11	0.26	-0.03	0.09
Common White Sucker	Cool	-0.85	0.34	-0.27	0.06	-0.75	*NA*	-0.17	*NA*
Rock Bass	Cool	-0.12	0.19	-0.15	0.24	-0.04	0.15	-0.01	0.06
Smallmouth Bass	Warm	-0.10	0.33	-0.08	0.21	-0.05	0.20	-0.01	0.04
Pumpkinseed Sunfish	Warm	-0.16	0.23	-0.20	0.28	0.27	0.07	0.14	0.04
Largemouth Bass	Warm	-0.51	0.19	-0.31	0.23	-0.71	0.14	-0.16	0.04
Bluegill	Warm	-0.28	0.18	-0.34	0.28	0.14	0.11	0.07	0.06

Table S4. Effect of year on total length in mm per species age class from Bayesian hierarchical models summarized per age class summarized as the mean and standard deviation (SD) expressed as both the slope (annual change in length in mm) and that slope expressed as percent change relative to mean total length of a given species age class.

Age	Annual Change in Length (mm)		Annual Percent Change in Length	
	Mean	SD	Mean	SD
0	-0.45	0.51	-0.35	0.28
1	-0.59	0.31	-0.41	0.19
2	-0.27	0.43	-0.14	0.16
3	-0.14	0.50	-0.06	0.14
4	0.06	0.83	0.01	0.18
5	-0.13	0.51	0.00	0.12
6	-0.14	0.72	0.00	0.14
7	-0.37	0.90	-0.05	0.16
8	-0.52	1.31	-0.06	0.19
9	-0.56	1.17	-0.08	0.15
10	-0.53	0.70	-0.09	0.11
11	-0.25	0.20	-0.06	0.04
12	-0.77	0.19	-0.14	0.01

Table S5. A) Model results from Bayesian meta-regression that examined if the effect of year on total length from the Bayesian hierarchical model varied among species age classes with respect to final temperature preferendum (FTP). B) Post-hoc test comparing adult and juvenile effects from Bayesian meta-regression to zero. For adults the relationship with FTP was positive and statistically different from zero. The juvenile relationship with FTP did not differ from zero as indicated by the 95% credibility interval overlapping with zero. C) Post-hoc pairwise comparison of the effects of adult and juvenile relationships with FTP. These effects were statistically different from one another as indicated by the credibility interval not overlapping zero.

A.	Estimate	Est. Error	Lower 95% CI	Upper 95% CI	Rhat	Bulk ESS	Tail ESS
Intercept	-1.86	0.10	-2.06	-1.67	1.00	1321	1500
FTP	0.07	0.00	0.06	0.07	1.00	1295	1459
Life Stage - Juvenile	1.53	0.12	1.29	1.77	1.00	1119	1333
FTP x Life Stage - Juvenile	-0.06	0.00	-0.07	-0.05	1.00	1113	1304

B.	FTP Slope	Lower 95% CI	Upper 95% CI
Adult	0.065	0.058	0.073
Juvenile	0.001	-0.005	0.006

C.	Estimate	Lower 95% CI	Upper 95% CI
adult-juvenile	0.064	0.055	0.074

Table S6. Model performance and summary metrics for all boosted regression tree models.

Species	Age	Total Deviance	Residual Deviance	Correlation	Percent Variance Explained	CV Deviance	CV Correlation	CV Percent Variance Explained
Black Crappie	0	550	464	0.48	15.7	525	0.24	4.6
Black Crappie	1	841	446	0.71	47.0	601	0.53	28.5
Black Crappie	2	919	471	0.72	48.8	700	0.49	23.9
Black Crappie	3	1016	715	0.58	29.6	906	0.33	10.8
Black Crappie	4	1126	808	0.60	28.2	1044	0.28	7.3
Black Crappie	5	1261	870	0.59	31.0	1146	0.32	9.1
Black Crappie	6	1235	819	0.64	33.7	1189	0.23	3.7
Black Crappie	7	1290	779	0.67	39.6	1283	0.22	0.6
Black Crappie	8	1245	677	0.73	45.6	1220	0.22	2.0
Black Crappie	9	1047	468	0.78	55.3	986	0.32	5.9
Black Crappie	10	1172	678	0.70	42.2	1143	0.25	2.5
Black Crappie	11	1069	605	0.68	43.4	976	0.39	8.7
White Sucker	1	3483	1751	0.71	49.7	2699	0.56	22.5
White Sucker	2	4402	2143	0.72	51.3	3686	0.41	16.3
White Sucker	3	3562	1566	0.76	56.0	2828	0.49	20.6
White Sucker	4	3231	1547	0.75	52.1	2841	0.36	12.1
White Sucker	5	2749	1613	0.70	41.3	2685	0.30	2.3
Cisco	2	1815	666	0.80	63.3	1106	0.62	39.1
Cisco	3	1534	764	0.72	50.2	1322	0.43	13.8
Cisco	4	1727	877	0.74	49.3	1693	0.30	2.0
Cisco	5	1675	924	0.74	44.8	1593	0.29	4.9
Cisco	6	1272	527	0.81	58.5	1457	-0.01	-14.6
Cisco	7	1424	485	0.84	65.9	1539	0.31	-8.1
Northern Pike	0	3846	1881	0.75	51.1	3473	0.38	9.7
Northern Pike	1	5350	2715	0.71	49.3	3565	0.58	33.4
Northern Pike	2	5223	3576	0.58	31.5	4371	0.41	16.3
Northern Pike	3	5241	3909	0.54	25.4	4685	0.33	10.6
Northern Pike	4	6490	4626	0.57	28.7	5738	0.34	11.6
Northern Pike	5	7856	5524	0.57	29.7	7043	0.33	10.3
Northern Pike	6	9856	6322	0.62	35.9	8630	0.36	12.4
Northern Pike	7	13295	4586	0.82	65.5	13708	0.35	-3.1
Northern Pike	8	17046	3938	0.88	76.9	17089	0.38	-0.3
Northern Pike	9	18041	8398	0.76	53.5	17691	0.29	1.9
Northern Pike	10	17175	10513	0.64	38.8	16437	0.32	4.3
Bluegill	0	379	376	0.45	0.9	391	0.11	-3.2
Bluegill	1	748	275	0.80	63.2	418	0.67	44.1
Bluegill	2	658	320	0.73	51.4	450	0.57	31.6
Bluegill	3	700	288	0.78	58.8	520	0.51	25.6
Bluegill	4	730	375	0.73	48.6	616	0.40	15.7
Bluegill	5	655	337	0.74	48.6	587	0.34	10.5
Bluegill	6	602	305	0.75	49.3	527	0.37	12.6
Bluegill	7	633	345	0.72	45.5	545	0.38	13.9

Bluegill	8	616	247	0.80	60.0	451	0.52	26.7
Bluegill	9	571	308	0.72	45.9	465	0.44	18.5
Bluegill	10	503	240	0.76	52.4	411	0.44	18.3
Bluegill	11	423	421	0.47	0.5	437	0.02	-3.5
Smallmouth Bass	0	615	614	0.42	0.1	627	-0.06	-2.0
Smallmouth Bass	1	1486	1106	0.67	25.6	1172	0.58	21.2
Smallmouth Bass	2	1613	910	0.67	43.6	1325	0.44	17.8
Smallmouth Bass	3	1670	992	0.66	40.6	1417	0.40	15.1
Smallmouth Bass	4	2103	1337	0.63	36.4	1834	0.39	12.8
Smallmouth Bass	5	1760	1000	0.69	43.1	1481	0.41	15.9
Smallmouth Bass	6	1711	856	0.73	49.9	1361	0.44	20.4
Smallmouth Bass	7	1482	673	0.76	54.6	1139	0.48	23.1
Smallmouth Bass	8	1348	671	0.74	50.2	1186	0.20	12.0
Smallmouth Bass	9	1075	525	0.73	51.1	959	0.42	10.7
Smallmouth Bass	10	1402	760	0.72	45.8	1354	0.30	3.4
Largemouth Bass	0	881	514	0.68	41.6	676	0.49	23.3
Largemouth Bass	1	1837	835	0.74	54.5	1057	0.65	42.4
Largemouth Bass	2	1527	1005	0.60	34.2	1209	0.46	20.8
Largemouth Bass	3	1560	1070	0.58	31.4	1272	0.43	18.4
Largemouth Bass	4	1664	1188	0.55	28.6	1421	0.39	14.6
Largemouth Bass	5	1627	1050	0.61	35.4	1300	0.45	20.1
Largemouth Bass	6	1848	1193	0.61	35.4	1532	0.42	17.1
Largemouth Bass	7	1921	1136	0.66	40.9	1542	0.45	19.7
Largemouth Bass	8	2150	1271	0.67	40.9	1881	0.38	12.5
Largemouth Bass	9	2056	1053	0.73	48.8	1881	0.34	8.5
Largemouth Bass	10	1969	886	0.78	55.0	1803	0.35	8.4
Largemouth Bass	11	1530	865	0.71	43.5	1668	0.03	-9.0
Largemouth Bass	12	1397	870	0.69	37.7	1472	0.08	-5.4
Rainbow Trout	1	3226	1349	0.78	58.2	2388	0.52	26.0
Rainbow Trout	2	5431	2847	0.72	47.6	5451	0.24	-0.4
Rainbow Trout	3	7553	4471	0.67	40.8	7852	0.21	-4.0
Rainbow Trout	4	14251	9296	0.59	34.8	14243	0.15	0.1
Yellow Perch	0	566	357	0.66	37.0	492	0.40	13.0
Yellow Perch	1	728	392	0.69	46.2	479	0.58	34.3
Yellow Perch	2	894	489	0.69	45.4	672	0.50	24.8
Yellow Perch	3	869	548	0.63	37.0	725	0.41	16.5
Yellow Perch	4	1040	797	0.50	23.3	914	0.35	12.0
Yellow Perch	5	1237	944	0.51	23.7	1113	0.32	10.1
Yellow Perch	6	1368	1018	0.53	25.6	1263	0.29	7.7
Yellow Perch	7	1399	959	0.59	31.4	1339	0.25	4.3
Yellow Perch	8	1282	749	0.68	41.5	1233	0.26	3.8
Yellow Perch	9	1094	567	0.74	48.1	1131	0.18	-3.3
Yellow Perch	10	817	628	0.60	23.1	769	0.27	5.9
Yellow Perch	11	696	690	0.34	0.8	729	0.00	-4.7
Pumpkinseed Sunfish	1	452	182	0.79	59.7	266	0.64	41.2
Pumpkinseed Sunfish	2	468	285	0.65	39.1	382	0.43	18.5
Pumpkinseed Sunfish	3	509	314	0.67	38.3	449	0.34	11.7
Pumpkinseed Sunfish	4	511	329	0.65	35.7	462	0.32	9.6
Pumpkinseed Sunfish	5	760	625	0.49	17.9	738	0.20	3.0

Pumpkinseed Sunfish	6	508	318	0.67	37.4	460	0.31	9.4
Pumpkinseed Sunfish	7	468	302	0.66	35.5	421	0.33	10.1
Pumpkinseed Sunfish	8	448	323	0.63	28.0	413	0.30	7.8
Pumpkinseed Sunfish	9	395	189	0.76	52.2	330	0.48	16.5
Rock Bass	1	507	269	0.72	46.9	371	0.54	26.8
Rock Bass	2	464	259	0.69	44.3	367	0.47	21.1
Rock Bass	3	523	272	0.73	47.9	464	0.35	11.3
Rock Bass	4	629	169	0.87	73.1	520	0.45	17.3
Rock Bass	5	663	203	0.86	69.4	531	0.45	19.8
Rock Bass	6	725	206	0.87	71.5	624	0.41	13.9
Rock Bass	7	664	249	0.82	62.4	554	0.42	16.5
Rock Bass	8	583	357	0.67	38.7	519	0.35	11.0
Rock Bass	9	520	204	0.81	60.8	432	0.43	17.0
Rock Bass	10	570	229	0.82	59.9	476	0.42	16.5
Rock Bass	11	555	426	0.59	23.1	550	0.24	0.8
Brown Trout	1	2853	1677	0.67	41.2	2212	0.56	22.5
Brown Trout	2	6214	3080	0.74	50.4	5606	0.30	9.8
Brown Trout	3	7536	3604	0.74	52.2	6310	0.43	16.3
Brown Trout	4	7381	4352	0.66	41.0	6854	0.37	7.1
Brown Trout	5	7396	6090	0.49	17.7	7140	0.32	3.5
Walleye	0	1531	575	0.80	62.5	1009	0.59	34.1
Walleye	1	3072	1164	0.80	62.1	1862	0.63	39.4
Walleye	2	2878	1391	0.73	51.7	2103	0.53	26.9
Walleye	3	3054	1500	0.73	50.9	2078	0.57	31.9
Walleye	4	3270	1617	0.72	50.6	2314	0.54	29.2
Walleye	5	3206	1614	0.72	49.7	2414	0.51	24.7
Walleye	6	3760	1865	0.72	50.4	2832	0.49	24.7
Walleye	7	4111	1920	0.74	53.3	3137	0.50	23.7
Walleye	8	4249	2039	0.74	52.0	3423	0.45	19.4
Walleye	9	3910	1763	0.77	54.9	3229	0.45	17.4
Walleye	10	4702	1986	0.78	57.8	3866	0.44	17.8
Walleye	11	4227	2283	0.70	46.0	4494	0.11	-6.3
Walleye	12	6002	3358	0.72	44.1	6192	0.22	-3.2

Supplementary Figures

Figure S1. Loess fits for each species age class through time to examine if there were any obvious shifts in length trends through time after 2015 when Michigan DNR aging protocols started to incorporate other aging structures in addition to scales for larger fishes (Table S1). The vertical red dashed line is placed at 2015. We were unable to discern any clear, consistent patterns that may be from this change in aging protocol.

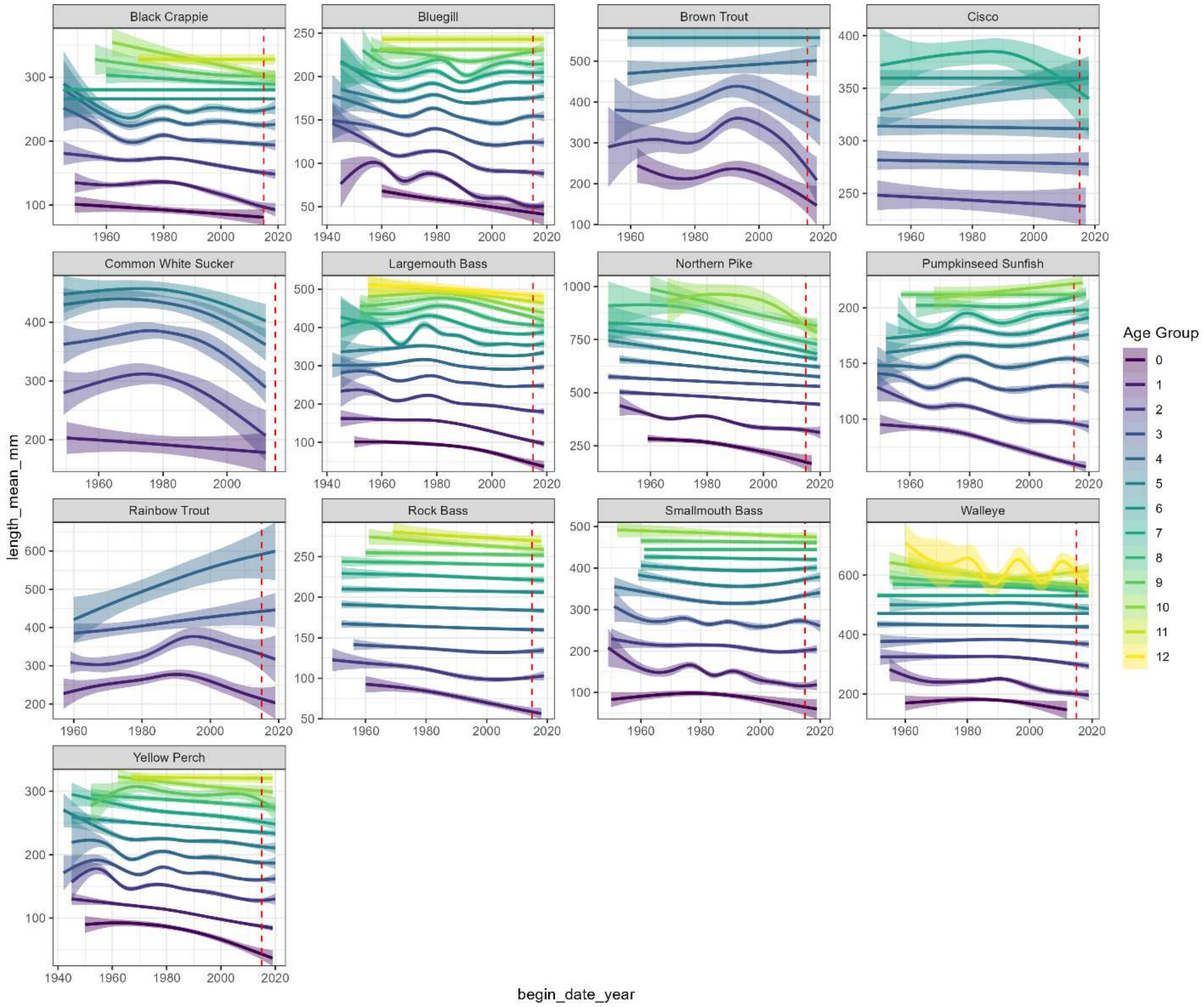

Figure S2. Distribution of mean annual lake surface water temperature across species and age classes.

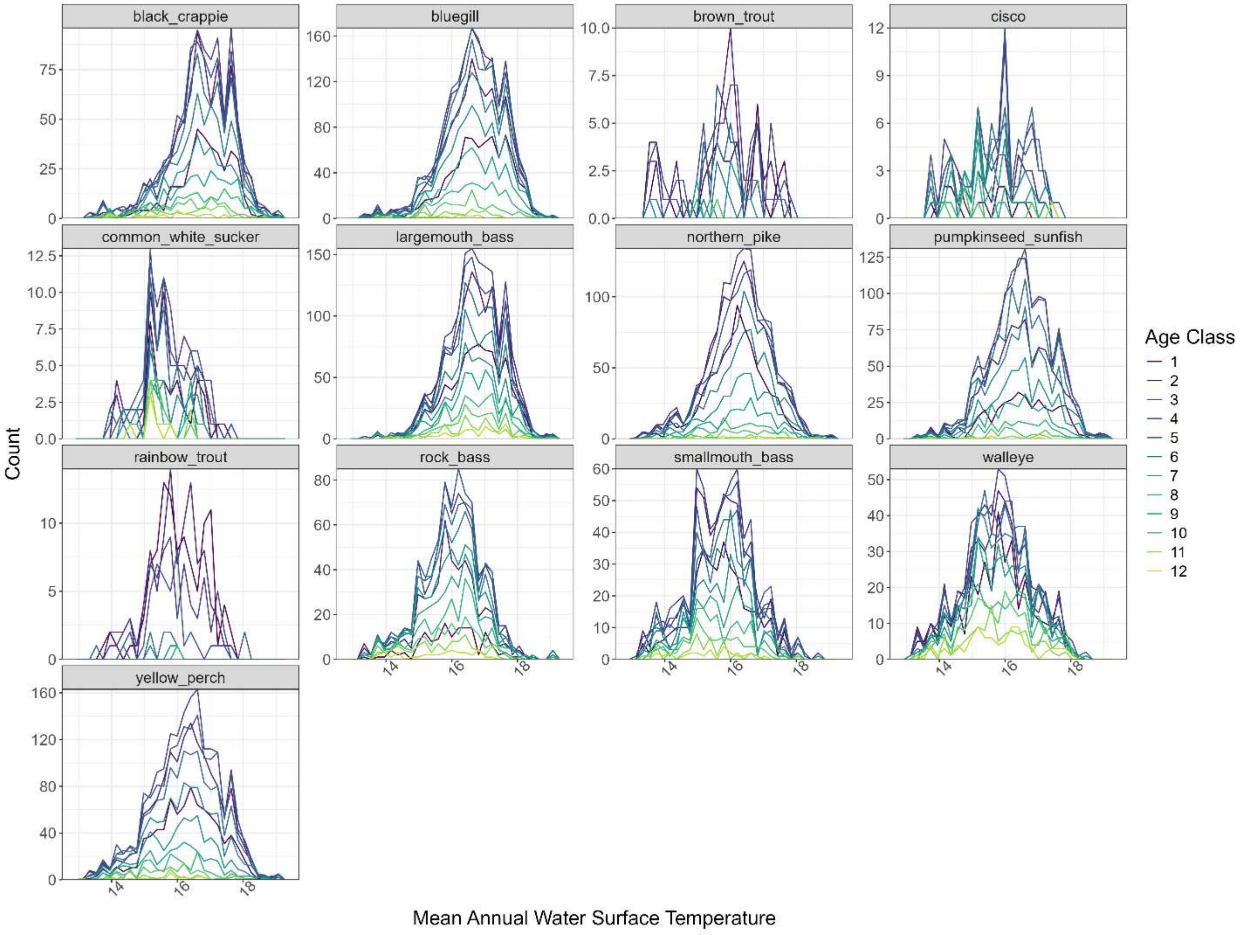

Figure S3. Distribution of mean lifetime growing degree days across species and age classes.

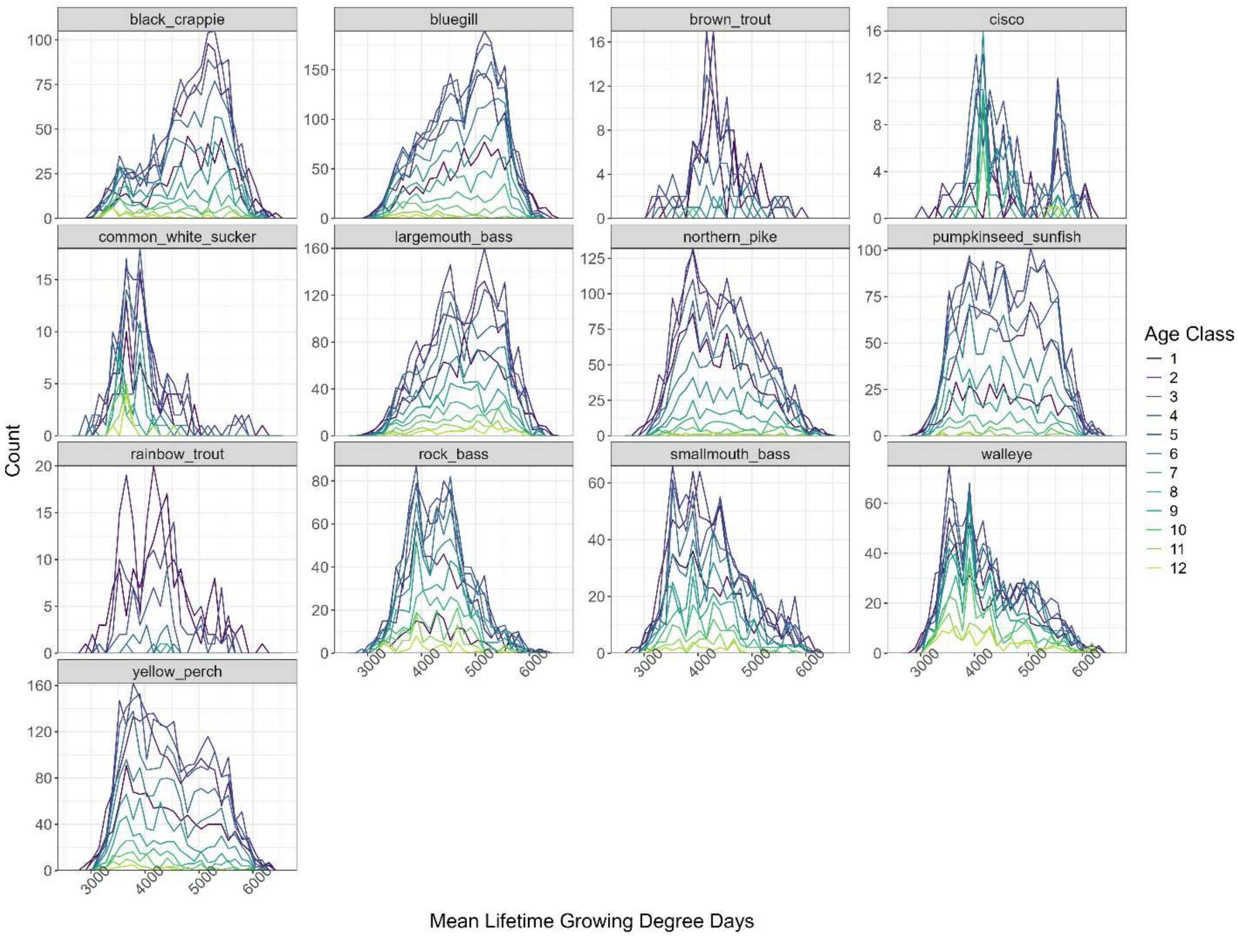

Figure S4. Distribution of day of year across species and age classes.

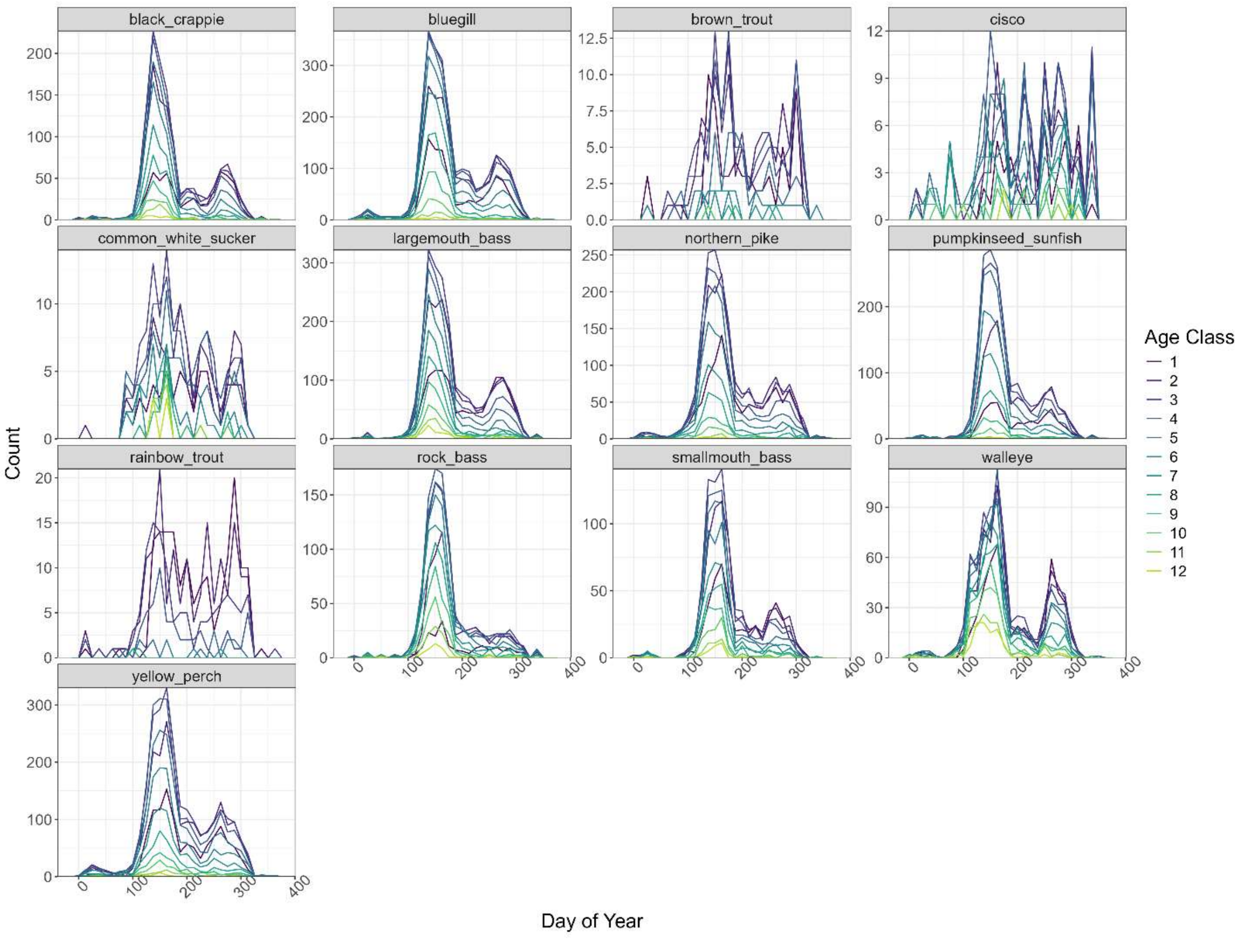

Figure S5. Distribution of log_{10}-transformed lake areas across species and age classes.

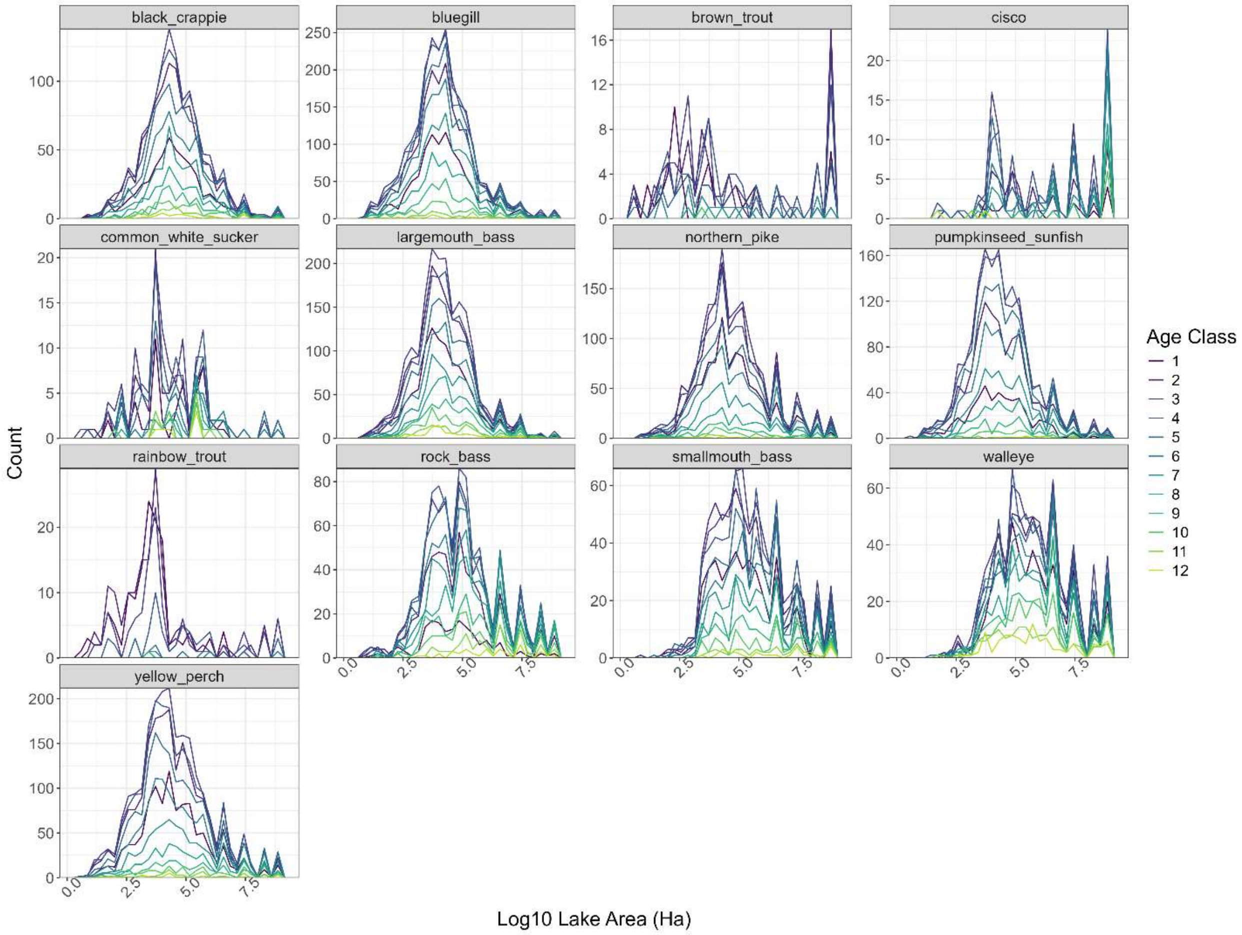

Figure S6. Distribution of $\log_{10}$-transformed lake maximum depth across species and age classes.

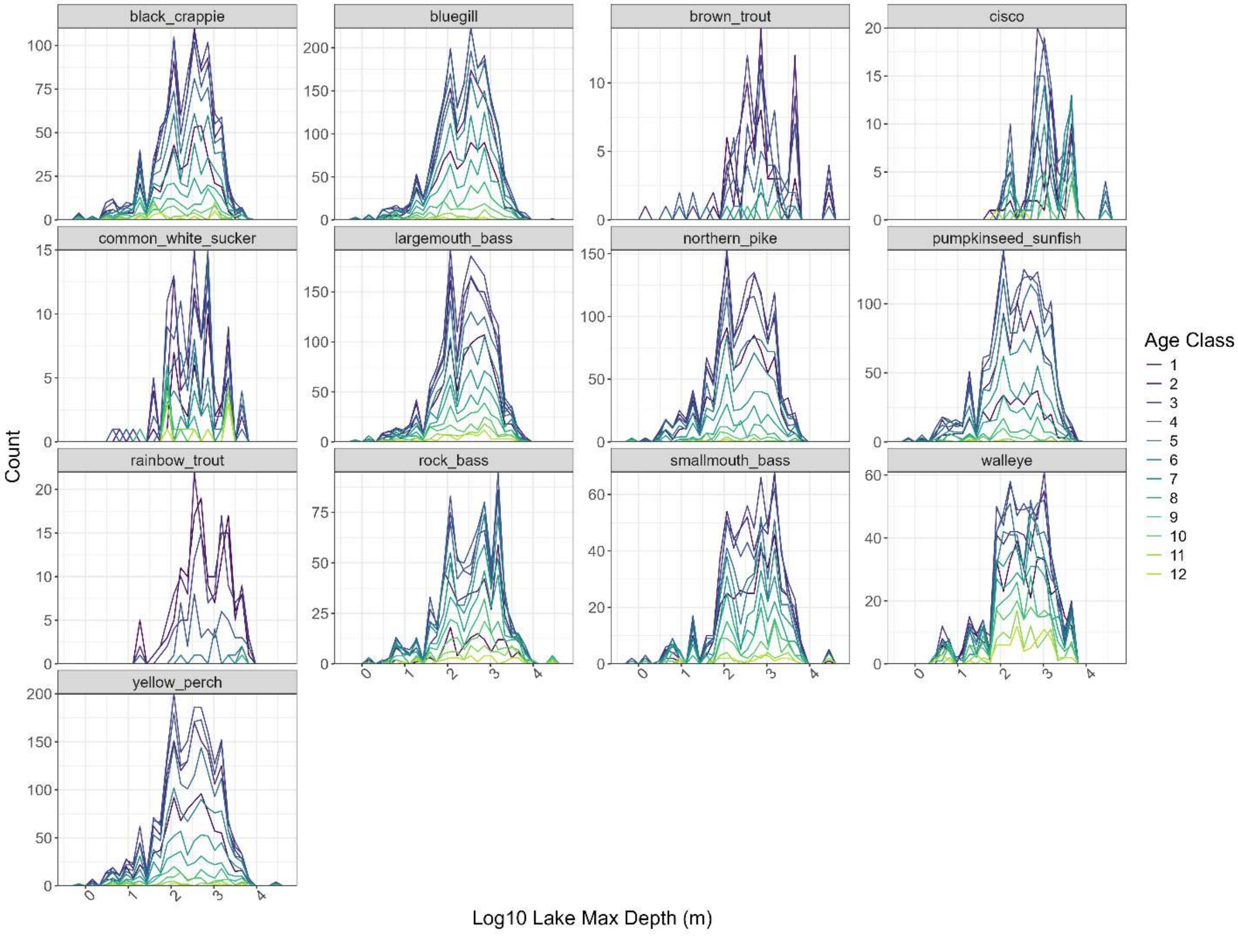

Figure S7. Distribution of Secchi depth across species and age classes.

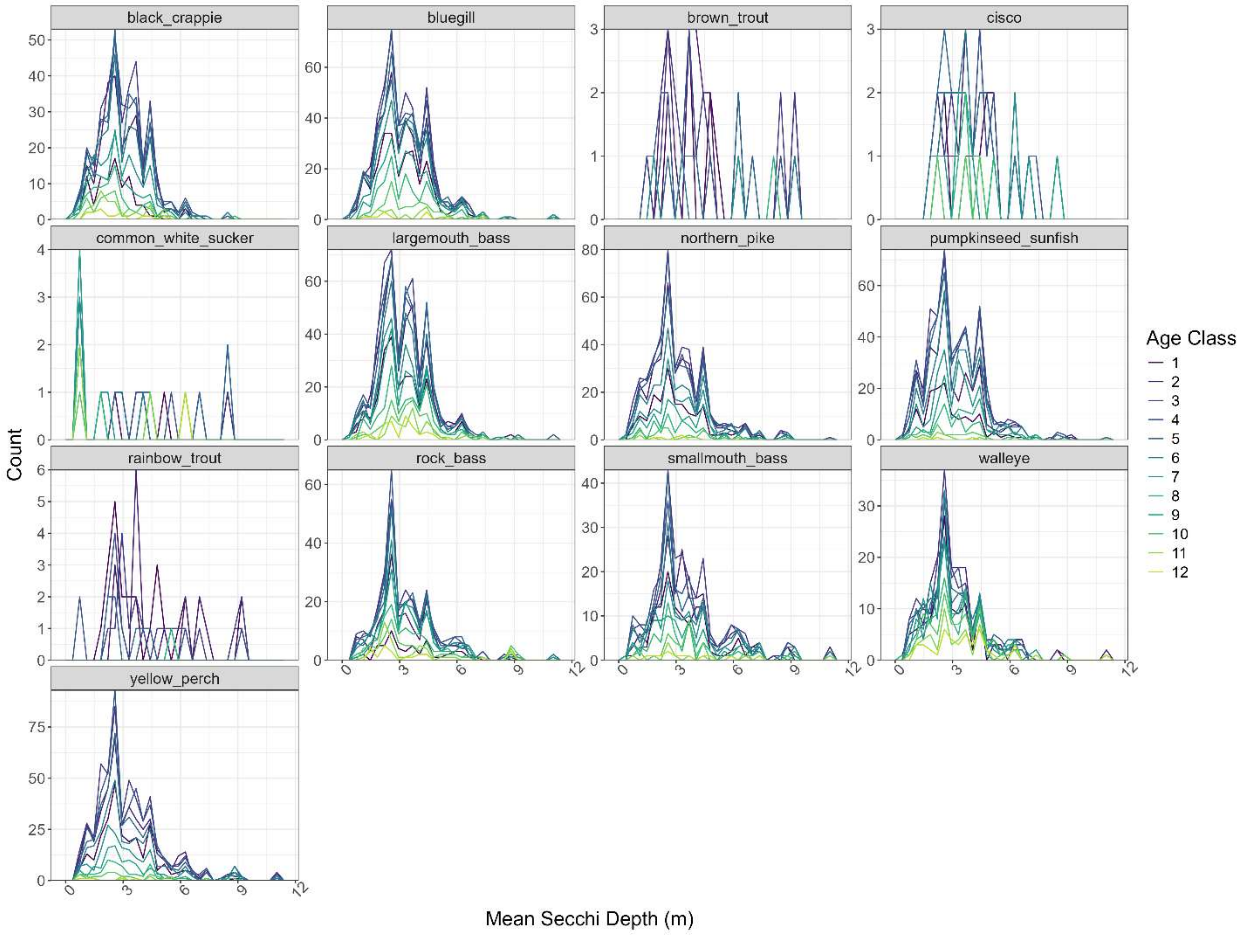

Figure S8. Distribution of percent wetland land cover across species and age classes.

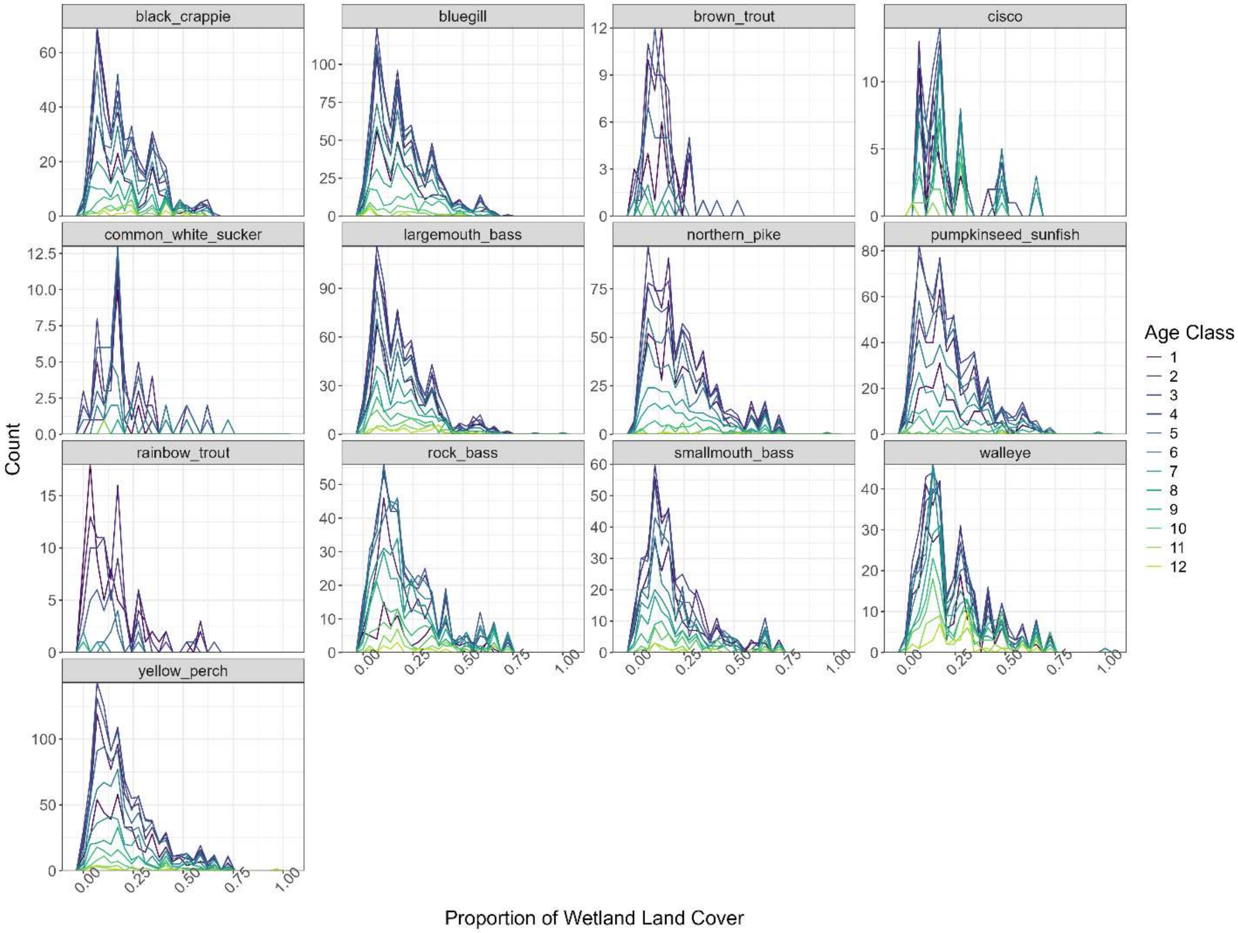

Figure S9. Distribution of percent urban land cover across species and age classes.

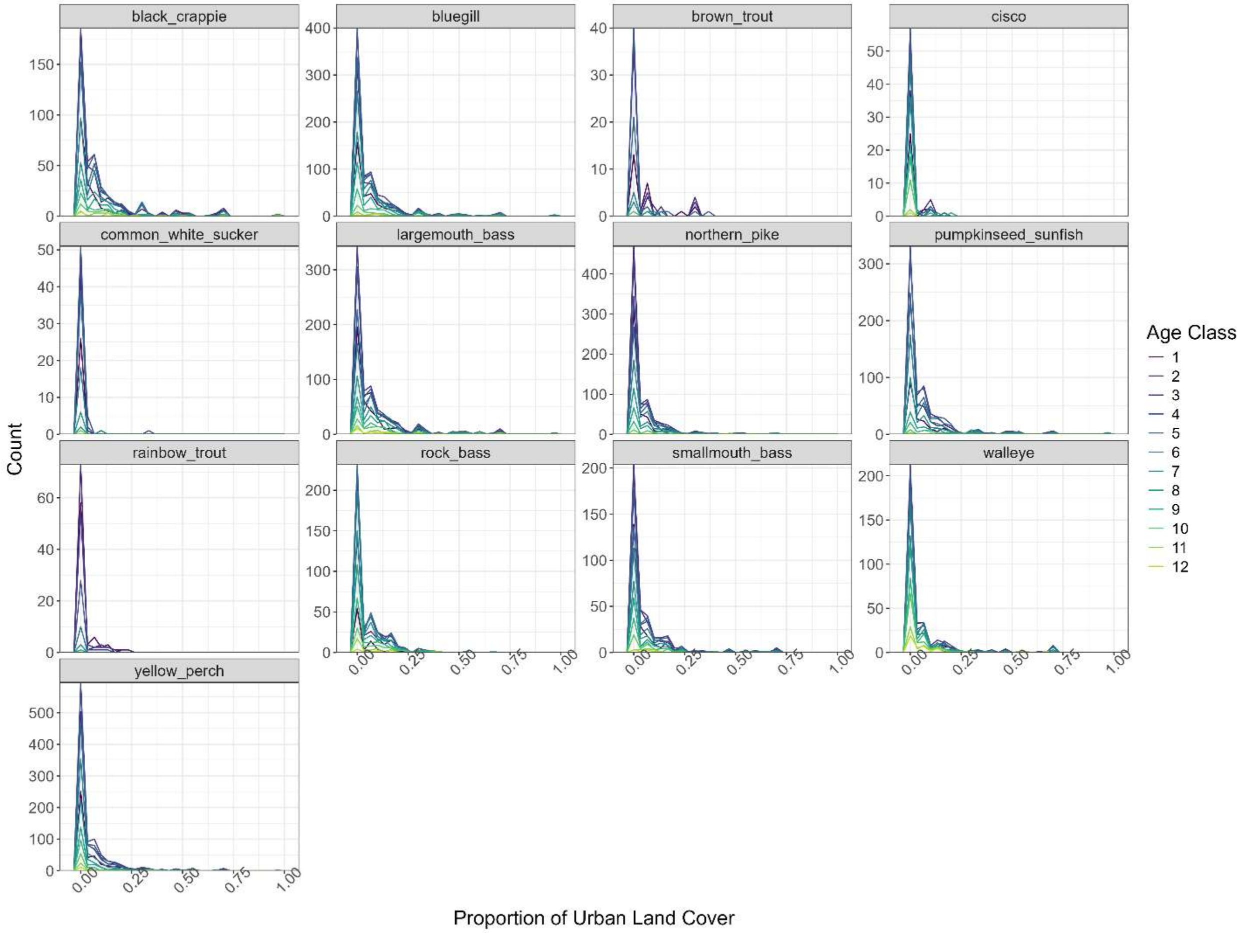

Figure S10. Distribution of percent agricultural land cover across species and age classes.

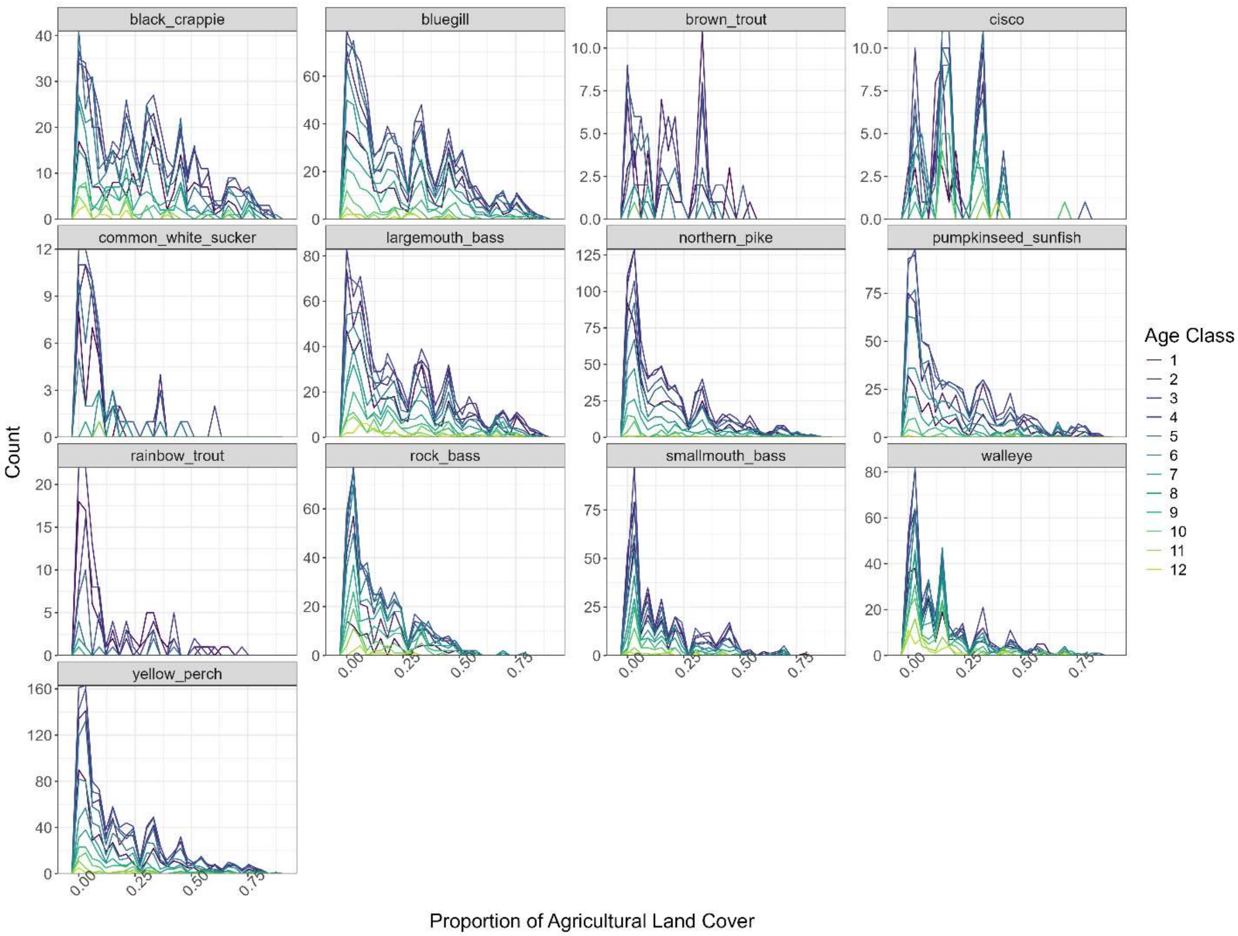

Figure S11. Distribution of percent forest land cover across species and age classes.

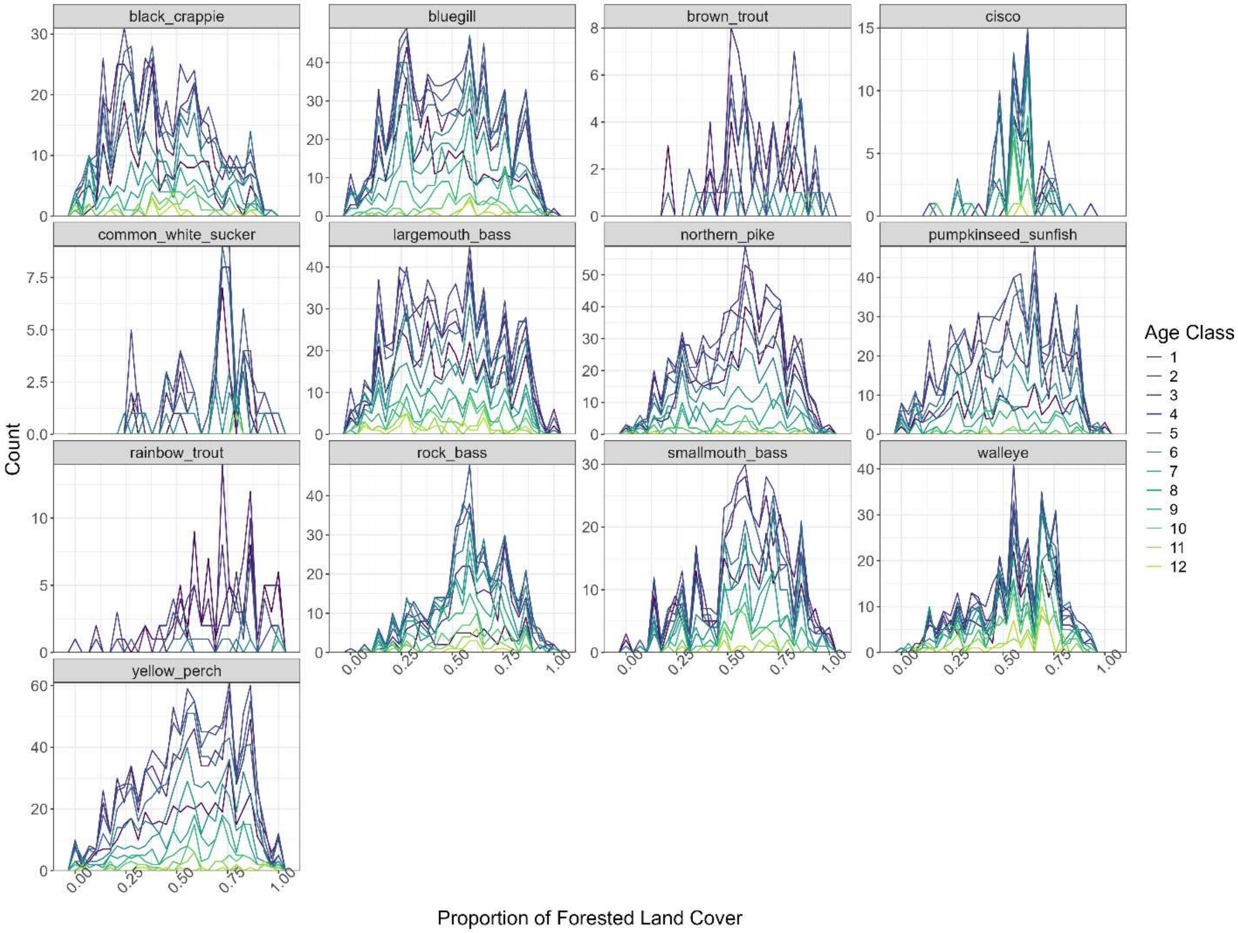

Figure S12. Partial effects of year on length-at-age (total length in mm) from the Bayesian hierarchical model with the partial effect expressed as mean annual percent change in length-at-age. Species are arranged along the x-axis by ascending final temperature preferendum. Statistical differences were based on whether the 95% credibility interval did or did not overlap with zero (as indicated by point shape). Black points and lines represent species means and 95% confidence intervals around those means. Points are labeled with their age group.

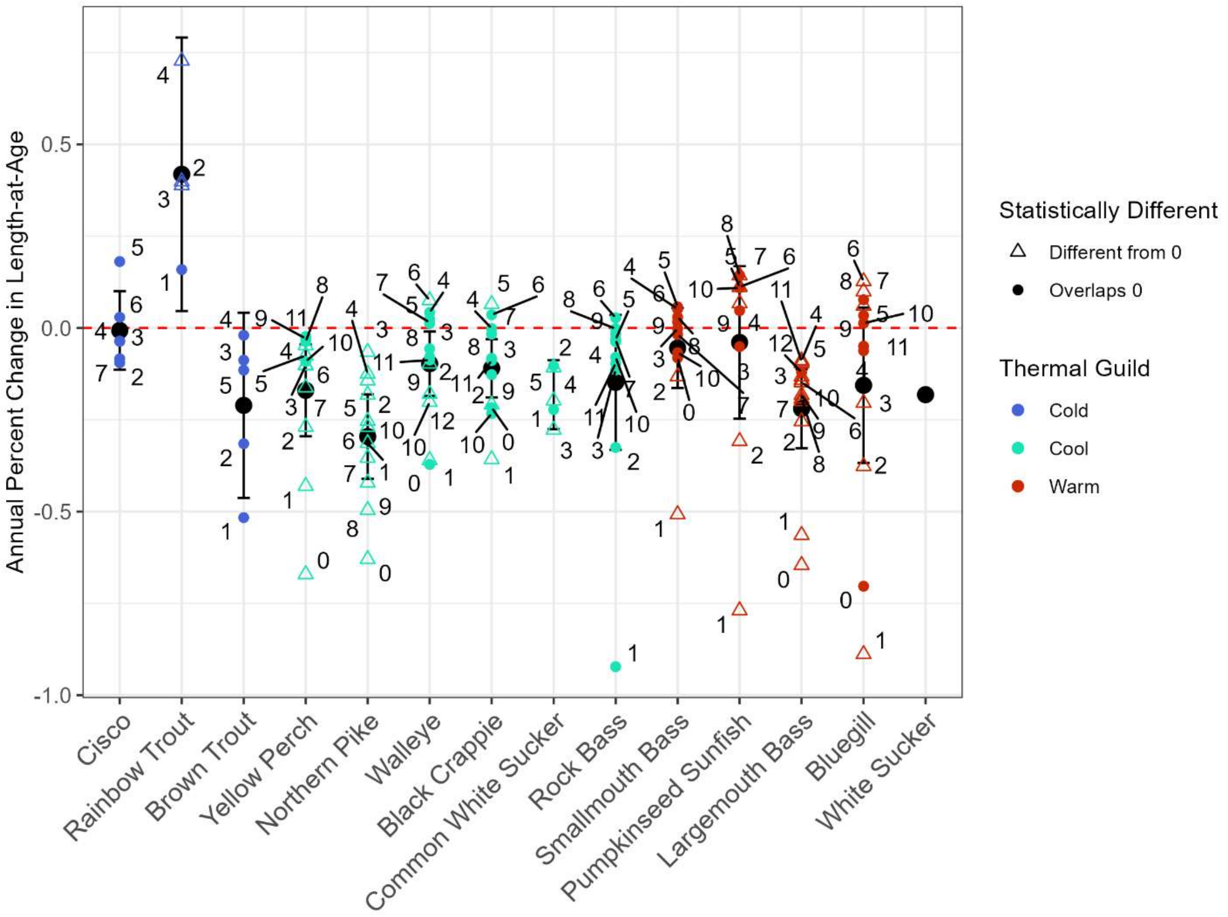

Figure S13. Partial effects of year on length-at-age (total length in mm) from Bayesian hierarchical modeling with the partial effect expressed as mean annual percent change in length-at-age relative to mean total length per species age group. Species panels are ordered by ascending final temperature preferendum starting on the top left. Statistical differences were based on whether the 95% credibility interval did or did not overlap with zero (indicated by point shape).

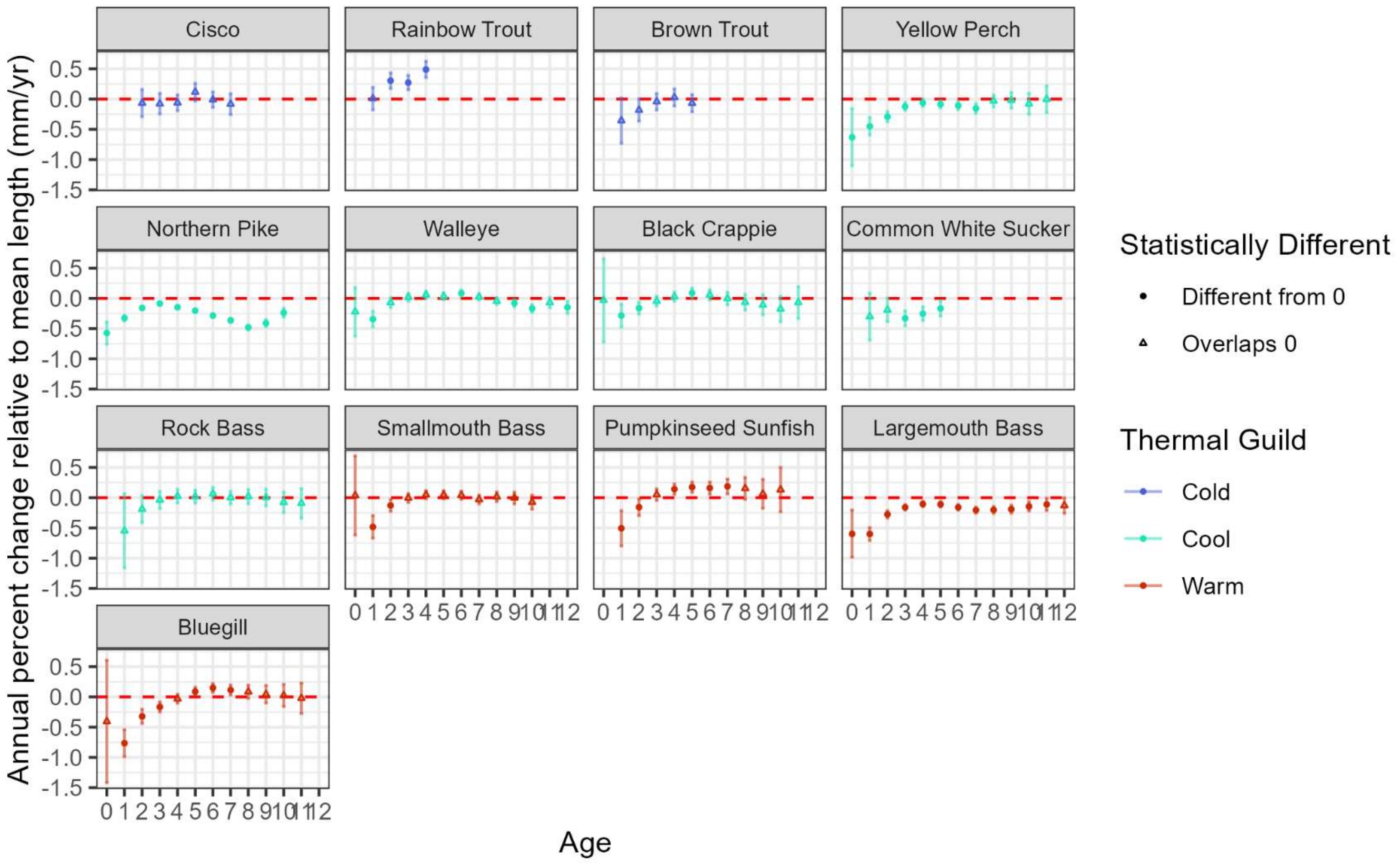

Figure S14. The relationship between the partial effect of year on total length from the Bayesian hierarchical model expressed as annual percent change relative to mean total length for a given species age group A) with and B) without Northern Pike and Rainbow Trout removed with a trend line. Removal of these two species does not change the relationships between FTP and life stage when the partial effect of year on total length is expressed as percent change. Predicted effect of FTP on change in total length (mm) per year from Bayesian meta-regression C) with and D) without Northern Pike and Rainbow Trout. When not expressed as percent change, removing these species changes the relationship between life stage and FTP such that the slope for the adult response to FTP was no longer statistically different from zero (adult 95% credibility interval overlaps zero) or juveniles (95% credibility intervals for adults and juveniles overlap). Meta-regression was not performed on effects expressed as percent change to facilitate propagation of error from the hierarchical model.

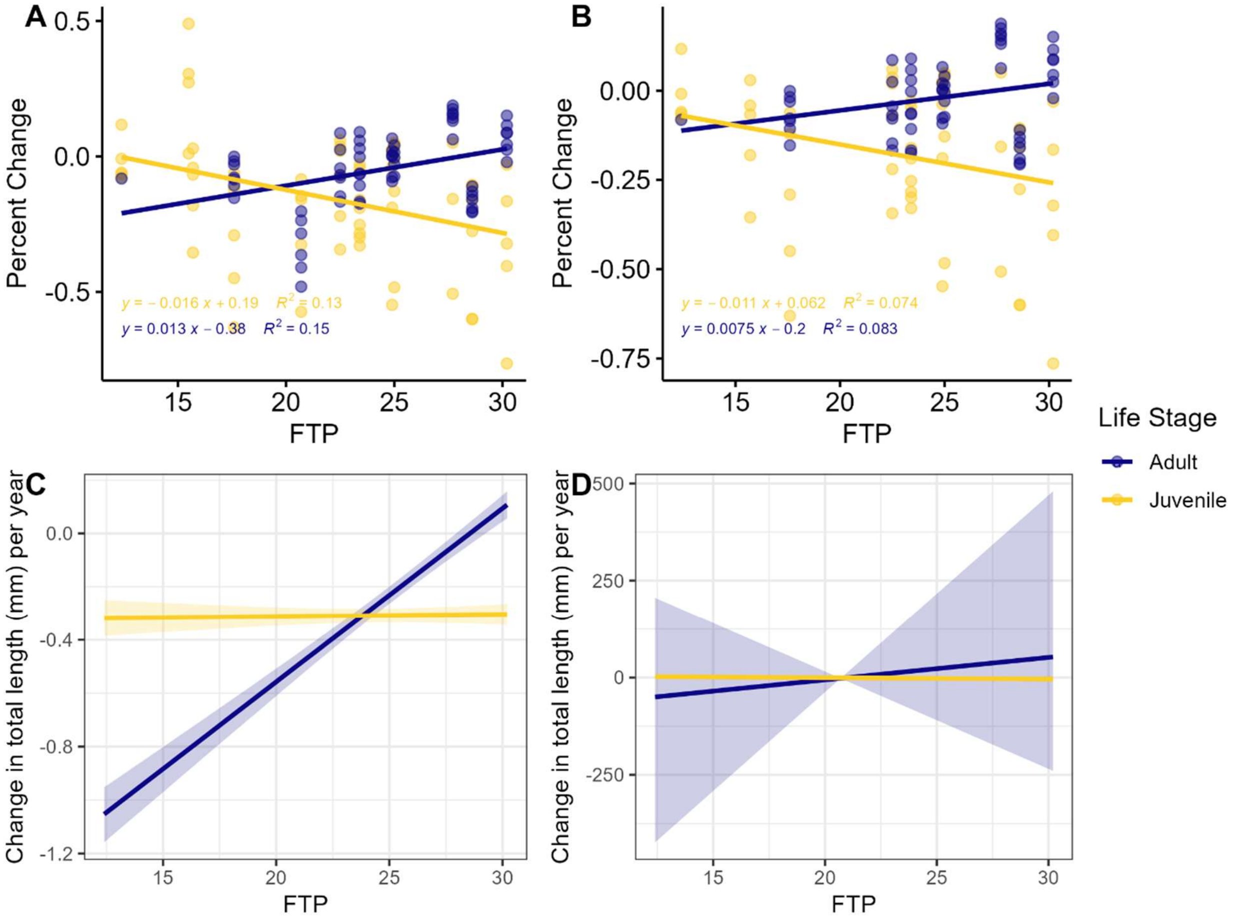

Figure S15. Relative influence of predictors from boosted regression trees for Cisco per age class.

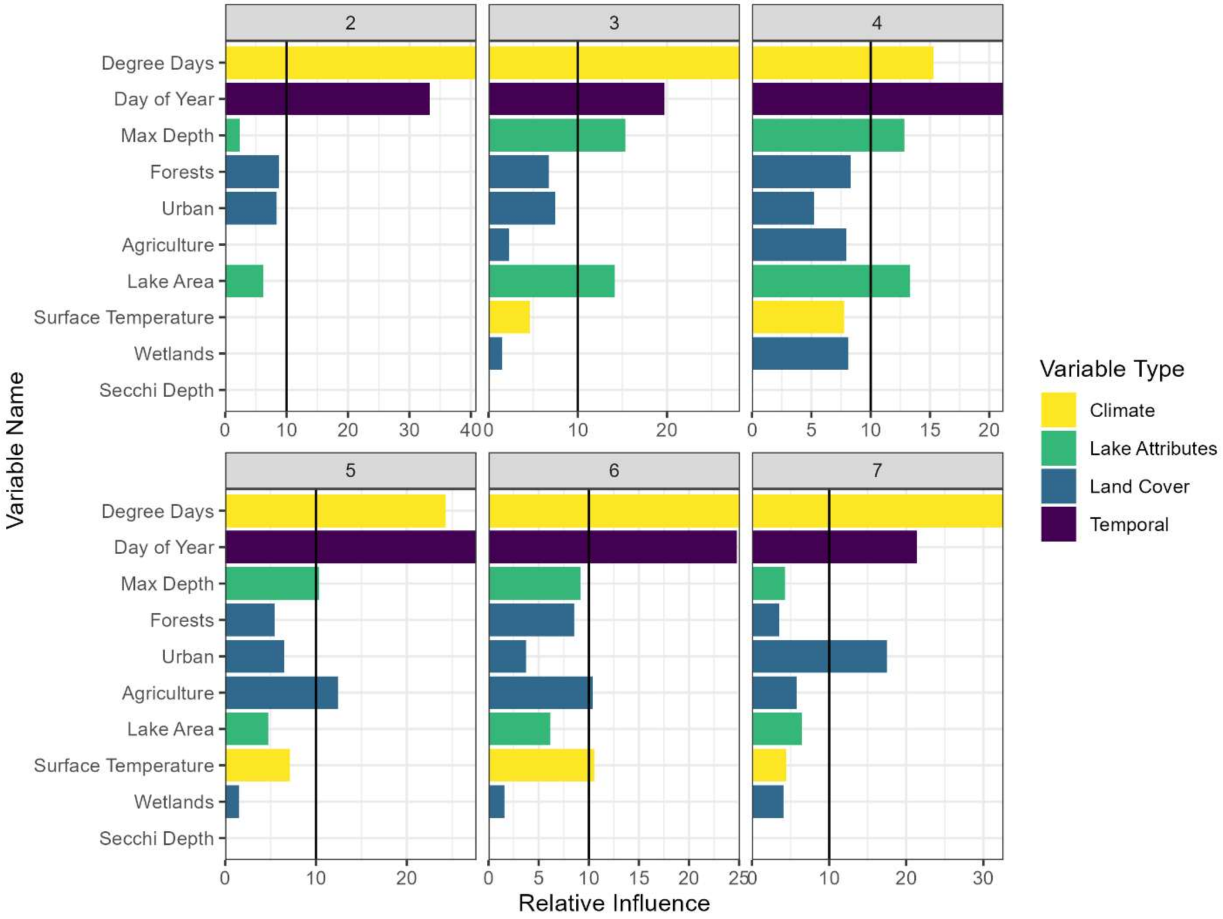

Figure S16. Partial dependency plots for predictors from boosted regression trees for Cisco per age class.

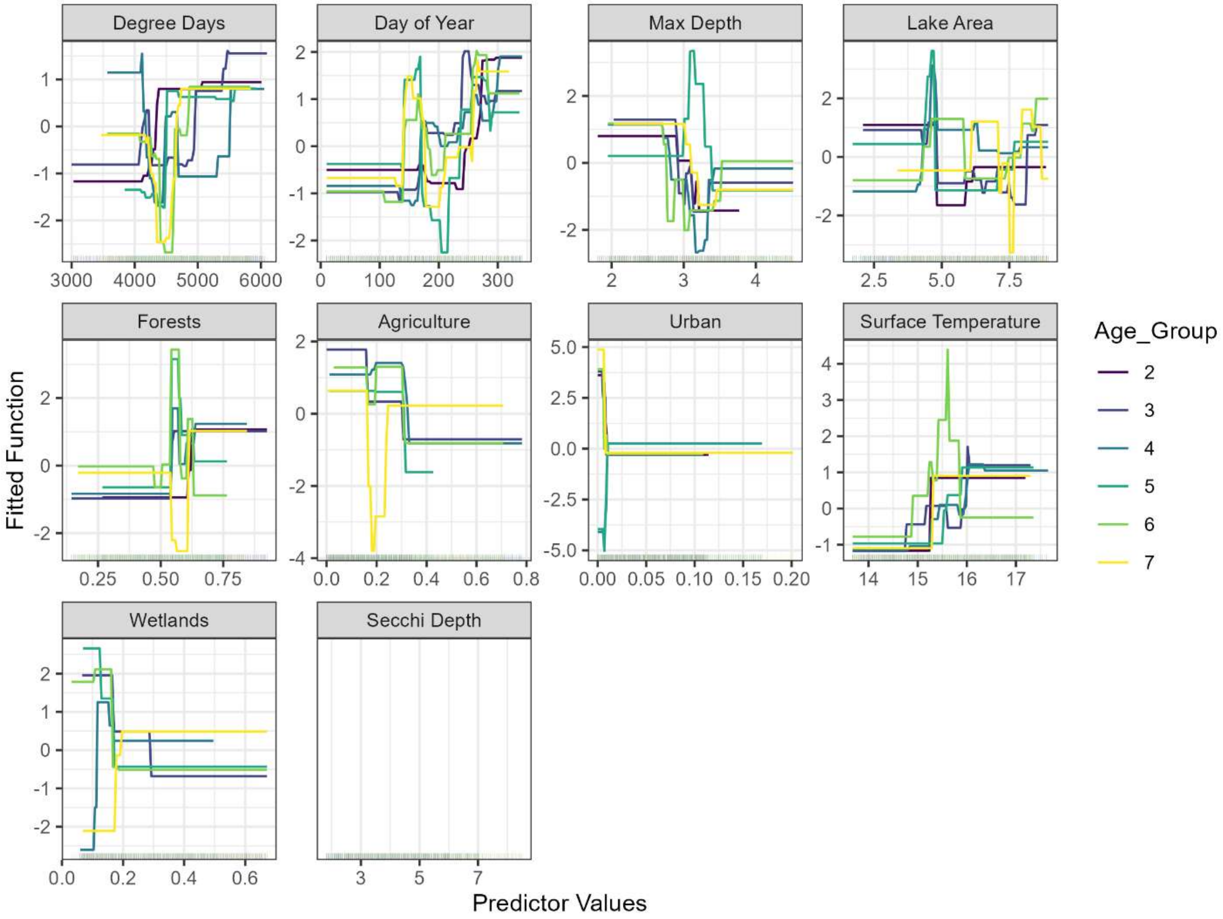

Figure S17. Relative influence of predictors from boosted regression trees for Rainbow Trout per age class.

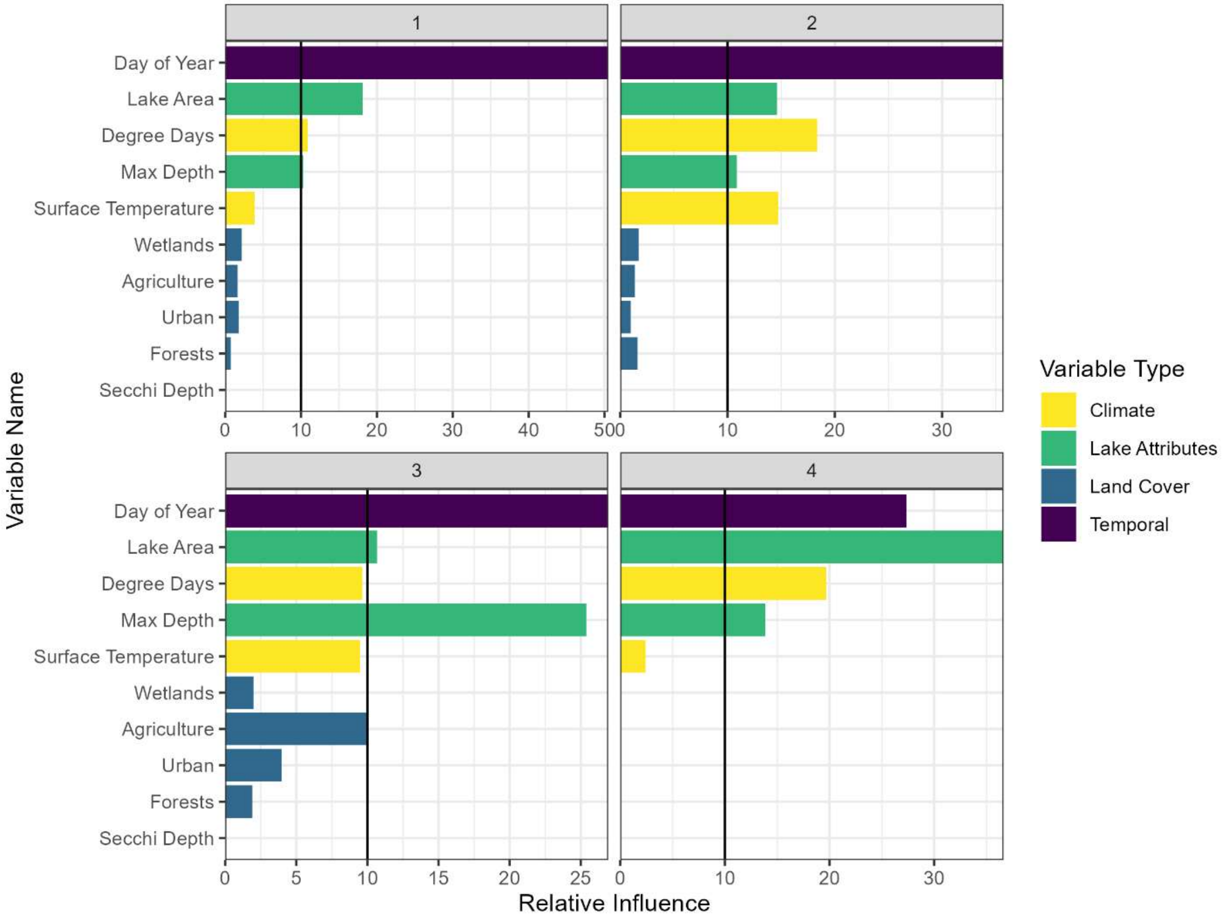

Figure S18. Partial dependency plots for predictors from boosted regression trees for Rainbow Trout per age class.

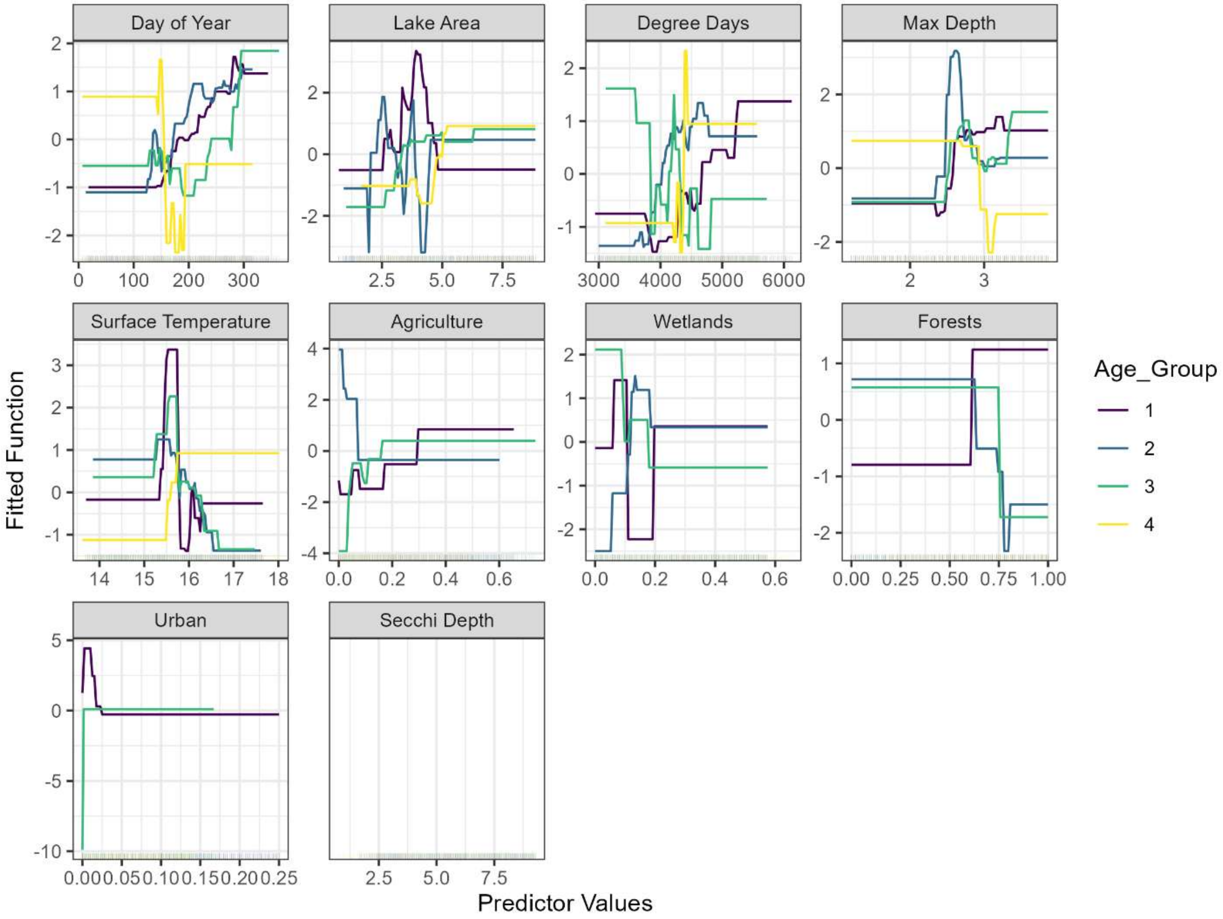

Figure S19. Relative influence of predictors from boosted regression trees for Brown Trout per age class.

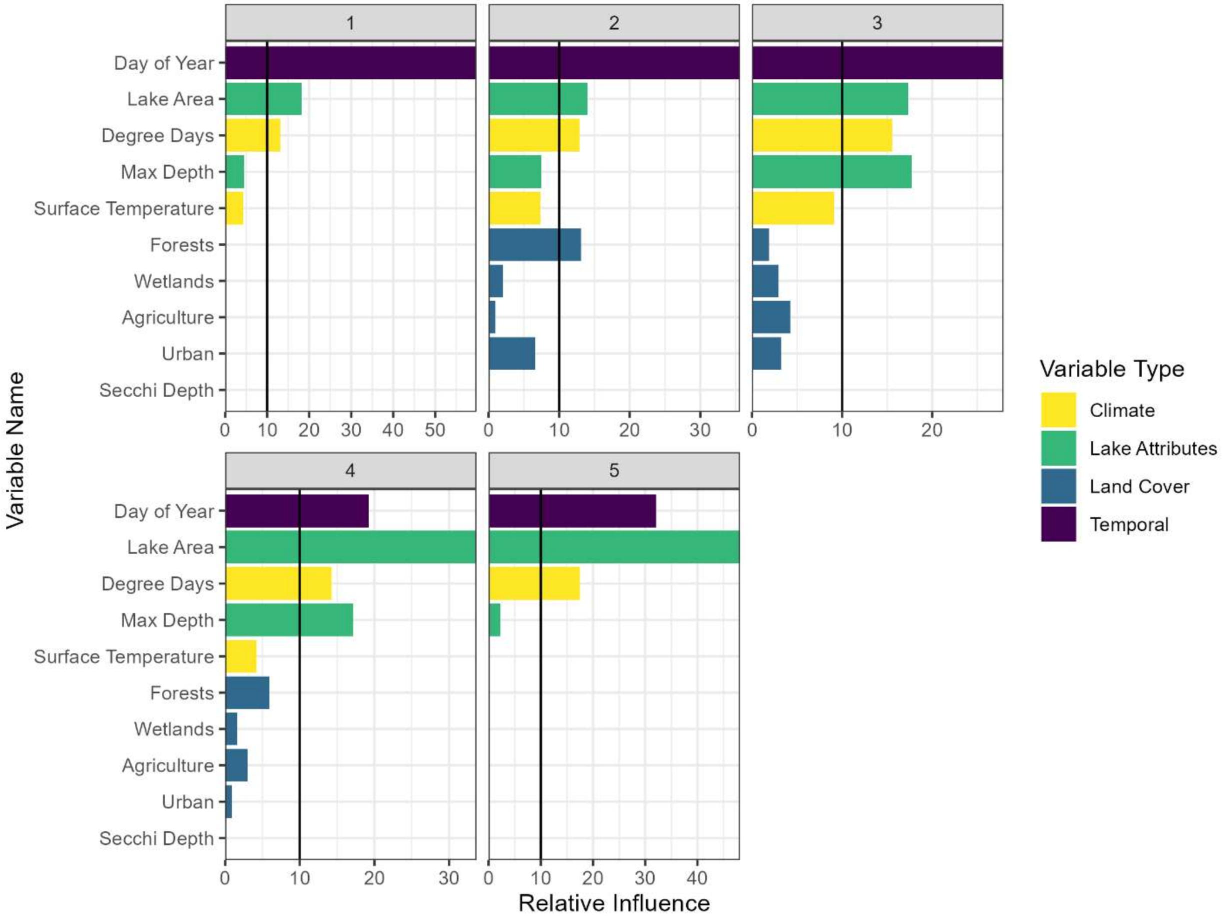

Figure S20. Partial dependency plots for predictors from boosted regression trees for Brown Trout per age class.

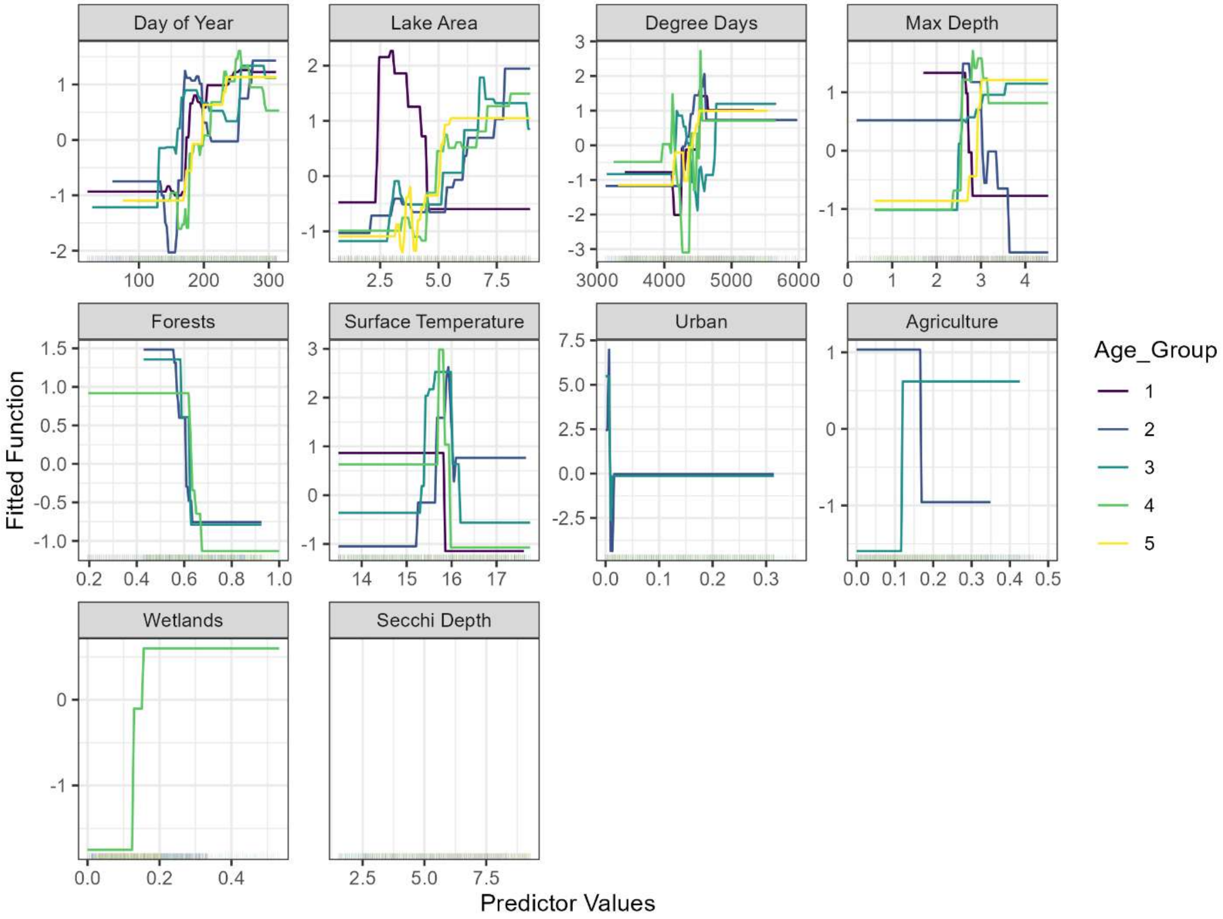

Figure S21. Relative influence of predictors from boosted regression trees for Yellow Perch per age class.

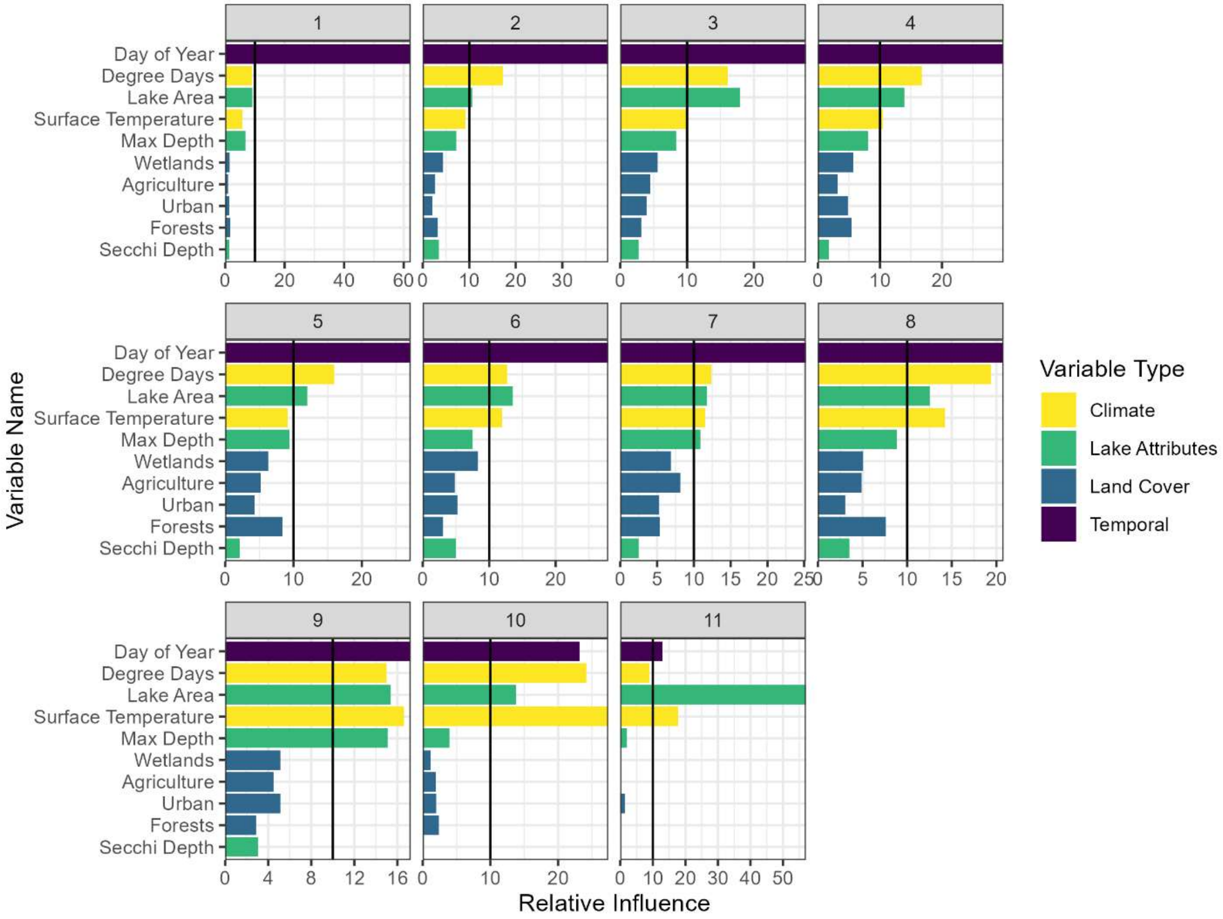

Figure S22. Partial dependency plots for predictors from boosted regression trees for Yellow Perch per age class.

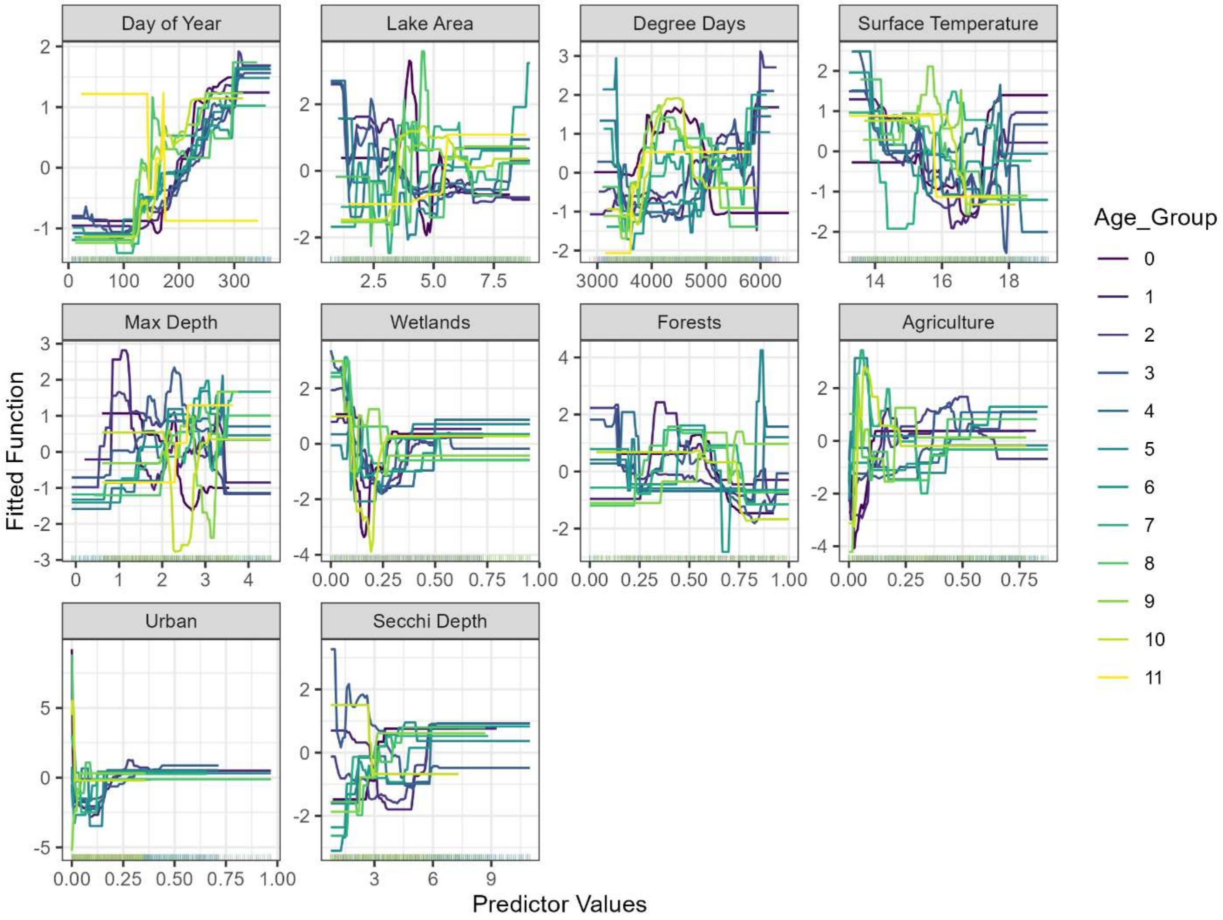

Figure S23. Relative influence of predictors from boosted regression trees for Northern Pike per age class.

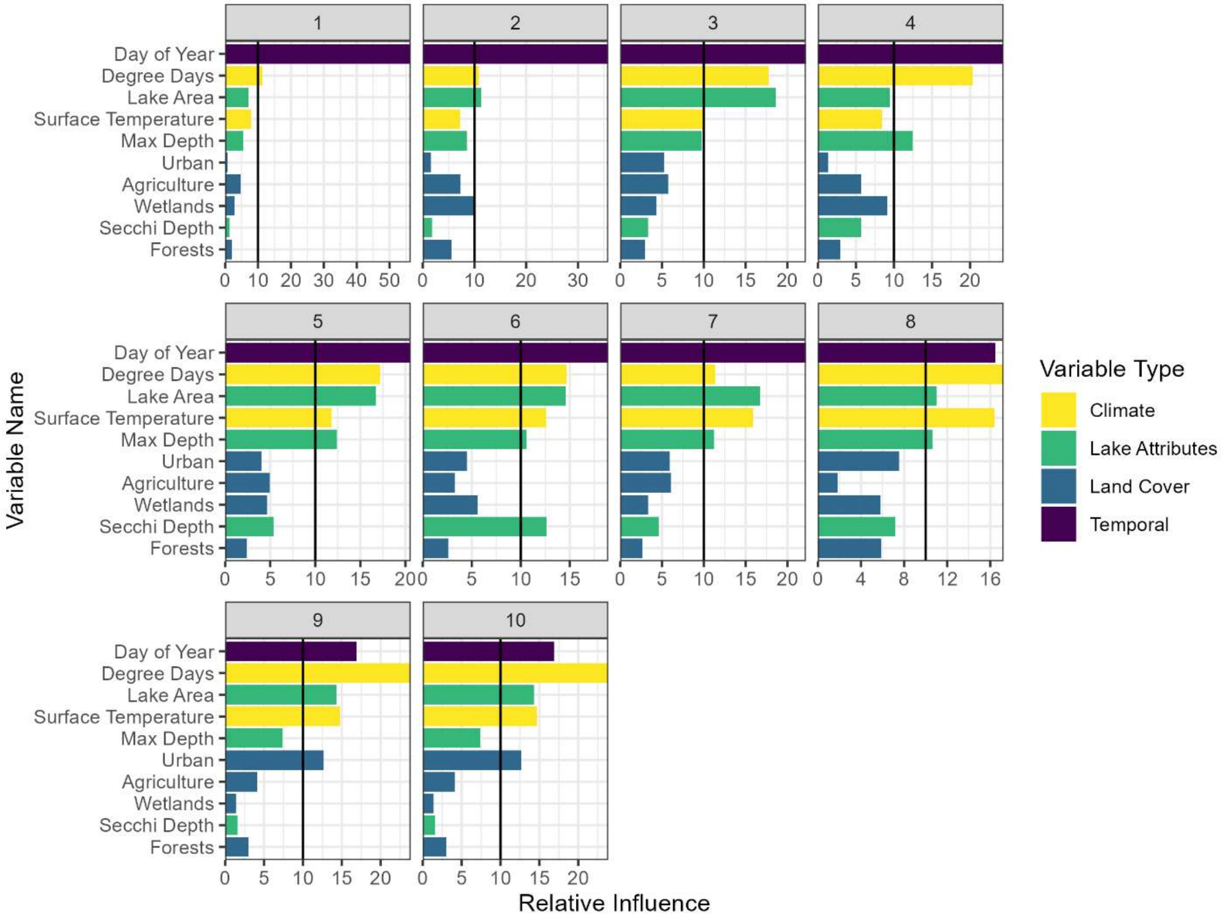

Figure S24. Partial dependency plots for predictors from boosted regression trees for Northern Pike per age class.

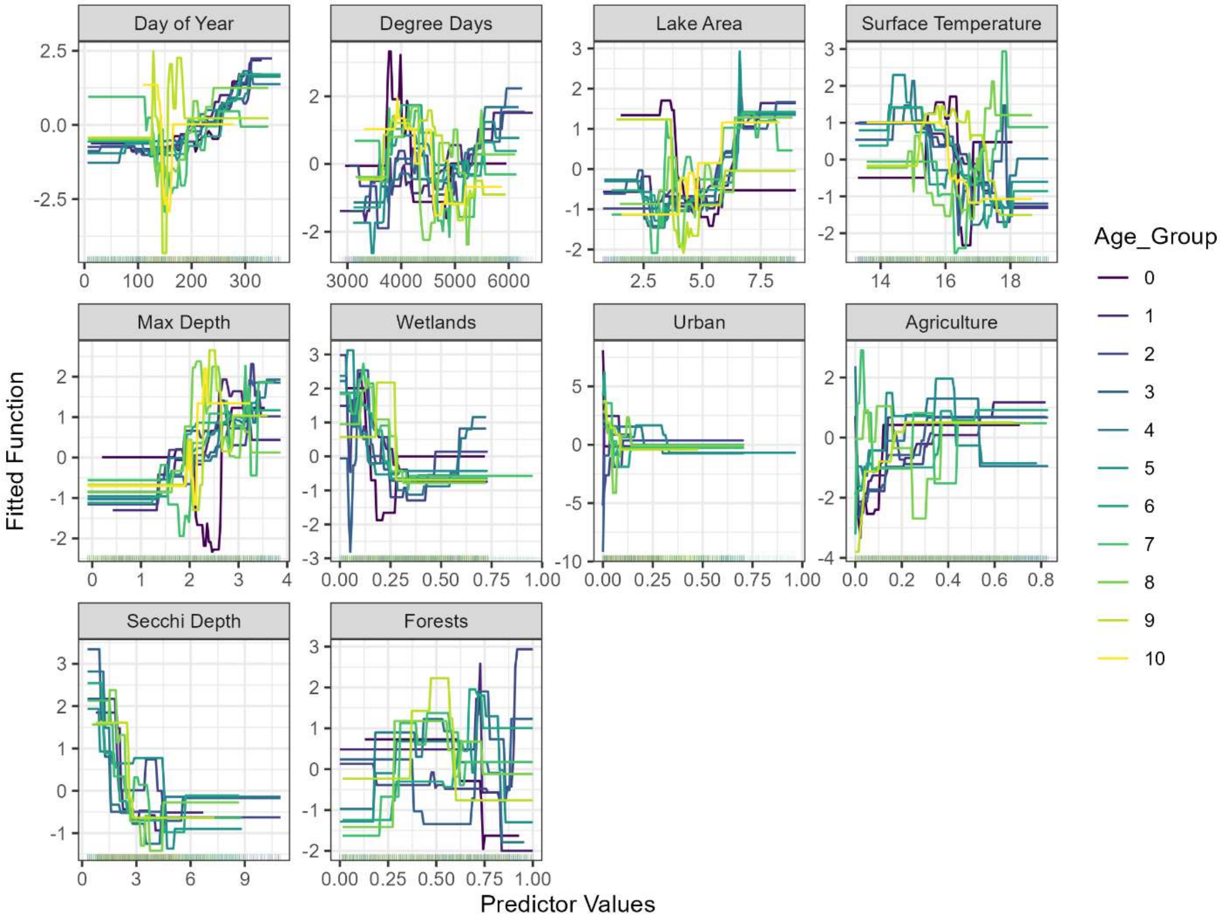

Figure S25. Relative influence of predictors from boosted regression trees for White Sucker per age class.

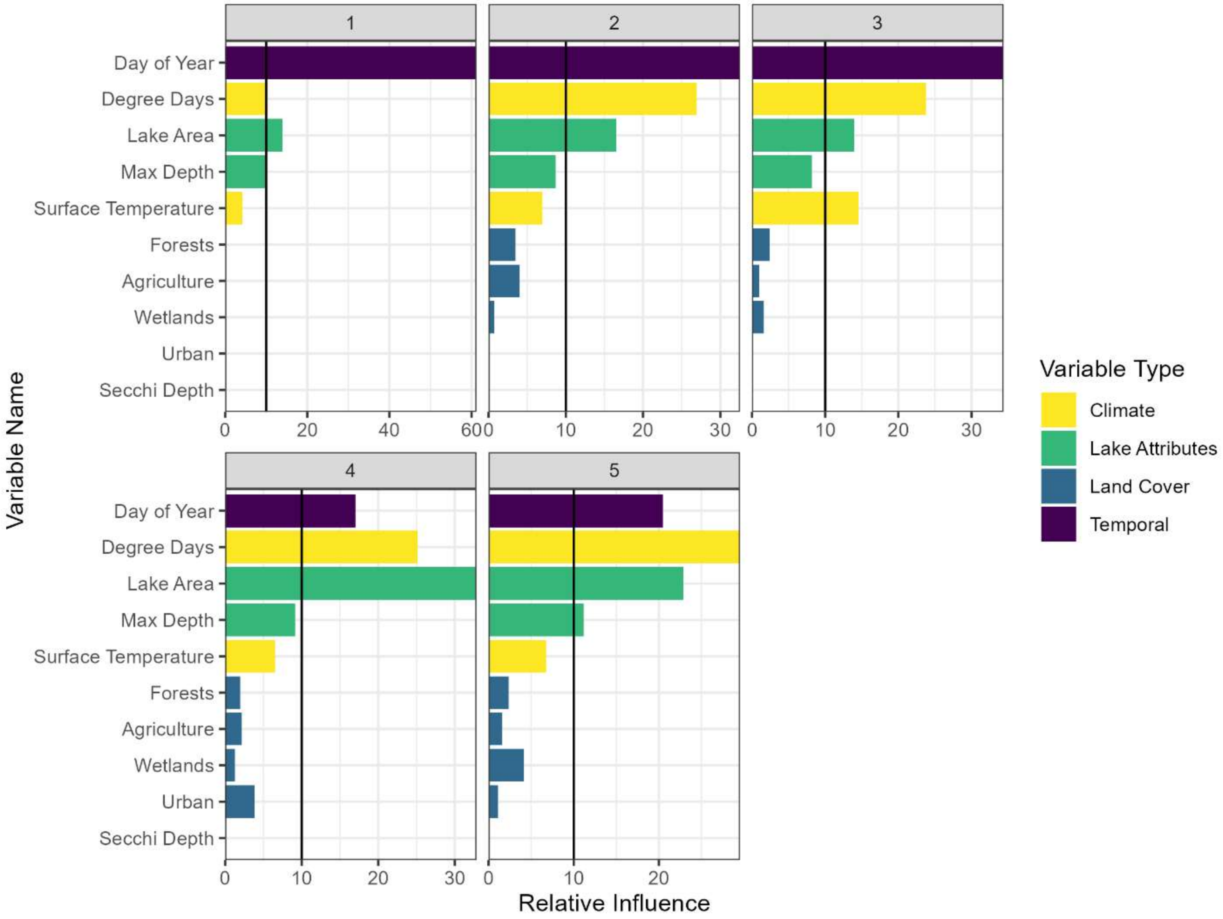

Figure S26. Partial dependency plots for predictors from boosted regression trees for White Sucker per age class.

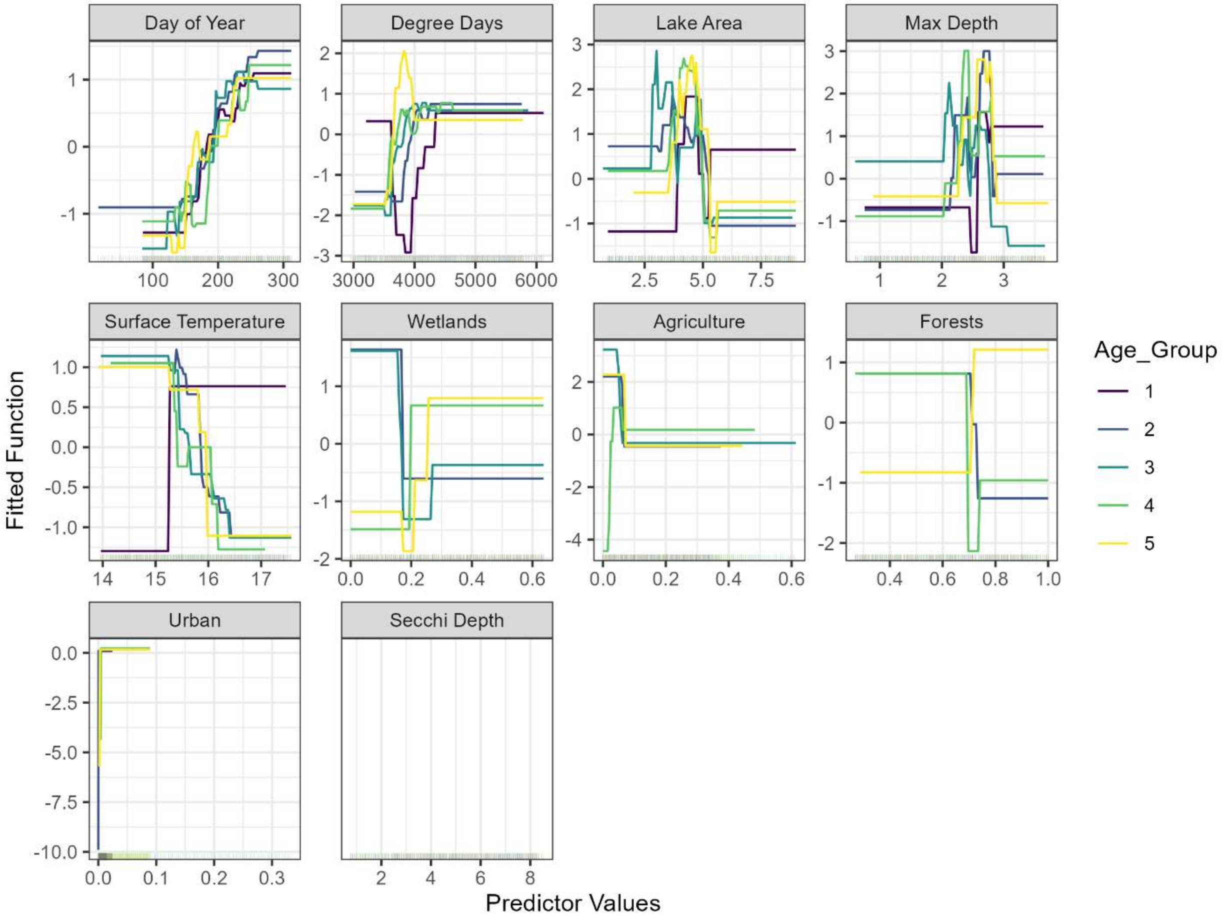

Figure S27. Relative influence of predictors from boosted regression trees for Black Crappie per age class.

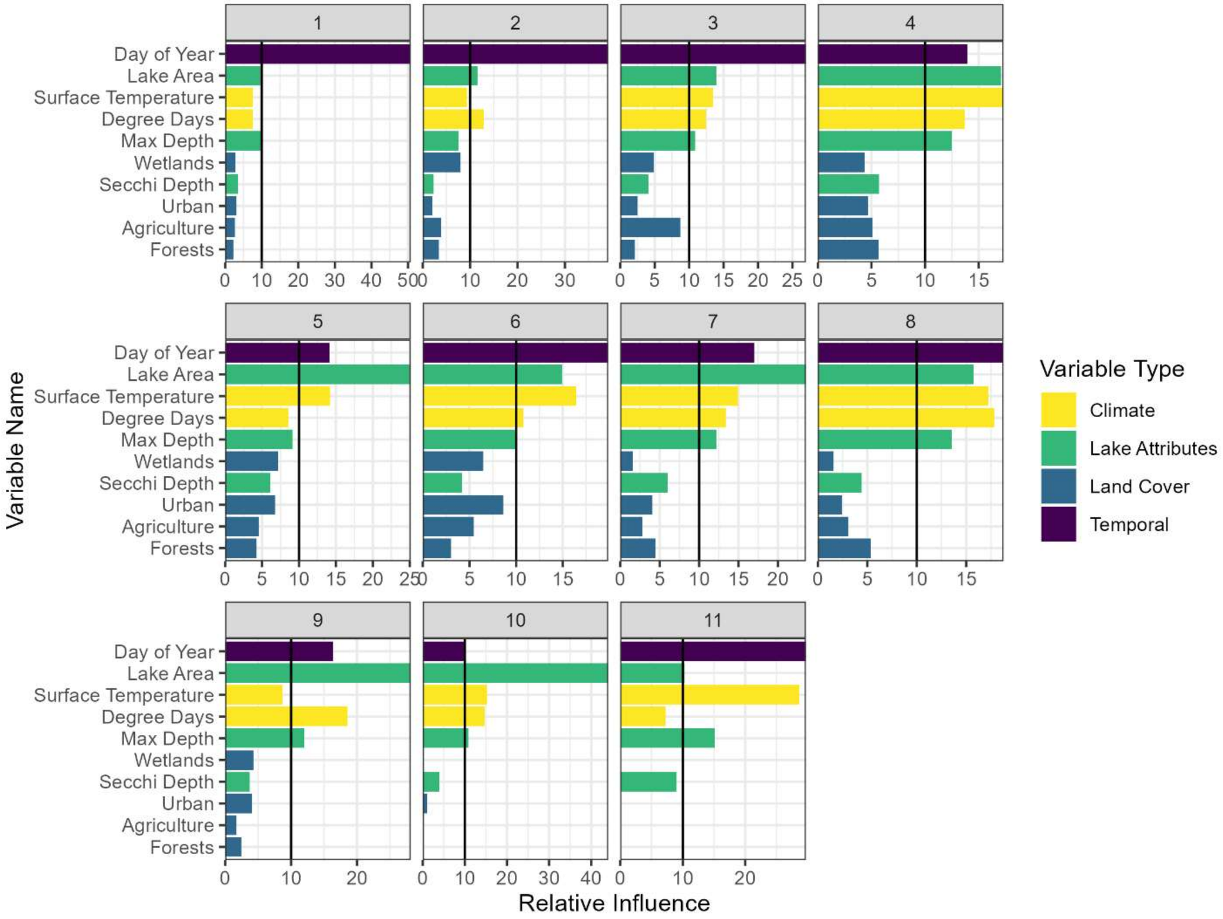

Figure S28. Partial dependency plots for predictors from boosted regression trees for Black Crappie per age class.

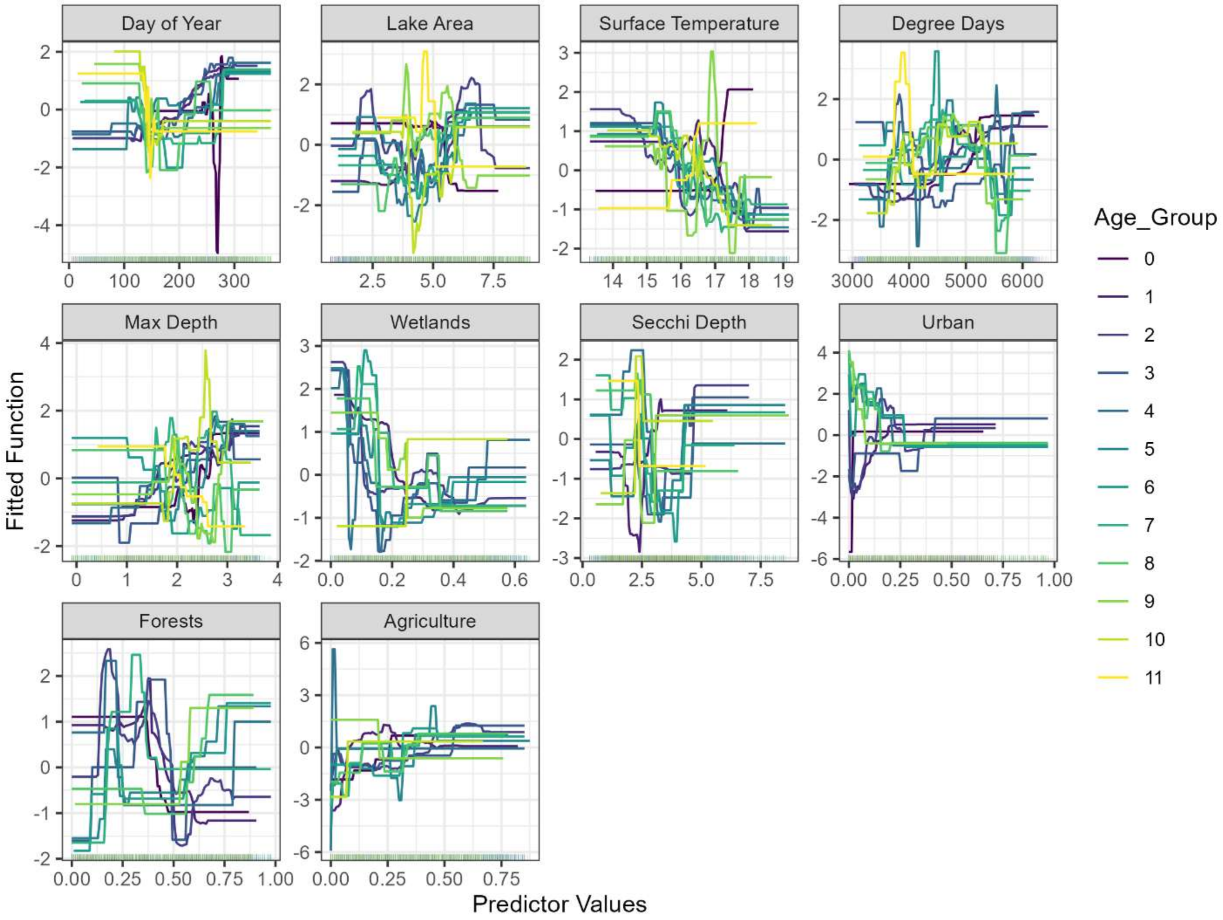

Figure S29. Relative influence of predictors from boosted regression trees for Rock Bass per age class.

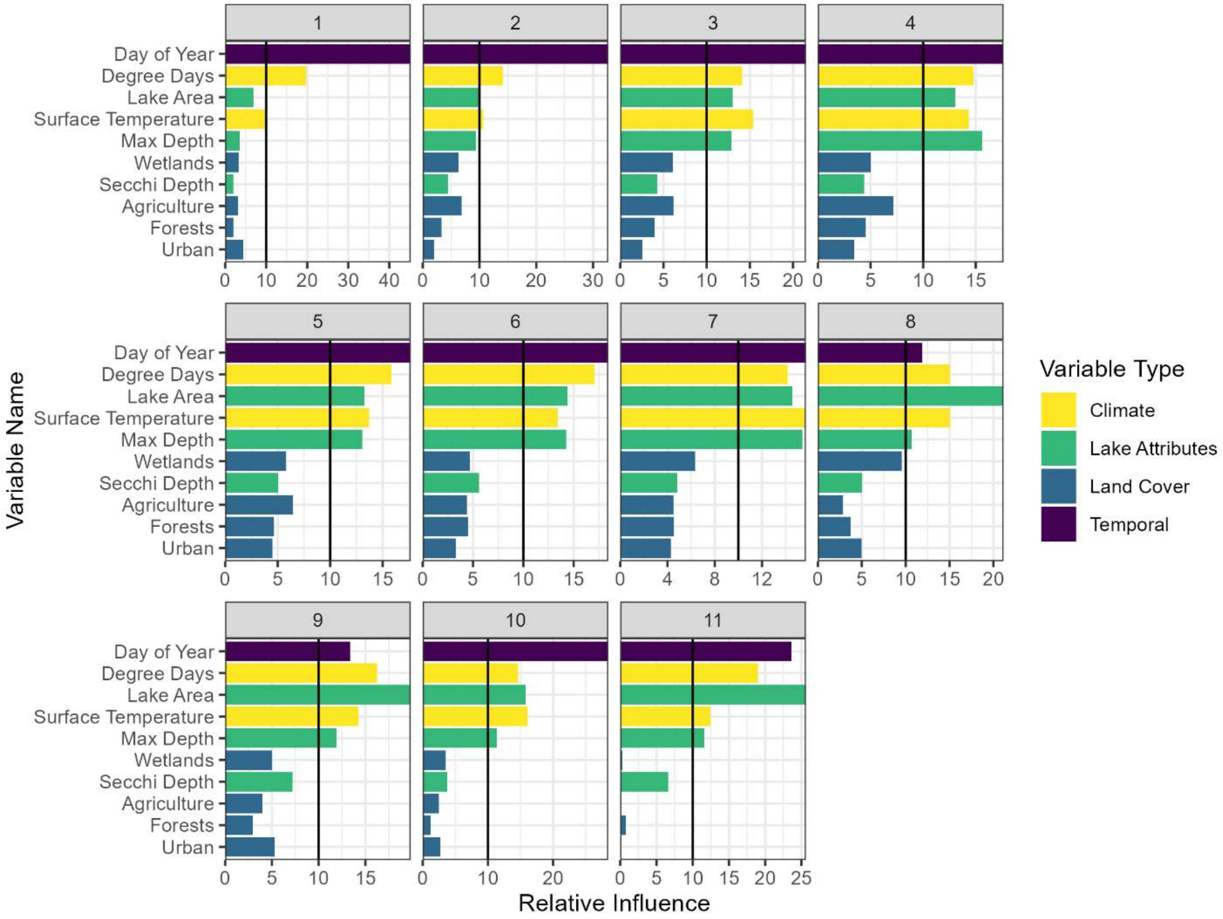

Figure S30. Partial dependency plots for predictors from boosted regression trees for Rock Bass per age class.

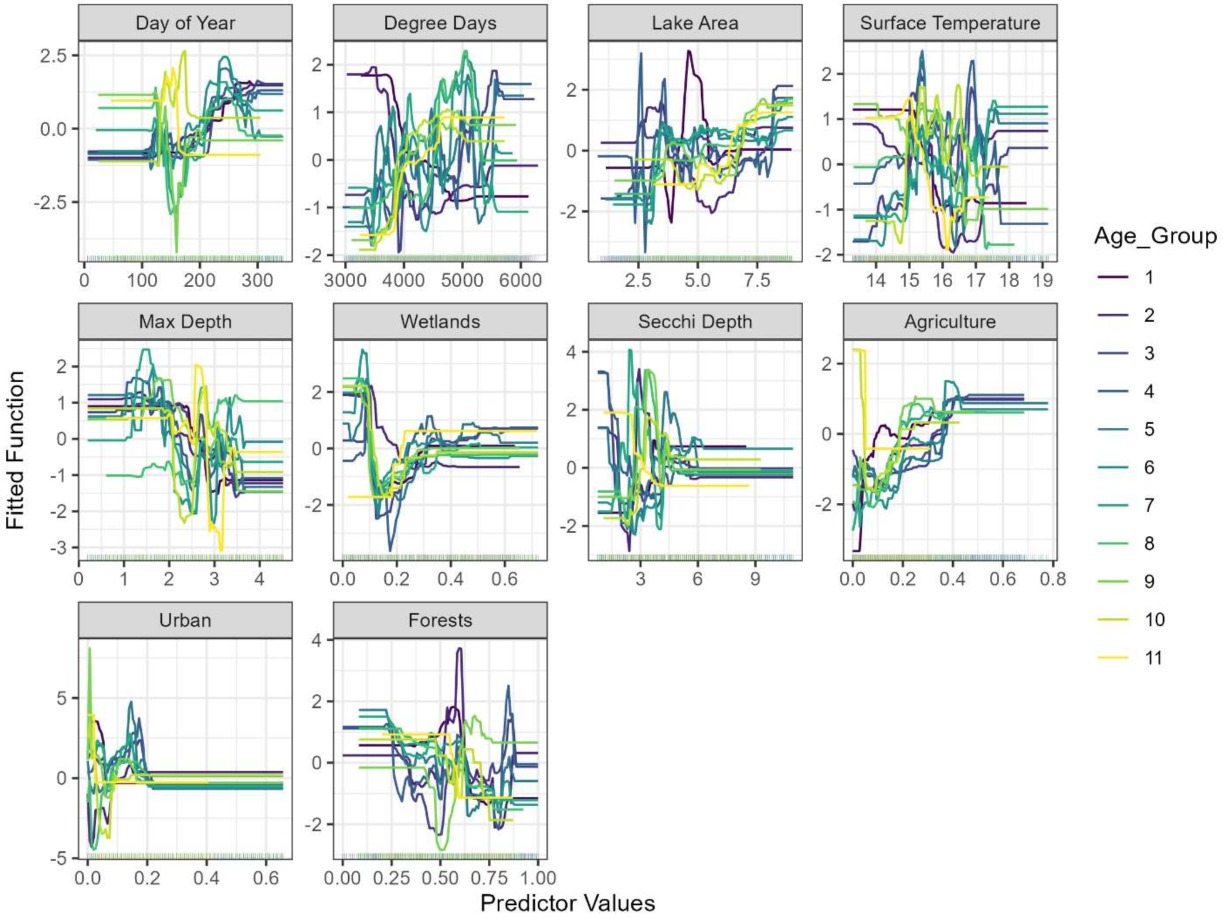

Figure S31. Relative influence of predictors from boosted regression trees for Smallmouth Bass per age class.

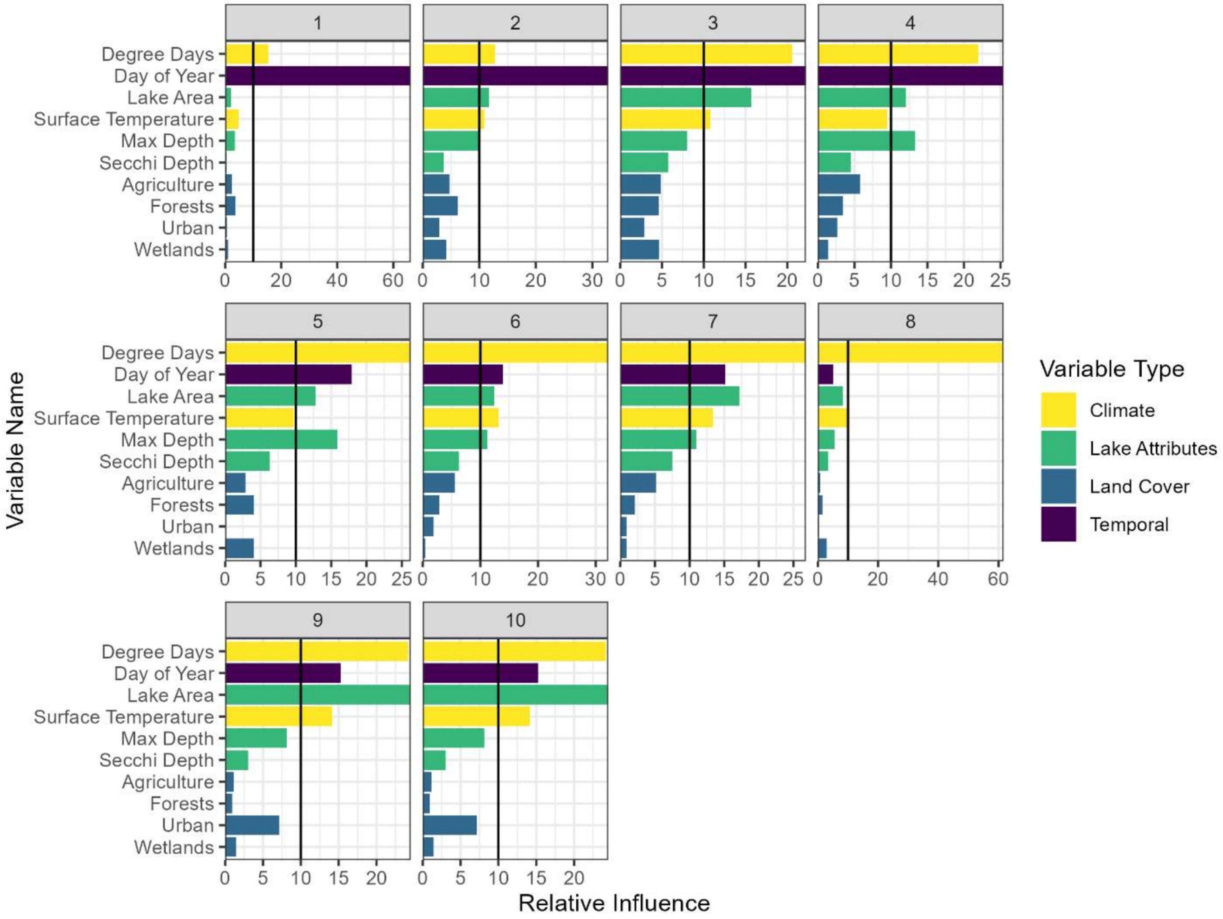

Figure S32. Partial dependency plots for predictors from boosted regression trees for Smallmouth Bass per age class.

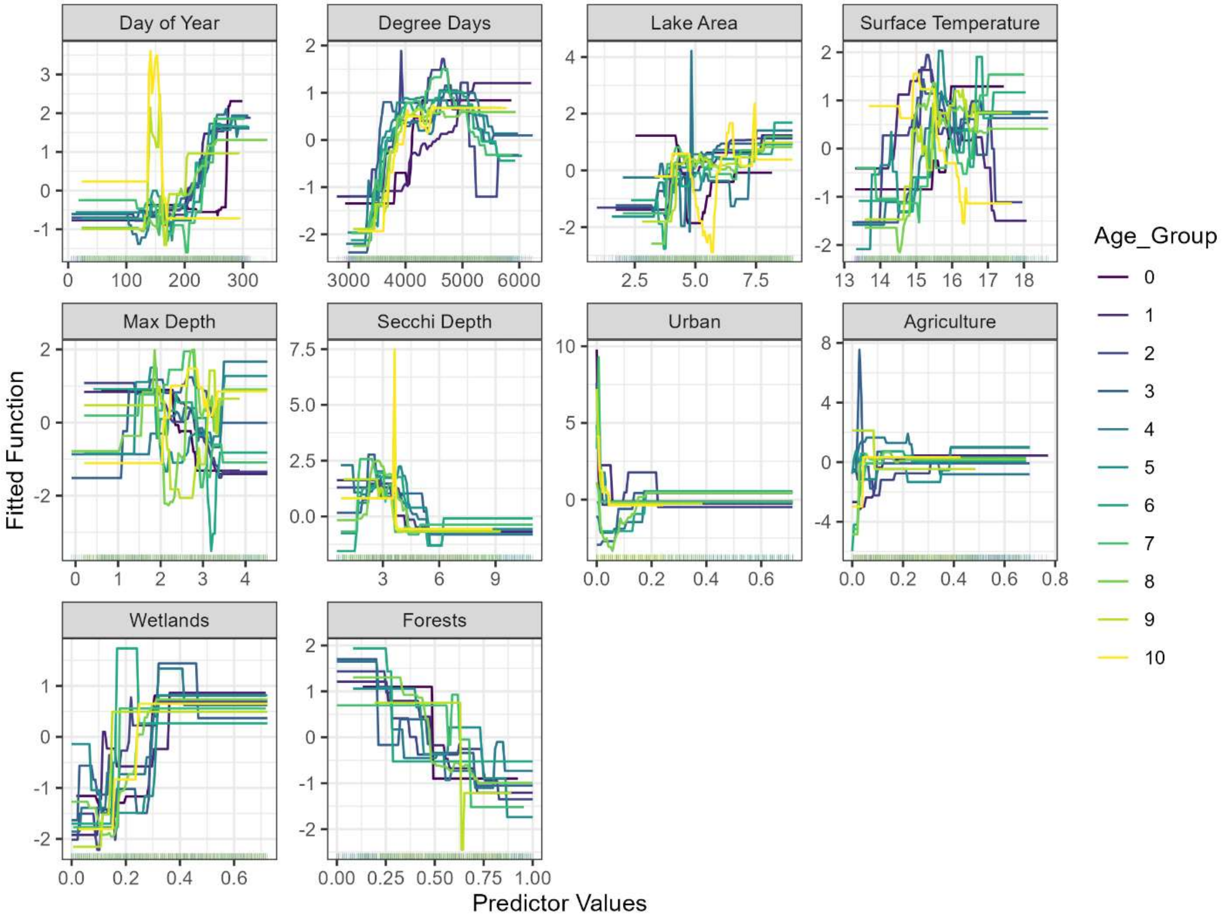

Figure S33. Relative influence of predictors from boosted regression trees for Pumpkinseed Sunfish per age class.

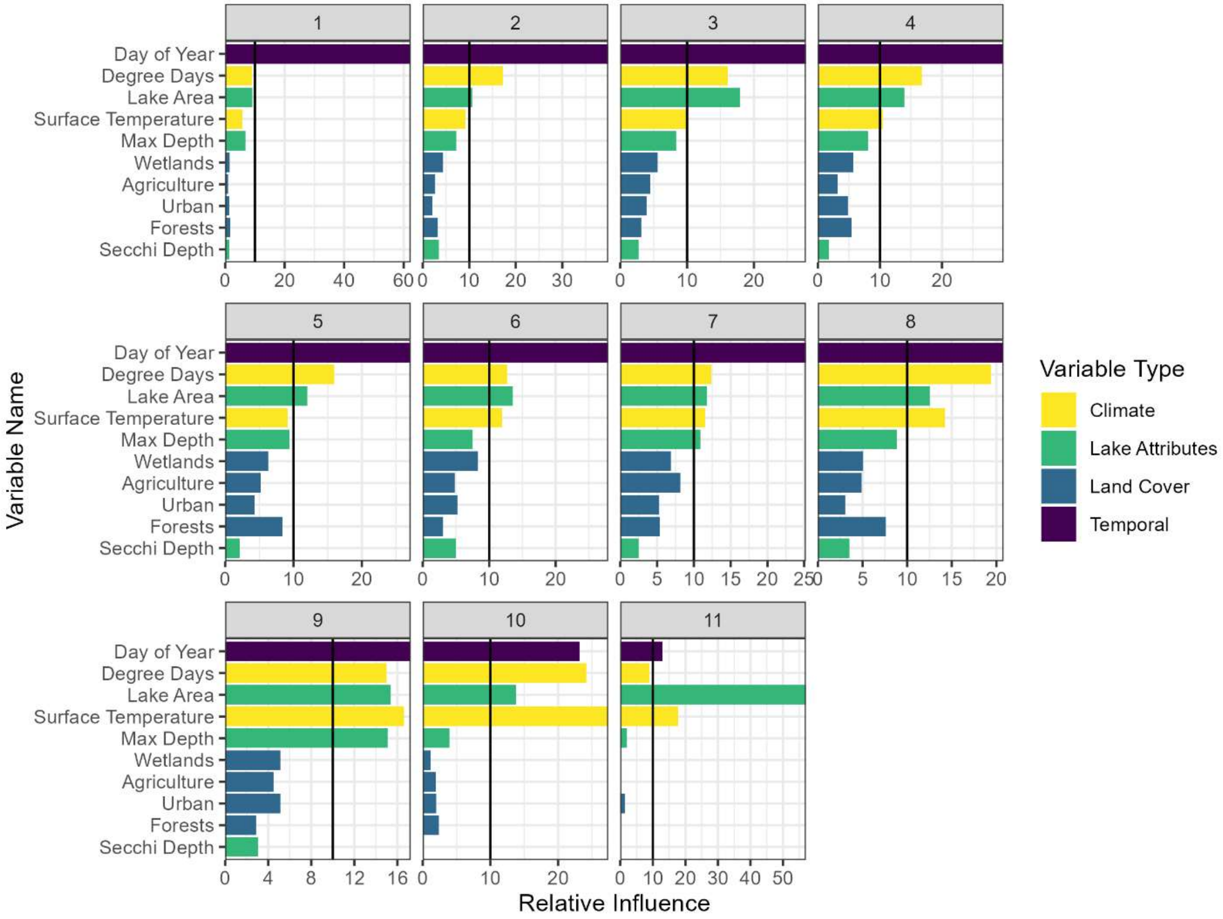

Figure S34. Partial dependency plots for predictors from boosted regression trees for Pumpkinseed Sunfish per age class.

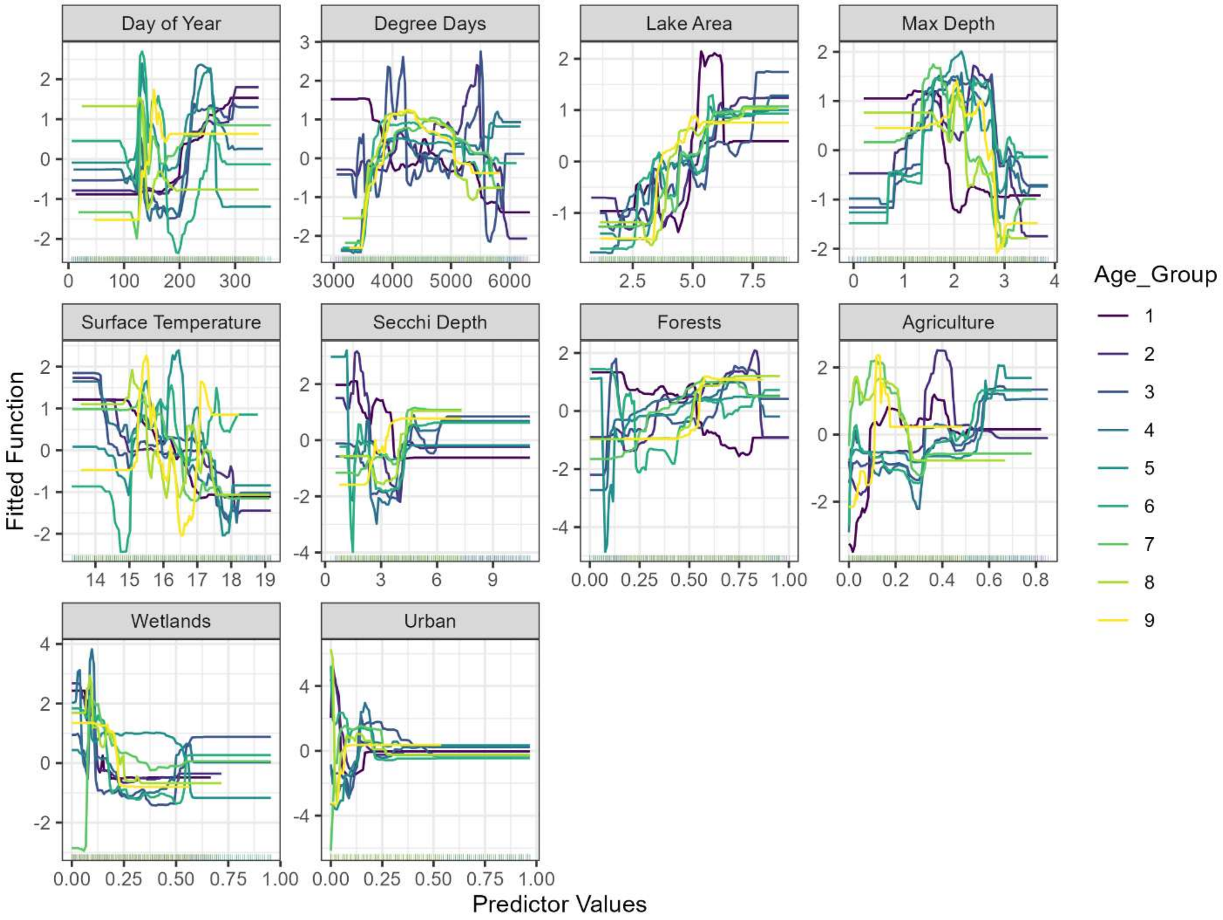

Figure S35. Relative influence of predictors from boosted regression trees for Largemouth Bass per age class.

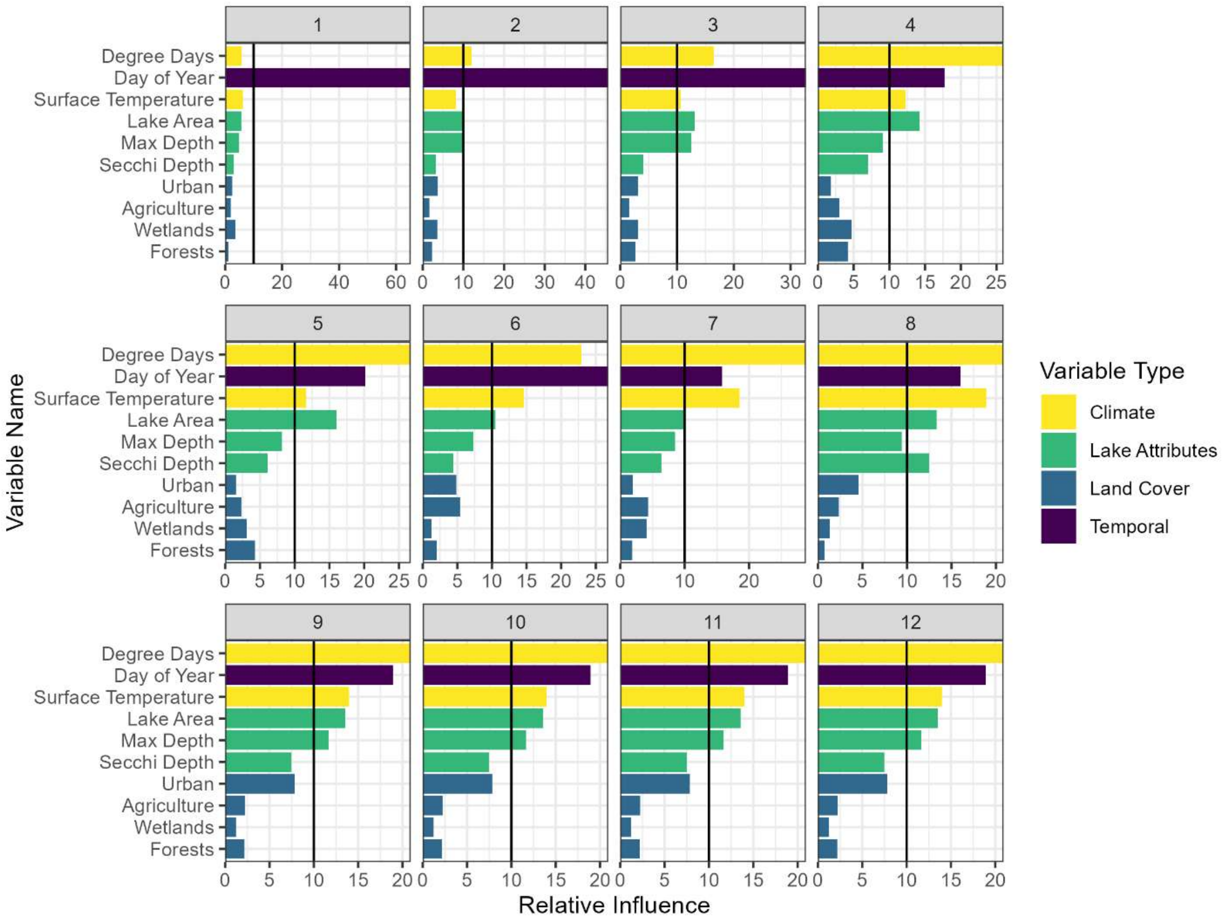

Figure S36. Partial dependency plots for predictors from boosted regression trees for Largemouth Bass per age class.

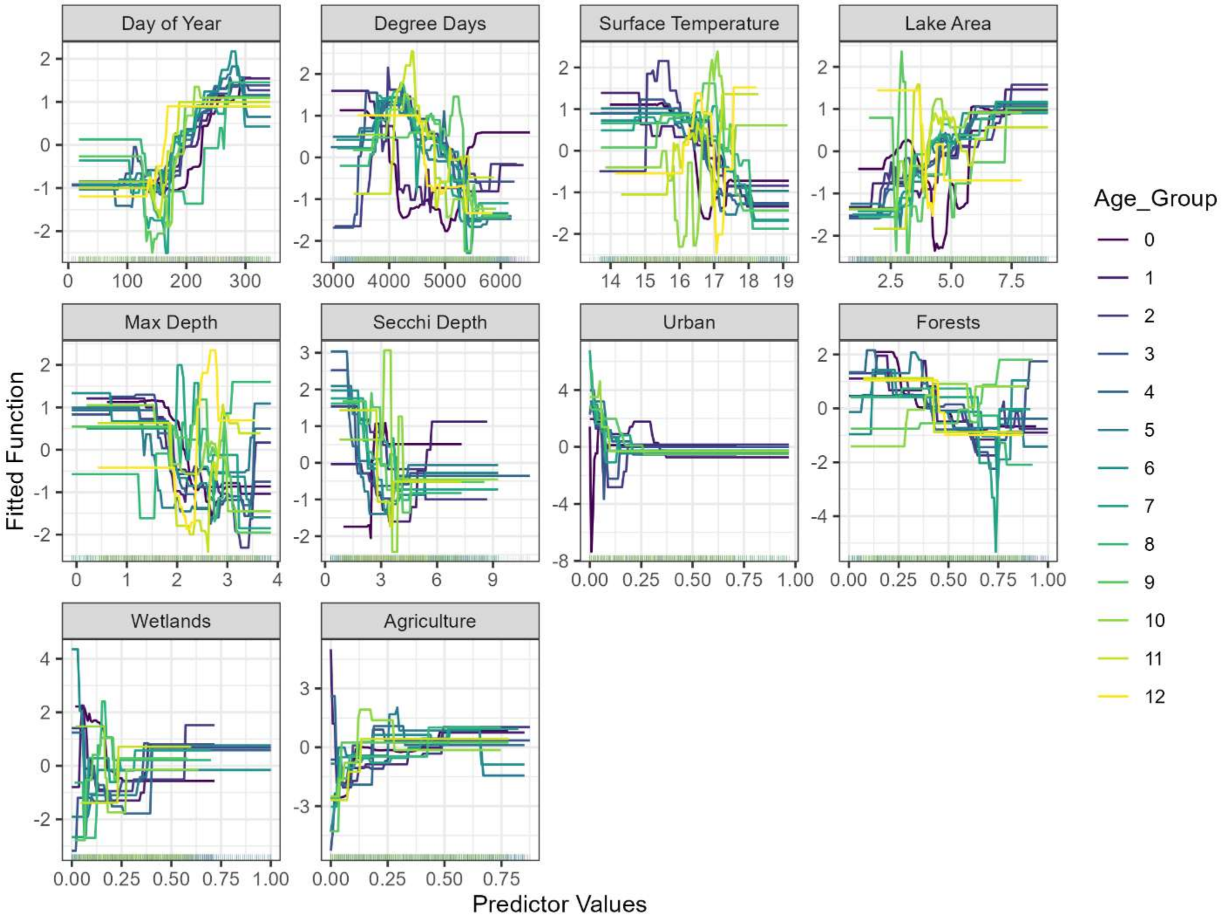

Figure S37. Relative influence of predictors from boosted regression trees for Bluegill per age class.

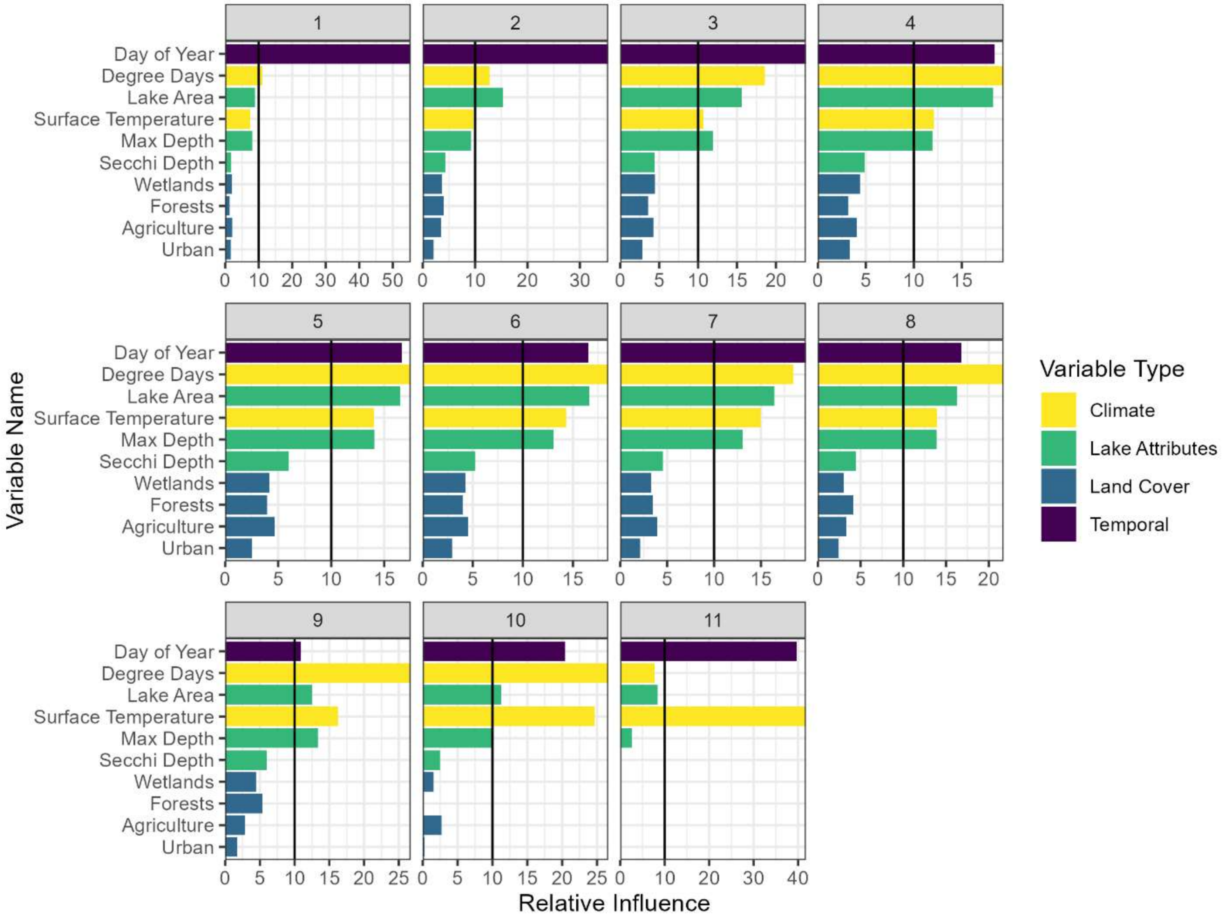

Figure S38. Partial dependency plots for predictors from boosted regression trees for Bluegill per age class.

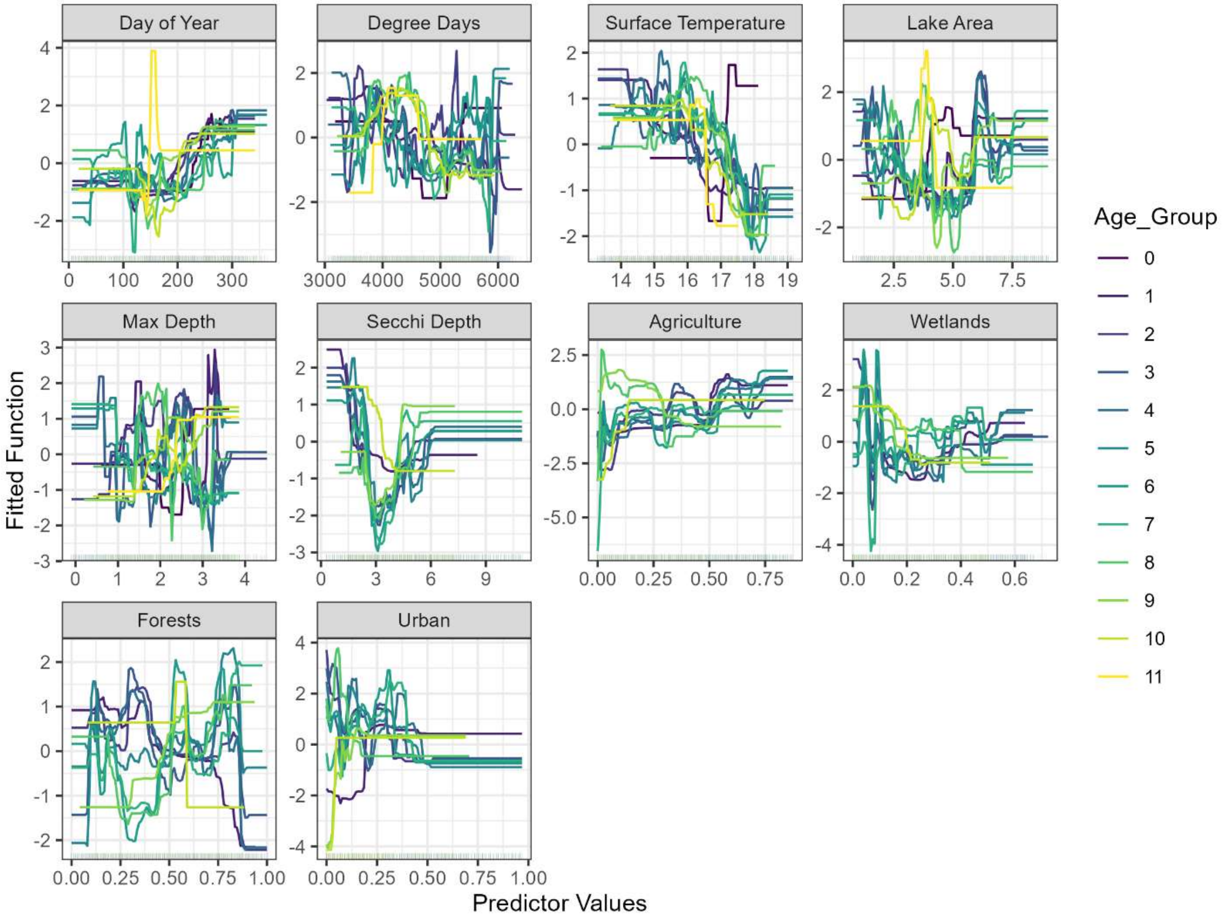

Figure S39. Trends in our data for A) mean annual lake surface temperature, B) annual growing degree days, C) mean annual growing degree days during the lifetime of each age class, and D) latitude. Trendline is a GAM fit with a 95% confidence interval in shaded gray. Gray points mean that the color/fill variable was missing (NA) for that data point.

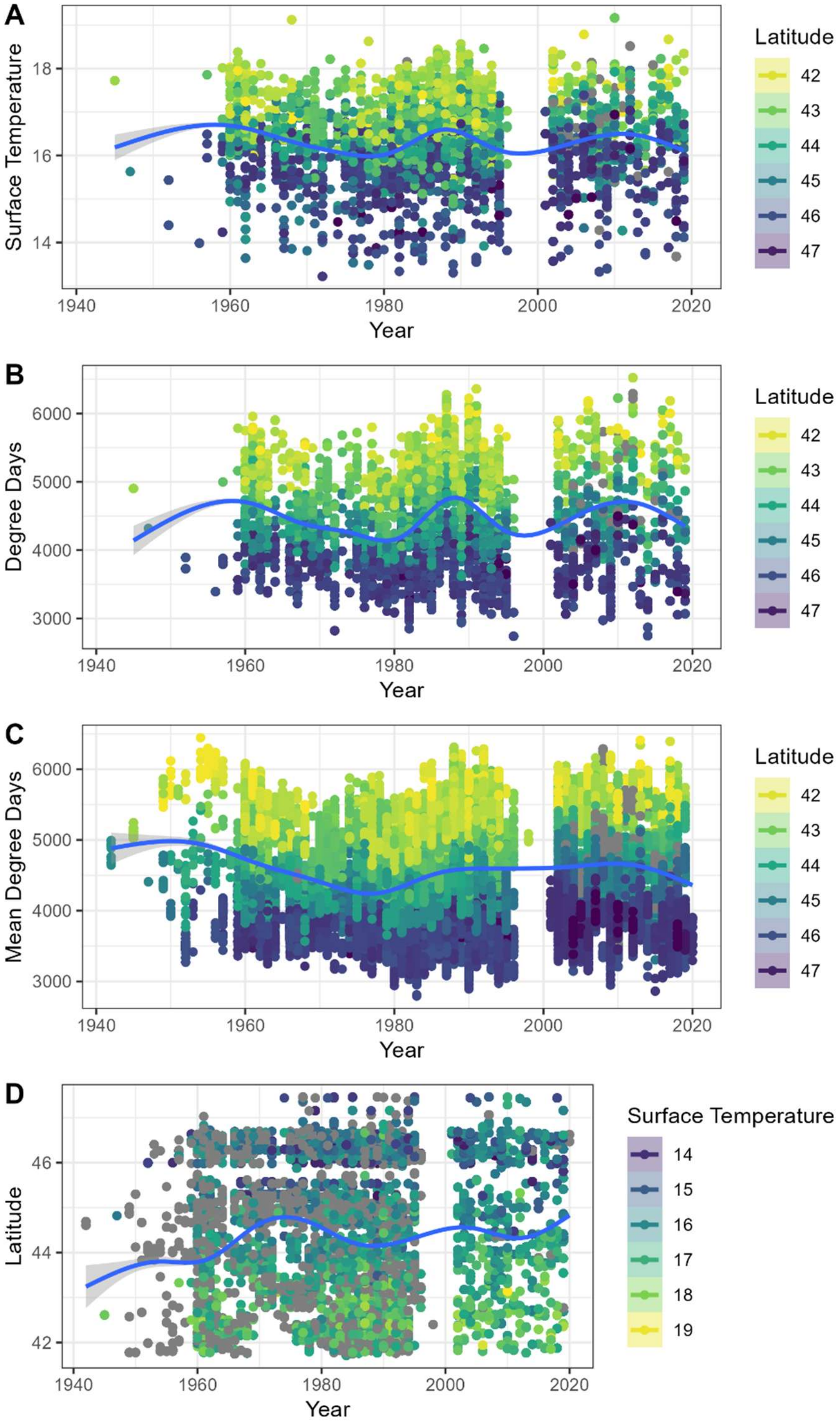

Supplement: Supplementary file 1 — Appendix S1: gcb70584‐sup‐0001‐AppendixS1.pdf. [file GCB-31-e70584-s001.pdf]
